# Immobilisation versus early active motion following primary flexor pollicis longus tendon zones 1–2 repair: protocol for a registry-based multicentre randomised controlled trial in Sweden

**DOI:** 10.1136/bmjopen-2026-118506

**Published:** 2026-10-20

**Authors:** Maria Wilcke, Annika Elmstedt, Erika Nyman, Jonas Svingen, Gustav Andersson, Eva Lundqvist, Sara Edsfeldt, Marianne Arner

**Affiliations:** 1 Department of Hand Surgery, Södersjukhuset, South General Hospital, Stockholm, Sweden; 2 Department of Clinical Science and Education, Karolinska Institutet, South General Hospital, Stockholm, Sweden; 3 Department of Hand Surgery, Plastic Surgery and Burns, Linköping University Hospital, Linköping, Sweden; 4 Department of Orthopedic and Hand Surgery, Faculty of Medicine and Health, Örebro universitet, Örebro, Sweden; 5 Department of Diagnostics and Intervention, Hand- and Plastic Surgery, University Hospital of Umeå, Umeå university, Umeå, Sweden; 6 Department of Medical and Translational Biology, Umeå University, Umeå, Sweden; 7 Department of Surgical Sciences, Uppsala University, Department of Orthopaedics and Hand Surgery, Uppsala University Hospital, Uppsala, Sweden

**Keywords:** Hand & wrist, Orthopedics, Orthopaedic & trauma surgery, REGISTRIES, Randomized Controlled Trial

## Abstract

**Introduction:**

Flexor pollicis longus (FPL) tendon injuries in the thumb are surgically challenging and require careful postoperative rehabilitation to avoid complications such as tendon rupture, stiffness and impaired hand function. Rupture rates after FPL repairs are substantially higher than those reported for finger flexor tendons. Although controlled early active motion (EAM) is widely used following FPL repairs, rehabilitation protocols developed for finger flexor tendons may not be directly transferable to the thumb because of differences in anatomy, biomechanics and functional demands. This study investigates whether immobilisation for 4 weeks postoperatively can reduce rupture rates without compromising functional outcomes after FPL injuries.

**Methods and analysis:**

This registry-based randomised controlled unblinded superiority trial will be conducted across five academic hand surgery centres. Patients aged 15 years or older with a complete FPL injury in zone 1 or 2 will be included. Participants will be randomised 1:1 to either the EAM group, in which training starts 2–5 days postoperatively with protection by a dorsal cast and a volar removable splint or the casted immobilisation group for 4 weeks. After 4 weeks, the same rehabilitation protocol will be introduced for both groups. The primary outcome is the frequency of tendon rupture over 12 months. The secondary outcomes include patient-reported outcome measurements and patient-reported satisfaction, active range of motion of metacarpophalangeal and interphalangeal joints, grip and key pinch strength at 3 and 12 months postoperatively. All data are collected within the Swedish National Quality Registry for Hand Surgery. The primary outcome will be analysed on an intention-to-treat basis using a stratified analysis (stratified by centre), with the absolute risk difference and relative risk reported with 95% CIs. Secondary outcomes will be analysed using generalised estimating equations and standard tests for continuous and ordinal data, with results presented as effect estimates with 95% CI.

**Ethics and dissemination:**

The trial was approved by the Swedish Ethical Review Authority (2025-00730-02). All participants are asked to provide written informed consent. The results will be disseminated as published articles in peer-reviewed journals.

**Trial registration number:**

NCT06836349.

**STRENGTHS AND LIMITATIONS OF THIS STUDY**

- This registry-based randomised controlled trial compares cast immobilisation with controlled early active motion following primary zone 1–2 flexor pollicis longus tendon repair.
- Embedding the trial within the Swedish National Registry for Hand Surgery enables multicentre recruitment, methodologically consistent collection of outcome measurements and enhances generalisability to clinical practice.
- A prespecified sample size calculation based on registry data ensures the study is adequately powered, while shared rehabilitation protocols and validated patient-reported outcome measures ensure methodological consistency across participating centres.
- Blinding of participants and outcome assessors is not feasible because of the nature of the interventions, and as in routine clinical practice, adherence to rehabilitation protocols cannot be fully monitored.
- As in routine clinical practice, surgical repair techniques and suture materials are not standardised across centres, and some secondary outcomes are assessed by treating, unblinded hand therapists, introducing a potential risk of detection bias.

## Introduction

### Background and rationale

 Rehabilitation after flexor tendon injuries of the fingers is critical to prevent tendon adhesion and joint stiffness.^1 2^ Controlled early active motion (EAM) is typically initiated a few days after surgery, and for repairs of the flexor tendon, EAM has produced better range of motion compared with cast immobilisation.^1 2^ The foremost risk associated with EAM is tendon rupture.^3^ Tendon rupture often requires reoperation, leading to increased patient suffering and additional healthcare costs.^4^ Moreover, outcomes following tendon re-repair are generally inferior to those of primary repairs.^3 4^

Most studies on flexor tendon injuries focus on the finger flexor tendons, while injuries involving the flexor pollicis longus (FPL) have been less extensively studied.^5 6^ Interestingly, the rupture rate for FPL repairs has been reported to be as high as 17% compared with approximately 3% for the flexor digitorum profundus repair.^7–10^

Although EAM is widely implemented following FPL repair,^3 9 11^ it may be associated with higher re-rupture rates^7–10^ and immobilisation could hypothetically represent a safer alternative. Concerns have been raised that rehabilitation protocols developed for finger flexor tendons may not be directly transferable to FPL repairs because of anatomical and biological differences, including greater muscle shortening, increased repair tension, and differences in tendon vascularity.^12^ Conversely, EAM has been advocated to reduce tendon tethering, adhesion formation and loss of interphalangeal (IP) joint motion following FPL repair.^12 13^ Therefore, the optimal postoperative rehabilitation strategy after FPL repair remains unclear and no studies have directly compared immobilisation with EAM specifically after FPL repair.

### Objectives

This study aims to compare the rupture rate after zones 1–2 FPL primary repair between patients who are immobilised for 4 weeks in a cast with patients who are issued a removable protective splint and instructed in controlled EAM 2–5 days postoperatively. The primary hypothesis is that the immobilised group will have a statistically significantly lower rupture rate.

The secondary hypotheses are that patient-reported symptoms and disability and measurements of active range of motion and grip and pinch strength will not differ between groups at 3 and 12 months postoperatively.

### Trial design

This study protocol is for an unblinded registry-based randomised controlled trial (RRCT) with two parallel groups, allocation ratio 1:1, using a superiority design.

## Methods: participants, interventions and outcomes

This protocol follows the Standard Protocol Items: Recommendations for Interventional Trials (SPIRIT).^14^

### Study setting

In Sweden, most patients with flexor tendon injuries have surgical repair done at one of the seven national hand surgery centres. These patients are registered and monitored through the Swedish National Hand Surgery Quality Register (HAKIR).^15^ Patient postsurgical follow-up is done using online follow-up questionnaires via the national healthcare platform 1177^16^ prior to surgery and again postoperatively at 3 and 12 months of patients’ self-reported symptoms, functional impairment and satisfaction. In addition, the participating centres routinely perform extended follow-up by hand therapists at 3 and 12 months postoperatively for patients undergoing flexor tendon repair, including assessments of range of motion and grip strength.

The study will be conducted at five hand surgical centres in Sweden:

- Department of Hand Surgery, South General Hospital, Stockholm.
- Department of Hand Surgery, Plastic Surgery and Burns, Linköping University Hospital.
- Department of Hand and Plastic Surgery, University Hospital of Northern Sweden, Umeå.
- Department of Orthopaedics and Hand Surgery, Uppsala University Hospital.
- Department of Orthopaedics and Hand Surgery, Örebro University Hospital.

All participating centres are secondary or tertiary referral hospitals and use the extended HAKIR extended follow-up programme for flexor tendon repairs.

The first author (MW) serves as the principal investigator of the study. At each participating centre, a hand surgeon who participated in the study design will serve as co-principal investigator. All surgeon co-principal investigators have clinical and research expertise in surgical management of flexor tendon injuries and hand surgery. At each centre, a hand therapist with clinical experience in hand rehabilitation and flexor tendon injuries serves as the local lead hand therapist, coordinating rehabilitation delivery and training of participating therapists. Screening of patients referred for FPL injuries at each centre is the responsibility of the co-principal investigator, in collaboration with delegated study staff.

### Participants

Any patient referred to the trial centres for a suspected zone 1–2^17^ FPL injury will be screened for eligibility. Table 1 outlines the inclusion and exclusion criteria. Patients with concomitant digital nerve injuries involving the thumb or other digits, as well as partial tendon injuries in the fingers of the same hand, are eligible for inclusion in the study.

**Table 1:** Inclusion and exclusion criteria

Inclusion criteria
Complete zone 1–2 FPL laceration.
Primary repair within 14 days of injury.
Age ≥15 years. Patients under 18 years of age will be assessed by the treating surgeon or co-principal investigator as having sufficient maturity to understand the study information, procedures and implications and to provide informed consent.
Ability to communicate in spoken and written Swedish.
Exclusion criteria
Tendon injury requiring tendon grafting.
Complete tendon injuries other than the FPL in the same hand.
Associated injuries such as fractures, vascular injuries impairing circulation, skin defects requiring reconstruction with a skin graft or flap or preoperative signs of infection.
Observable conditions such as cognitive impairment, severe psychiatric illness or ongoing substance abuse that may impair the patient's ability to safely participate in and adhere to the EAM rehabilitation programme.
Wound infection developing between postoperative days 2 and 5, thereby contraindicating the EAM rehabilitation programme.

EAM, early active motion; FPL, flexor pollicis longus.

### Intervention

This trial will compare two different methods for managing patients after zones 1–2 FPL repair. These methods include either (1) immobilisation of the wrist and thumb in a dorsal cast from operation until 4 weeks postoperatively (immobilisation group) or (2) wrist and thumb dorsal cast and a volar removable thermoplastic splint and implementation of controlled EAM (splint/EAM) within 2–5 days after surgery (splint/EAM group). At 4 weeks postoperatively, participants in both groups will be given the same restrictions and rehabilitation programme. A standardised rehabilitation protocol (online supplemental material tables 1-4) is delivered by licensed occupational therapists or physiotherapists (hand therapists) at each participating centre. Both groups will attend in-person visits at 2–5 days postoperatively (for a cast check or fabrication of a splint), 2 weeks (suture removal), 4 weeks (transition to the shared rehabilitation protocol) and 12 weeks; further appointments are scheduled when clinically indicated. Based on surgeon or hand therapist assessment, analgesics and additional appointments according to the individual patient needs are permitted when clinically indicated. In the event of an FPL rupture or other adverse event such as a postoperative infection, the need for reoperation or other intervention will be assessed by the responsible surgeon in consultation with the participant to determine the need for further treatment.

### Measurement of outcomes

The primary outcome variable is the frequency of FPL tendon rupture over a 12-month period. Tendon rupture is defined as complete loss of active IP joint flexion, confirmed by direct visualisation of tendon discontinuity at reoperation. If the participant declines reoperation, the diagnosis will rely on this clinical criterion alone, which is a recognised source of possible misclassification. Tendon rupture may occur and be identified at any point during the 12-month follow-up period, rather than only at the scheduled 3-month and 12-month assessments; participants are instructed to contact their hand surgery centre if a rupture is suspected between scheduled visits (see adverse events and safety), and the date of clinical presentation or reoperation is recorded as the event date for the primary analysis. The full schedule of assessments for both groups is illustrated in figure 1.

**Figure 1:**
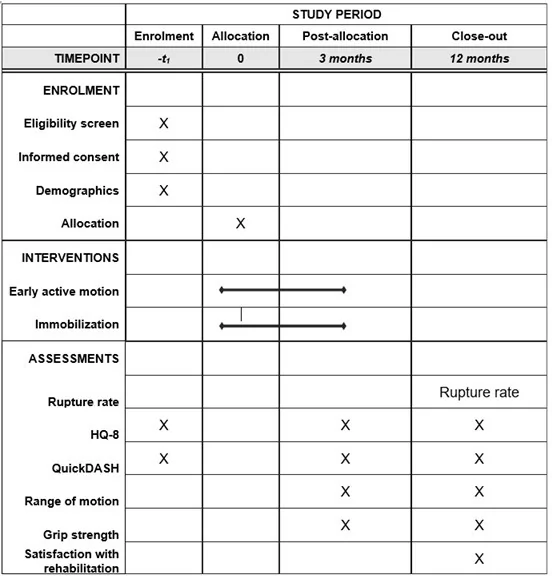
SPIRIT diagram of enrolment, interventions and outcome measures. HQ-8, Swedish National Hand Surgery Quality Register (HAKIR) Questionnaire-8; QuickDASH, Quick Disabilities of the Arm, Shoulder and Hand; SPIRIT, Standard Protocol Items: Recommendations for Interventional Trials.

Secondary outcome variables include active range of motion, grip and pinch performance measurements and patient-reported outcome measures (PROMs).

The treating therapist will measure the outcomes and enter the data as described in the HAKIR English measurement manual as follows:

- Goniometric bilateral measurement of active range of motion of the thumb metacarpophalangeal (MCP) and IP joints, measured in isolation at each joint. Active extension and flexion of the MCP and IP joints will be measured at the 3 and 12-month assessments using a standard finger goniometer placed over the dorsal aspect of each joint. Active range of motion of the MCP and IP joints will be presented in degrees and as a percentage of the contralateral side.
- Jamar dynamometer or equivalent hydraulic hand dynamometer will be used to measure grip strength and pinch gauge Jamar or equivalent for key pinch strength. The mean of three consecutive measurements will be recorded. Dynamometers are calibrated according to the manufacturer’s instructions prior to the study and at regular intervals thereafter to ensure comparability of measurements across centres. Grip and key pinch strength are measured at the 3 and 12-month assessments and reported in kilograms and as a percentage of the contralateral side.

PROMs will be collected with online follow-up questionnaires, via the national healthcare platform 1177^16^ and include:

- HAKIR symptom survey (HQ-8)^18^ is a patient-reported Numerical Rating Scale (NRS) with 11 response options ranging from 0 to 100 in increments of 10, with a higher score indicating worse symptoms. HQ-8 is administered three times; preoperatively and at 3 and 12 months postoperatively. Symptoms surveyed include pain on loading, pain during unloaded motion, pain at rest, weakness, stiffness, cold sensitivity, numbness and activities of daily life experienced during the previous week. This instrument has been validated for the Swedish hand surgery population.^18^ The minimal clinically important difference (MCID) has not yet been established for this instrument.
- The Quick Disabilities of the Arm, Shoulder and Hand (QuickDASH)^19^ is an 11-item self-assessment of symptoms and disability in the upper limb, scored from 0 (no disability) to 100 (most severe disability). QuickDASH is administered three times; preoperatively and at 3 and 12 months postoperatively. MCID is considered to be a difference of approximately 8–15 points.^20^
- Patient satisfaction with the surgical outcome is assessed in HAKIR at 12 months postoperatively using an 11-point NRS, with response options ranging from 0 to 100 in increments of 10. Higher scores indicate greater satisfaction.^15^ An MCID has not been established for this HAKIR-specific item.
- Five-point Likert scale of overall satisfaction^21^ with rehabilitation, assessed 12 months postoperatively and ranging from 1 (very dissatisfied) to 5 (very satisfied). An MCID has not been established for this instrument.

### Sample size

The sample size calculation was performed a priori for the primary outcome of tendon rupture. Based on previous HAKIR data, the expected rupture rate was estimated at 10% in the splint/EAM group and 3%^8^ in the immobilisation group, corresponding to an absolute risk reduction of seven percentage points. The primary hypothesis was that 4 weeks postoperative immobilisation would result in a lower rupture rate compared with 4 weeks of postoperative splint/EAM rehabilitation, consistent with a superiority design. Based on a two-sided significance level of 0.05 and 80% statistical power, a total of 194 patients per group was required. However, HAKIR captures only surgically treated tendon ruptures, and the present study also includes clinically diagnosed ruptures managed non-operatively. According to a previous study based on HAKIR data, up to one-third of clinically confirmed flexor tendon ruptures did not undergo reoperation.^10^ Because the overall event rate was expected to be higher than the HAKIR-based estimates, the number of evaluable patients required to detect the same absolute difference was correspondingly lower; a conservative target of 173 evaluable patients per group was calculated. Allowing for up to 10% loss to follow-up, a final recruitment target of 190 patients per group was set.

### Participant recruitment

Eligible patients at participating centres will be invited to participate in the study. The principal investigator will monitor recruitment progress at all centres through regular review of HAKIR recruitment reports. Recruitment rates, protocol adherence and any barriers to enrolment will be discussed at steering committee meetings, allowing potential challenges to be identified and addressed in a timely manner. An overview of the trial design is presented in figure 2, and the enrolment, intervention schedule and outcome measures are illustrated in figure 1.

**Figure 2:**
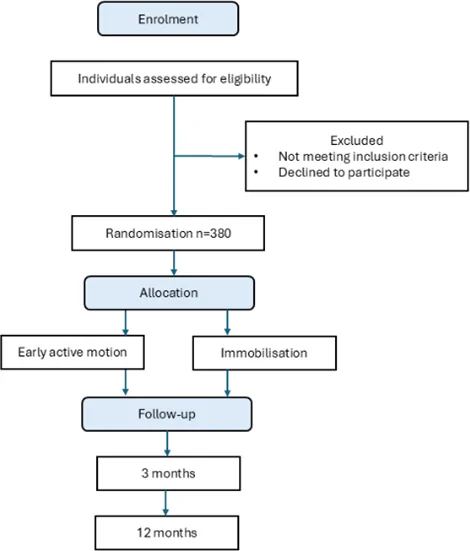
Flow chart of the trial design.

### Study status

Protocol version 2.1 dated 29 January 2025. The first participant was recruited on 13 June 2025. At the time of manuscript submission, 21 participants had been enrolled. Recruitment is ongoing.

### Adherence

Participants will attend routine follow-up visits according to the rehabilitation protocol at each participating centre. During scheduled follow-up visits, the hand therapist or research staff will remind participants to complete the PROM questionnaires in HAKIR and assist with technical issues if needed. Completion of PROM questionnaires and attendance at follow-up visits will be recorded in HAKIR.

Participants who miss scheduled appointments will be contacted by phone, electronic message or mail and offered a new appointment. Reasons for withdrawal or discontinuation will be documented when available. Participants who withdraw before randomisation or before initiation of the allocated rehabilitation protocol will be replaced.

Adherence to the splint/EAM group will be assessed by the treating hand therapist during follow-up visits based on participant self-report and clinical evaluation. Participants allocated to the immobilisation group will be instructed to maintain cast immobilisation continuously for 4 weeks; unlike the splint/EAM programme, adherence to continuous cast-wearing cannot be objectively verified between visits. Participants requiring post-trial treatment will be offered the centre’s standard care for FPL injuries. Those who suffer harm by trial participation may seek compensation from the Swedish patient insurance system (LÖF).

## Methods: assignment of interventions

### Randomisation and blinding

Randomisation will be conducted after signed informed consent is obtained by either the dedicated surgeon, hand therapist or research nurse. A prerandomised allocation sequence, stratified by participating centre, was generated within the HAKIR registry infrastructure prior to study initiation. Each participating centre has its own allocation list. For each new participant, the HAKIR randomisation function assigns the next allocation in the pregenerated randomisation sequence. Participants will be randomly allocated 1:1 to either the immobilisation group or the splint/EAM group, within each centre, with a block size of four to six. There will be no blinding of participants, treating therapists or outcome assessors because of the nature of the interventions.

## Methods: data collection, management and analysis

### Baseline participant and injury assessment

Baseline data including age, sex, injured side, smoking, mechanism of injury, injury of dominant hand, comorbidities (ie, diabetes or hand osteoarthritis) are collected by the hand surgeon, hand therapist or research nurse after inclusion. Participants complete the baseline PROMs with instructions to report on their status using the week before the injury as a reference point. The surgeon enters information about the zone FPL tendon injury, associated digital nerve injury, surgical technique and core suture configuration within the flexor tendon injury HAKIR assessment.

### Outcome data collection

Data include the number of FPL tendon ruptures, PROMs, MCP and IP joint thumb range of motion, grip and pinch strength of injured and contralateral hands and adverse events (ie, postoperative infection) collected as required by the HAKIR registry, 3 and 12 months postoperatively (figure 1).

Participants are allowed to withdraw from the trial at any time. In the event of participant withdrawal, data collection is discontinued and the participant’s data up to the time of withdrawal will be used and analysed.

In the event of an FPL rupture noted by either clinical exam and/or confirmed intraoperatively, the participant will remain included in the study and assessed as planned at 3 and 12 months after the primary repair operation.

### Data management

All data will be entered directly into the HAKIR registry and stored under the participant’s personal identification number. When analyses are to be performed, the researchers will receive a pseudonymised data file from the registry.

### Statistical plan

Descriptive statistics will be presented as means with SD for normally distributed continuous variables. Normality will be assessed using Q-Q plots. Non-parametric and continuous variables that are not normally distributed will be presented as median with IQRs. Categorical variables will be presented as numbers with percentages.

The primary outcome (rupture rate) will be analysed according to the intention-to-treat principle using a Mantel-Haenszel (MH) analysis stratified by centre, reflecting the centre-stratified randomisation and chosen for its robustness to sparse strata. The pooled relative risk (RR) and risk difference (RD) will be estimated with the MH estimators, with splint/EAM as the reference group. For the RD, centres are weighted approximately in proportion to their size; for the RR, the weights also depend on the number of ruptures in the splint/EAM group. Larger centres, and for the RR centres with more ruptures in the reference group, will therefore have greater influence on the pooled estimates. The 95% CIs will be based on the Greenland-Robins variance estimator for the log RR and the Sato variance estimator for the RD, both of which are valid for sparse strata.^22 23^ No continuity correction is required, centres with no ruptures in one group contribute to both estimates, whereas centres with no ruptures in either group contribute to the RD (as a difference of zero) but not to the RR. If no ruptures occur in one group across all centres, only the RD will be reported. No covariates other than centre are included in the primary analysis: centre is prespecified as the sole adjustment factor because it is the randomisation stratification variable, and adjustment is not based on observed baseline imbalance between groups, consistent with Consolidated Standards of Reporting Trials (CONSORT) 2025 guidance. Should a clinically important baseline imbalance be identified after unblinding, an additional analysis adjusting for the relevant characteristic(s) will be reported as a prespecified sensitivity analysis, separate from the primary analysis.

Consistency of the treatment effect across centres will be examined descriptively by presenting centre-specific numbers of ruptures and RDs with 95% CI in a forest plot. Given five centres and a low expected event rate, no formal test of treatment-by-centre interaction will be performed.

Secondary outcomes, including PROMs, active range of motion and grip and pinch strength, will be analysed using the independent-samples t-test for normally distributed data and the Mann-Whitney U test for non-normally distributed and ordinal data. Results will be presented with 95% CI intervals. This includes ordinal and satisfaction measures without an established MCID (HQ-8, NRS satisfaction and the five-point Likert satisfaction scale), which will be statistically compared between groups irrespective of MCID availability. In addition, generalised estimating equations, adjusted for age, sex, baseline PROM and centre will be used for analysis of repeated secondary outcome measurements. Centre will be included as a fixed covariate to account for between-centre variation, while data from all participating centres will be analysed together within a single combined model rather than separately by centre. Robust variance estimation will be applied to account for within-subject correlations in repeated measurements. The working correlation structure will be selected according to the lowest Quasi-likelihood under Independence Model Criterion, and linear models will be used for all variables.

Missing data for the primary outcome will not be imputed, as tendon rupture represents a clearly defined clinical endpoint and the primary analysis will be based on observed data only. For secondary outcomes, missing data will be imputed using multiple imputation with predictive mean matching, applying a donor pool size of k=5, as part of sensitivity analyses. The imputation models will include outcome measures at relevant follow-up time points, as well as participant age, sex, smoking status, injury of the dominant hand, suture technique and core suture configuration.

Analyses requiring stratified MH estimation, predictive mean matching with a specified donor pool, or group-sequential alpha-spending boundaries will be performed in R. Remaining analyses will be performed in SPSS or R.

### Analysis

The trialist responsible for data analysis will remain blinded to treatment allocation. The blinded results will be presented to the writing committee (ie, the authors) for consensus on interpretation prior to unblinding.

### Interim analysis and stopping guidelines

One interim analysis of the primary outcome (rupture rate) will be performed after approximately 50% of the participants have been enrolled and completed the 12-month follow-up. Early stopping for efficacy will be guided by a group-sequential design using an O’Brien-Fleming alpha-spending function to preserve the overall two-sided type I error rate at 0.05. The interim efficacy boundary corresponds approximately to p≤0.003. The final analysis will use a significance threshold of p≤0.05.

Early termination for harm will be considered if a statistically significant difference (ie, decrease or increase) in rupture rates is observed between the groups at the interim analysis. Decisions regarding early stopping will be made by the steering committee after review of the blinded interim results. The interim analysis is designed to detect a large treatment effect and will have approximately 80% power to detect an absolute RD of 13%.

An interim analysis of secondary outcomes, including PROMs, active range of motion and grip and pinch strength, will be conducted after 20 participants per group have completed 12 months of follow-up. This analysis is intended to assess safety, feasibility, and measures of range of motion and strength, and will primarily be descriptive. Formal hypothesis testing of secondary outcomes at interim will not be used to guide early termination.

For active range of motion in the IP-joint, non-inferiority will be assessed using a predefined margin of 20°. The 20° margin was chosen based on clinical judgement regarding what would constitute a meaningful difference in thumb IP-joint motion. Based on registry data from HAKIR, assuming an SD of 24° at 12 months postoperatively, a sample size of 19 participants per group provides 80% power to detect non-inferiority at a one-sided significance level of 0.05. These analyses will be considered supportive and exploratory.

### Adverse events and safety

Serious adverse events are defined as events leading to hospitalisation or death that are directly attributable to the intervention. For this trial, two rehabilitation programmes are implemented, both designed to protect the FPL repair. Possible adverse events may include FPL rupture noted by examination or intraoperative confirmation and postoperative infection which may require hospitalisation. Any tendon rupture or infection will be recorded. If an adverse event occurs outside scheduled follow-up visits, participants will be instructed to contact their hand surgery centre. All participating centres provide on-call hand surgery services and are equipped to manage adverse events. Study participants experiencing an adverse event will be managed according to standard clinical practice, irrespective of trial participation. The participant and the surgeon responsible will determine whether reoperation is indicated and the centre will provide appropriate treatment.

### Monitoring

There will be no formal external monitoring of this trial. All participating centres are responsible for the oversight and monitoring of the study at their centre. The Steering Committee oversight consists of the principal investigator (MW), lead on-site investigators (ie, hand surgeons) at each centre, the registry holder and the National Coordinator of HAKIR. The collaborative Research Team includes hand therapists and research nurses at the participating centres who are responsible for the practical implementation of the trial. The Steering committee and the Research Team will meet regularly as required, with meetings held monthly to every third month, depending on needs, to ensure consistent study conduct, protocol adherence, patient recruitment, data quality and coordination between centres. All study-related information is available on the HAKIR website (HAKIR | Handkirurgiskt kvalitetsregister).

## Ethics and dissemination

### Ethical considerations

This study complies with the ethical guidelines stated in the Declaration of Helsinki and has received approval from the Swedish Ethical Review Authority on 13 November 2023 (D.nr. 2023-04922-01) and 6 March 2025 (D.nr. 2025–00730-02). Significant protocol amendments that may impact the conduct of the study will only be implemented following approval from the Swedish Ethical Review Authority. The trial has been prospectively registered in ClinicalTrials.gov (NCT06836349) on 25 February 2025.

In Sweden, the capacity of minors to provide informed consent is assessed based on maturity and understanding rather than age alone; this approach, including the consent procedures for participants aged 15–17 years, has been reviewed and approved by the Swedish Ethical Review Authority as part of the study protocol.

Both rehabilitation programmes implemented in this study are established and routinely used in clinical practice to manage surgically repaired FPL injuries. Participants will therefore not be exposed to experimental or unproven interventions. The main potential risk associated with the study is tendon rupture, which is a known complication after FPL surgical repair regardless of what rehabilitation postoperative programme is used. Additional risks include secondary thumb joint stiffness and reduced grip and pinch strength. These risks will be clearly communicated to patients before participating in the study.

Benefits of participation may include a potential reduced risk of tendon rupture, possible improvement in functional outcomes. The study does not generally involve more frequent follow-up than standard care; however, because the rehabilitation programmes have been standardised within the study, some participants may require more frequent follow-up visits during the rehabilitation period compared with usual practice at their treating centre. All participants will receive standard postoperative care and monitoring, and any complication will be managed as outlined, according to established clinical practice. Participation in the study will not affect access to appropriate medical treatment.

### Information of the trial and approval of the participant

All patients will be given written and verbal information before signing the informed consent. Only patients who provide written informed consent (online supplemental material) will be entered into the study. Informed consent will be obtained by a surgeon, research nurse or hand therapist, immediately after surgery or at the first outpatient clinic appointment 2–5 days after surgery. Both groups attend this first outpatient visit: participants allocated to the splint/EAM group begin training at this visit, while participants allocated to the immobilisation group have their cast checked and continue immobilisation. At that visit, the study’s purpose, methods, potential benefits and risks will be explained. Patients can decline or withdraw their consent at any time during the study. Informed consent will be recorded in both the medical record and the HAKIR registry.

### Confidentiality

All participant data will be handled in accordance with the General Data Protection Regulation (GDPR) and applicable Swedish data protection legislation. Study data will be collected and stored within HAKIR using each participant’s personal identification number. As the study data are stored within the national quality registry HAKIR, it will not be destroyed after study completion, in accordance with Swedish legislation governing national quality registries. Access to identifiable data will be restricted to authorised personnel involved in registry administration. For research purposes, the study team will receive pseudonymised datasets in which personal identifiers have been replaced with unique study codes. The key linking study codes to personal identifiers will be stored securely within HAKIR and will not be accessible to the Steering Committee or Research team. All analyses will be performed on pseudonymised data, and results will be reported at an aggregated level to prevent individual identification. Participant information will be stored on secure servers with appropriate technical and organisational safeguards.

### Patient and public involvement

Patients and the public were not involved in the design, conduct, reporting or dissemination plans of this trial as the interventions are uncomplicated and constitute standard care following FPL injuries. The study was designed by clinicians and researchers based on existing clinical evidence and HAKIR registry data.

### Communication of results, authorship and access to data

The findings of this study will be published in peer-reviewed journals. Results will be presented through scientific posters or oral presentations at relevant conferences and meetings. Results will be shared with the participants involved through the HAKIR website. The researchers will have full access to the final trial dataset. Data generated and analysed during this study are stored within HAKIR and are subject to Swedish data protection legislation and the GDPR. Due to legal and ethical restrictions, individual-level participant data cannot be made publicly available. Access to de-identified study data may be granted to qualified researchers on reasonable request and subject to approval by the Swedish Ethical Review Authority and the HAKIR Steering Committee. Access to the study protocol, including the statistical analysis plan described herein, and analytic code will be granted after reasonable request. Requests should be directed to the corresponding author.

## Discussion

Controlled EAM is widely used after FPL repair, largely extrapolated from evidence in digital flexor tendon repair, where EAM is associated with reduced adhesion formation and joint stiffness compared with immobilisation.^5 6^ Reported rupture rates following FPL repair have varied across studies, with some series reporting higher rates than those observed after repair of the digital flexor tendons.^7–10^ However, no study has directly compared immobilisation and EAM following zones 1–2 FPL repair.^7–9^ Consequently, the relative effects of these rehabilitation strategies on tendon rupture, functional recovery and patient-reported outcomes remain uncertain. This RRCT was designed to address this knowledge gap by comparing 4 weeks of cast immobilisation with splint/EAM following primary zones 1–2 FPL repair.

The primary outcome is tendon repair rupture, while active MCP and IP joint active motion, grip and pinch strength, and PROMs are assessed as secondary outcomes at 3 and 12 months postoperatively. Tendon rupture was selected as the primary outcome because it frequently necessitates reoperation, delays rehabilitation and may be associated with poorer functional recovery, satisfaction and higher levels of pain.^3 4^ The secondary outcomes were chosen to capture both clinical and patient-reported aspects of recovery and to facilitate comparison with previous studies of flexor tendon repair. Together, these outcomes will provide a comprehensive assessment of both structural and functional recovery following zone 1–2 FPL repair.

An important asset of this study is the integration of an RCT within a national quality registry (RRCT design). This design facilitates large-scale recruitment and may reduce the burden on surgeons and therapists, as patient recruitment, follow-up and outcome assessment are embedded within the registry’s existing systems, while retaining the external validity of routine clinical practice. However, this pragmatic approach may provide less control over treatment delivery and follow-up procedures than a traditional explanatory RCT.

Several limitations should be considered. For this study, surgeons use their preferred repair materials and techniques. In doing so, this reflects routine clinical practice and enhances external validity. However, variation in surgical technique, suture configuration and repair strength may influence both rupture risk and functional outcomes. Previous case series of FPL repair have demonstrated declining rupture rates with progressively stronger multistrand core suture repairs when combined with EAM,^5 6^ suggesting that repair technique is an important determinant of safety. Consequently, surgical factors may contribute to variation in outcomes both within and between centres.

For this study, tendon rupture is defined as loss of active IP-joint flexion, confirmed by direct visualisation of tendon discontinuity when reoperation is performed; when a clinically suspected rupture is not surgically confirmed, the diagnosis relies on this clinical criterion alone, which may reduce specificity, since impaired IP-joint flexion caused by tendon adhesions could be misclassified as rupture.

The unblinded design may introduce performance and assessment bias, as surgeons, therapists and participants are aware of treatment allocation, and outcome assessors are typically the same therapist responsible for the participant’s rehabilitation. Although rehabilitation protocols were developed collaboratively by hand surgeons and therapists across all participating centres to promote consistency, delivery may still vary between therapists, centres and individual participants, potentially contributing to outcome variability.

The secondary outcomes, including active range of motion and strength measurements, are more susceptible to detection bias, because they are assessed by the treating hand therapist rather than an independent assessor and some measurement variability is unavoidable despite calibrated instruments and a shared assessment protocol. PROMs may also be affected by reporting bias, as participants are aware of their treatment allocation; although validated questionnaires are used, this bias cannot be excluded.

Interim analyses, described in the Methods, are intended primarily to protect participant safety rather than to guide early efficacy claims, and use conservative statistical thresholds to limit the risk of false-positive findings.

As this trial does not standardise surgical repair technique, its findings should be interpreted as applicable to routine Swedish hand surgery practice rather than extrapolated broadly. Factors such as repair technique, injury zone and patient adherence may also contribute to rupture risk and will be described in secondary analyses where feasible. If immobilisation is associated with lower rupture rates without compromising functional or patient-reported outcomes, this may inform postoperative practice; conversely, if splint/EAM achieves comparable rupture rates while maintaining or improving recovery, current practice may be further supported. Either outcome is expected to provide clinically relevant evidence to guide postoperative management following FPL repair.

## Supplementary material

10.1136/bmjopen-2026-118506 online supplemental file 1

10.1136/bmjopen-2026-118506 online supplemental file 2
