# Supplementary material for: Immobilisation versus early active motion following primary flexor pollicis longus tendon zones 1–2 repair: protocol for a registry-based multicentre randomised controlled trial in Sweden
Source: BMJ Open. 2026 Oct 20;16(10):e118506. doi: 10.1136/bmjopen-2026-118506 (PMC13684173; doi:10.1136/bmjopen-2026-118506)
Supplement: Supplementary file 1 [file bmjopen-2026-118506.reviewer_comments.pdf]

PEER REVIEW HISTORY

BMJ Open publishes all reviews undertaken for accepted manuscripts. Reviewers are asked to complete a checklist review form and are provided with free text boxes to elaborate on their assessment. These free text comments are reproduced below.

ARTICLE DETAILS

Title (Provisional)

Immobilisation versus Early Active Motion Following Primary Flexor Pollicis Longus Tendon Repair: Protocol for a Registry-Based Multicentre Randomised Controlled Trial in Sweden.

Authors

Wilcke, Maria; Elmstedt, Annika; Nyman, Erika; Svingen, Jonas; Andersson, Gustav; Lundqvist, Eva; Edsfeldt, Sara; Arner, Marianne

---

VERSION 1 - REVIEW

**Reviewer**	**1**
**Name**	**Marson, Ben**
**Affiliation**	**University of Nottingham, Trauma Outcomes Group**
**Date**	**23-Apr-2026**
**COI**	**None**

A robust study protocol. Good luck.

**Reviewer**	**2**
**Name**	**Howell, Julianne W.**
**Affiliation**	**Self-employed Hand Therapy Consultant**
**Date**	**13-May-2026**
**COI**	**None**

Authors, your research question is important as it may add to our knowledge in the rehabilitation management of FPL repairs. Please review the attached specifics outlined by this reviewer. Please write from a rehab not surgical perspective.

**ClinicalTrials.gov NCT06836349**	**Reviewer Comments**
**Title:**<br>**A Registry- Randomized Controlled Trial Comparing Rehabilitation Regimens Following Flexor Pollicis Longus Tendon Repair (REFLEX): Study protocol**	Title-<br>Authors-Consider striking (REFLEX) from title as it seems unnecessary, striking regime and update terminology with programs or approaches, and adding Randomized Controlled Trial with a superiority design
**Abstract:**<br>**Introduction**	**Abstract:**<br>**Introduction**

Ln 7-8 Flexor pollicis longus (FPL) tendon injuries in the thumb are surgically challenging and require careful postoperative rehabilitation to avoid complications such as tendon rupture, stiffness, and impaired hand function.	Ln 7- 8 Authors- consider tendon adherence and edit and substitute FPL throughout abstract
Ln 9-13 Controlled Early active motion (EAM) is the standard protocol for flexor tendon repairs. However, rupture rates after flexor pollicis longus repairs are significantly higher than for finger tendons. ~~Given the anatomical and functional characteristics of the thumb, it is unclear whether early mobilisation is ideal following repair of the flexor pollicis longus.~~ This study investigates whether immobilisation for four weeks postoperatively can reduce rupture rates without compromising functional outcomes after flexor pollicis longus tendon injuries.	Ln 9-13 Authors- Please revise this entire section.<br>1. Consider introducing the problem, significantly more FPL than FDP ruptures first, then postoperative rehab programs/regimens.<br>2. It is your opinion that the anatomy and functional use of the thumb are factors that may correspond with higher FPL rupture rates, please remove this from the abstract. You could use this as a discussion point if supported by evidence. There are many other possible contributors to FPL rupture from surgical repair techniques, patient adherence, therapist experience, co-morbidities, mechanism of injury etc.<br>3. Consider why you are comparing 2 different postoperative FPL rehab regimens/program and use this as your rationale. For instance, did another HAKIR -based study suggest this possibility that EAM might be a contributor?
**Methods and analysis**<br>15 -22 This ~~registry-~~randomised controlled unblinded superiority trial will be conducted across five academic hand surgery centres. Patients aged 15 years or older with a complete flexor pollicis longus injury in zone 1 or 2 will be included. Participants will be randomised 1:1 to either the controlled EAM/splint group ~~1) early active motion,~~ starting two to five days postoperatively ~~with a protective splint, or 2~~)or the casted immobilisation group ~~in a cast~~ for four weeks. After four weeks, ~~both groups follow~~ the same rehabilitation protocol will be introduced for both groups.	**Methods and analysis**<br>Ln 15-18 Authors consider the suggested edits<br>1. You have not described the credentials of the hand therapists involved in this study<br>2. Is the cast applied at 2-5 days or intra-operatively?<br>3. Are baseline patient reported outcomes (please name these) done once the patient becomes a participant?
Ln 19-21 The primary outcome is the frequency of tendon rupture over 12-weeks ~~rate~~. The secondary outcomes include ~~patient-reported outcome measurements and patient-reported satisfaction~~, range of motion, and grip strength at three and 12 months postoperatively.	Ln 19-22 Authors- consider edits<br>1. Please specify by name, the patient-reported outcomes, i.e., QuickDASH<br>2. Active or passive ROM, of what joints?<br>3. Grip only or xxx pinch strength?<br>4. Are these measurement of ROM and strength taken by standard methods?
Ln 21-22 All data is collected within the Swedish National Quality Registry for Hand Surgery, HAKIR.	Ln 21-22 Authors please<br>1. specify exactly how data collection will be done (Each therapist enters the data?) and how it will be analyzed

	2. Will there be surgical data also entered and analyzed for this RCT? Please include
**Key words:** Hand & wrist < ORTHOPAEDIC & TRAUMA SURGERY, Orthopedics, Orthopaedic & trauma surgery < SURGERY, REGISTRIES, Radiation oncology < RADIOTHERAPY	**Key words:**<br>Please consider more appropriate key words i.e., flexor tendon, thumb, rehabilitation, immobilization, early active motion, splint
**Introduction:**<br>Lines 32-35 Rehabilitation after flexor tendon injuries of the digits is critical to prevent tendon adhesion and joint stiffness.(reference) Controlled early active motion (EAM) vs. immobilization ~~movement therapy~~ is typically initiated a few days after surgery as EAM has produced ~~since early active mobilisation results in a~~ better range of motion following flexor tendon repair involving the fingers[1] (additional references) ~~compared to immobilisation~~[1]. The foremost risk associated with ~~early active motion~~ EAM ~~training following flexor tendon repair~~ is rupture. ~~due to excessive tendon loading~~.<br><br>Ln 36-~~Strict adherence to postoperative rehabilitation protocols is essential to minimize the risk.~~<br><br>Ln 37 Tendon rupture often requires reoperation, leading to increased patient morbidity and additional healthcare costs. Moreover, outcomes 38 following tendon re-repair are generally inferior to those of primary repairs[2].<br><br>Ln 39 Most studies on flexor tendon injuries focus on finger tendons (Flexor Digitorum Profundus (FDP) and 40 Superficialis (FDS)), while injuries to the thumb flexor tendon (Flexor Pollicis Longus (FPL)) are significantly<br><br>Ln 41 less studied[3]. Notably, the rupture rate for flexor tendon repairs in the thumb is significantly higher than in the 42 fingers. Rupture rates around 3% have been reported in FDP tendon injuries while in thumbs rupture rates up 43 to 17% have been reported[4-6].<br><br>Ln 44 ~~The biomechanics and anatomy of the thumb tendon differ substantially from those of the finger tendons with 45 fewer phalanges, joints, and pulleys, as well as only a single tendon contained within its sheath. Additionally, 46 the functional requirements of the thumb differ from those of the fingers, as its unique role is to perform 47 opposition, which facilitates pulp-to-pulp positioning, pinch, and facilitates precision grips[7] [3].~~	Lines 32-35 Authors please consider suggested revisions.<br>Please seek out additional more current references to support your statement of controlled EAM after finger flexor tendon repairs provides better outcomes than immobilization<br><br>There are other factors that also contribute to tendon rupture, EAM is not the only factor<br><br>Ln 36- consider deletion of this sentence as it implies patient adherence is responsible for tendon rupture. This is your opinion, unsupported by references and how does not support your study intent<br><br>Ln 37-38-Authors please support your statements with references and consider more current references. I am not sure you intended to say patient morbidity = patient death/illness<br>Ln 39- 40 Authors consider this edit-<br>Most studies (REFERENCES) reporting on flexor tendon injury, focus on the flexor tendons of the fingers when compared to those involving the thumb.(REFERENCES)<br><br>Ln 41-43 Authors consider this edit-<br>Interestingly, the rupture rate for the Flexor Pollicis Longus (FPL) has been reported to be up to 17% (REFERENCES) compared to three percent for the Flexor Digitorum Profundus (FDP). (REFERENCES)<br><br>Ln 44-47 Authors this may be a discussion point, not part of the introduction. Please remove<br><br>Ln 48-50 Authors- Please provide all references for controlled EAM rehabilitation programs following FPL repair and support

Ln 48 Although early active mobilisation of flexor FPL injuries is widely implemented it may be associated with higher 49 re-rupture rates8. Therefore, immobilisation could represent a safer alternative. However, evidence remains 50 limited.	your premise that EAM following FPL repair rupture rates are high when compared to FDP EAM rehabilitation programs.<br>One could argue that immobilization may result in less tendon excursion secondary to adhesions and also more susceptible to rupture as the tensile strength of the repair has not been progressively challenged. You state that the evidence is limited, but you have not provided that evidence for the reader to review.
Objectives<br><br>Ln 53-This study aims to determine whether four weeks of postoperative immobilisation reduces rupture rates 54 without adversely affecting objective functional and patients' self-reported outcomes following FPL repair.<br>55 The primary hypothesis is that immobilising the thumb for four weeks postoperatively will reduce the FPL<br>56 rupture rate. Secondary hypotheses are that patient rated symptoms, disability, and satisfaction as well as<br>57 range of motion and grip strength will not differ between immobilisation and early motion at three and 12 58 months postoperatively.	Objectives<br>Authors, please re-consider how you have stated your objectives-<br>1. Comparison between patients with FPL primary repairs who are immobilized for 4 weeks in a cast and patients who wear an thermoplastic splint and initiate controlled EAM motion 2-5 days post-operatively. The authors' hypothesis is that those immobilized will have a statistically significant lower rupture rate, proving immobilization to be superior to EAM.<br><br>2. The secondary hypothesis is that patient report of symptom, disability and strength will not differ between groups when measured at 3 and 12 months post-operatively.
Ln 60-62 **Trial design**<br>61 This study protocol is for an unblinded registry randomised controlled trial ~~(RRCT)~~ with two parallel groups, allocation ratio 1:1, ~~with~~ using a superiority design.	Ln 60-62 Authors please clarify if registry is inserted properly.
**Methods:**<br>Ln 66-This protocol follows the Standard Protocol Items: Recommendations for Interventional Trials (SPIRIT)9.<br><br>**Study setting**<br>Ln 69-73 In Sweden, all patients with flexor tendon injuries have surgical repair done at one of ~~operated at~~ the seven national hand surgery centres. These patients are registered and 70 monitored through the Swedish National Hand Surgery Quality Register (HAKIR)10 [7]. Patient post-surgical follow-up is done using ~~receive~~ online 71 follow-up questionnaires via the national healthcare platform 1177[11] [8] for ~~that assess~~ self-reported symptoms, functional impairment, and satisfaction at three and 12 months postoperatively. For selected diagnoses, including flexor tendon injuries, an extended assessment in	Methods-<br>Authors- consider edits for clarity

which a hand therapist measures range of motion and grip strength is also conducted at three and 12 months postoperatively. ~~during which a hand therapist measures range of motion and grip strength.~~	
Ln 75-79 The study will be conducted at five ~~study~~ hand surgical sites that apply the extended assessment for flexor tendon injuries all hand surgical centres in secondary or tertiary referral hospitals in Sweden: Department of Hand Surgery, 77 South General Hospital Stockholm; Department of Hand Surgery, Plastic Surgery, and Burns, Linköping University Hospital; Department of Hand and Plastic Surgery, University hospital of Northern Sweden, Umeå; Uppsala; Department of Orthopaedics, and Hand Surgery, Örebro University Hospital.	Ln 75-79 Authors- please clarify and consider listing the sites by name, not in paragraph format
Ln80-81 A hand surgeon will serve as the centre's co-principal investigator. Each centre will be responsible for screening all patients referred to the centre for FPL injuries.	Ln 80-81 Authors- who serves as the primary principal investigator and how was the hand surgeon at each centre selected to serve as co-principle investigator. (Important for research method repeatability)
**Participants**<br>Ln 84-85 All patients referred to the trial centres for a suspected FPL ~~flexor tendon~~ injury will be screened for eligibility. The inclusion and exclusion criteria are described in Table 1.	Ln 84-84 Authors consider edits
**Table 1 Inclusion and exclusion criteria**<br>**Inclusion**<br>Complete Zone 1-2 FPL laceration of the FPL ~~within zone 1 and 2.~~<br>Age ≥15 years. Individuals under 18 years old must be deemed sufficiently mature to understand the research implications.<br>Ability to communicate in spoken and written Swedish.<br>Surgery has been performed within 14 days of injury.	**Table 1-**<br>Authors- consider all edits<br>1. Please provide repeatable details regarding how a patient under 18 years is deemed sufficiently mature to understand the research implications<br>2. Primary repair within 14 days of injury<br>Authors- are patients with digital nerve injury, partial tendon injuries of other digits in the same hand included/excluded?
**Exclusion**<br>FPL tendon injury requiring tendon grafting.<br>~~Additional~~ Complete tendon injuries in the same hand.<br>~~Extensive~~ Associated injuries such as fractures, vascular injuries impairing circulation, ~~with circulatory impairment,~~ large skin defects, or preoperative signs of infection.<br>Patients deemed unsuitable for EAM rehabilitation ~~early active motion~~ program ~~training~~ due to non-compliance, cognitive impairment, or substance abuse.<br>Development of wound infection at day two to five postoperatively, contraindicating EAM ~~early active motion~~ rehabilitation program ~~training~~.	3. Define large skin defects<br>4. How do you deem patient will be non-compliant and unsuitable for EAM program?<br>5. Please use consistent updated terminology i.e., EAM rehabilitation program
**Intervention**	

Ln 92-94 This trial will compare two ~~different~~ methods for managing ~~rehabilitation regimes for~~ patients after zones 1-2 FPL injury. These methods include either ~~1)~~ cast immobilization of the wrist, hand and thumb for a period of ~~in a 93 plaster cast~~ four weeks postoperatively (experimental group) or ~~and 2)~~ use of a thermoplastic wrist-hand-thumb splint and implementation of controlled EAM program ~~Early active motion~~ within two to five days after surgery (control group).	**Intervention**<br>Ln 92-94 Authors, please consider more thorough edits<br><br>Authors- when is the cast applied?
Ln 94- At 4- ~~Four~~ weeks after the operation ~~and forward~~, participants in both groups will be instructed in ~~have~~ the same restrictions and rehabilitation program. ~~training regime.~~ For details of the program, see online supplemental material. Analgesics and additional appointments according to the individual needs of the patient are allowed.	Ln 94-96 Authors, please provide<br>1. more details, is the original cast worn for 4 weeks without follow-up? repeatable description of EAM program<br>2. how the need for additional appointments is determined. This protocol should be written to be repeatable by others.<br>3. Please provide the online link for review
Ln 97- 98 In the event of an FPL rupture or other adverse event (i.e., post-operative infection), the participant and the responsible surgeon will decide whether reoperation is indicated, and any required intervention will be performed.	Ln 97-98 please revise for clarity
**Measurement of study outcomes**<br>Ln 101 The primary outcome ~~variable~~ is the frequency of FPL tendon rupture over a 12-week period. ~~rate~~. If an FPL rupture is suspected, the treating therapist will notify the responsible hand surgeon. Clinical assessment for a FPL rupture can only be classified as suspected i.e., no active thumb flexion at interphalangeal (IP) joint. If surgery is pursued, observation intraoperatively will be used to confirm. Note that not all patients may elect to undergo reoperation. ~~If a tendon rupture is confirmed, the surgeon and the 103 participant will decide whether to proceed with reoperation based on standard clinical principles. If the 104 participant is reoperated, the rupture will be confirmed during the operation. If the participant declines 105 reoperation, the diagnosis will be based on loss of active thumb flexion at the interphalangeal (IP) joint.~~<br>106 The secondary outcomes are measured by the treating therapist? At measured at 3 and 12 months postoperatively include- ~~objective function and patient-reported outcomes measurements (PROMs) 107 including:~~<br>• Goniometric measurement of active range of motion of thumb metacarpophalangeal (MCP) and IP joints ~~measured with a goniometer at three and~~	**Measurement of study outcomes**<br>Ln 101-121 Authors, consider edits<br><br>Authors- specify who is taking the measurements. If treating therapist, this may be a limitation

~~109 12 months postoperatively~~ according to the HAKIR measurement manual.10<br>•Jamar dynamometer bilateral grip ~~strength measured with a Jamar dynamometer~~ and key pinch strength ~~with a pinch gauge at 111 three and 12 months postoperatively~~. With the mean ~~value~~ of three consecutive measurements ~~is~~ recorded according to the HAKIR measurement manual10.<br>•HAKIR symptom survey (HQ-8) (reference) including ~~the items~~ pain on loading, pain during motion with no load and pain at rest,<br><br>The patient-reported 11-point numerical rating scale (NRS) 0-100 with a higher score indicating worse symptoms[12], for weakness, stiffness, cold sensitivity, numbness and activities of daily life (ADL) the week before the injury or subsequent assessments. ~~previous week, rated with a self-administered 11-point numerical rating scale (NRS) 0-100, higher score means worse 116 symptoms12.~~	Please describe all methods, not refer reviewer to HAKIR manual unless it is accessible for review
117 • Quick disability of the arm, shoulder, and hand (QuickDASH)[13]. QuickDASH is an 11-item self-assessment of ~~score assessing~~ symptoms and disability rating ~~in the upper limbs and ranges~~ from 0 (no disability) to 100 (most severe disability).	Detail method of scoring, qDASH please<br><br>Was a Jamar pinch gauge used, if not name the brand, please.
119 •The 11-point NRS ~~scale~~[10] of patient verbal? report of overall satisfaction with the surgical outcome. ~~is assessed with a self-administered 11-point NRS (0-100)10 120 three and 12 months postoperatively.~~	Please specify if NRS was verbal report or marked on a scale for all outcomes using NRS
121 • The 5-point Likert scale [14] of overall satisfaction with rehabilitation, ~~on a five-point Likert scale [14] three and 12 months postoperatively~~	
**Sample size**<br>Ln 124-127 The sample size calculation for the ~~primary analysis was based on an expected rupture rate of 10% in the control group and 3% in the intervention group, corresponding to an absolute risk reduction of 7 percentage points. These estimates were derived from previous HAKIR data, which reported reoperation rates due to tendon rupture of 10% following thumb tendon repair and 3% following finger flexor tendon repair5.~~<br><br>~~128 The primary hypothesis was that the intervention would result in a lower rupture rate compared with standard 129 rehabilitation, consistent with a superiority design. Assuming a two-sided significance level of 0.05 and 80% statistical power, a total of 194 patients per group was required to detect this difference.~~<br><br>~~131 In the present study, the primary outcome includes both surgically treated ruptures and clinically diagnosed ruptures in patients who do not undergo reoperation. This broader outcome definition is expected to result in higher observed~~	**Sample Size**<br>Authors, this section appears to be the method in which you determined the number of participants needed for your study.<br>This appears to be an unusual method based on what appears to be unpublished HAKIR data including-<br>1. Reoperation rates for FPL and FDP repairs<br>2. This HAKIR data did not include FPL ruptures determined by clinical examination only<br>3. Approximately 20% of those ruptures diagnosed by clinical exam, did not choose reoperation<br><br>**Authors**- Why did you not choose to do a Priori Analysis which is a more conventional method for determining the number of participants needed for this study?

~~rupture rates than those reported in HAKIR, which were limited to surgically treated cases.~~<br><br>~~134-138 An unpublished medical record review indicated that approximately 20% of patients with clinically confirmed tendon ruptures do not undergo reoperation. After accounting for these additional cases, the required sample size was therefore reduced, and 160 patients per group was considered sufficient. To compensate for anticipated loss to follow-up, which is expected to be substantial in this patient population, the target sample size was increased to 190 patients per group~~	
**Participant recruitment**<br>141 ~~All eligible patients diagnosed with and operated for an FPL injury, meeting the inclusion criteria and none of~~<br>~~142 the exclusion criteria, at the participating centres will be invited to participate in the study. The principal~~<br>~~143 investigator will actively monitor the recruitment progress from each centre, and the steering committee~~<br>~~144 conducts regular reviews of recruitment progress and any potential challenges. An overview of the trial~~<br>~~145 design can be found in the flowchart (Fig. 1). The enrolment, intervention schedule, and outcome measures~~<br>~~146 are illustrated in the SPIRIT diagram (Fig. 2). The first participant was recruited on 13 June 2025.~~	**Participant recruitment**<br>Authors- this section is in part a duplication of that found in lines 84-124. Please be consistent in description when presented in multiple places
**Adherence**<br>Ln 149 – 154 ~~All post-enrolment appointments are provided free of charge. During the scheduled appointments, the hand therapist ensures that the participant has filled in the PROM questionnaires in HAKIR. Participants who miss scheduled appointments will be contacted by phone or mail to reschedule. Participants who decline assessments will be asked to complete the PROMs in HAKIR. In the event of a dropout, reasons for discontinuation will be recorded.~~	**Adherence**<br>Authors- again, some of this section ln 149-159 is methodology, however your presentation is not orderly or up to standards.<br>1. What does free of charge visit have to do with method? May be a discussion point<br>2. How does the hand therapist 'ensure' that the HAKIR PROM questionnaires are completed? If this is method, write it so it is repeatable.<br>3. If the study participant (not patient) fails to complete the HAKIR PROM questionnaires, who and how is this recorded?
Ln 153- Participants who withdraw after inclusion but before undergoing the intervention will be replaced.	Ln 153 – describe how participant will be replace (repeatable method)
155 ~~Compliance~~ Adherence to ~~with~~ the EAM ~~early active motion~~ program will be assessed and promoted at follow-up appointments by the hand therapist.	Ln 155 please consider edits, describe how assessed and 'promoted' -this sentence is unclear.

Ln 156 There are no specific strategies to improve or monitor adherence to immobilisation.	Ln 156- This may be viewed as a limitation to your study. If the participant removes his/her cast prior to 4 weeks, is there no way to monitor? How do you replace that group's participant?
Ln 157-159 Participants requiring post-trial treatment will be offered our centre's standard care ~~for sequelae~~ after FPL injuries. ~~at the respective hand surgery centre.~~ Those who suffer harm by trial participation may seek compensation from the Swedish patient insurance system (LÖF).	Ln 157 -159 Consider edits
**METHODS: ASSIGNMENT OF INTERVENTIONS**<br>**Randomisation and blinding**	**METHODS: ASSIGNMENT OF INTERVENTIONS**<br>**Randomisation and blinding**
Ln 164-167 Randomisation will be conducted after the ~~obtaining~~ signed informed consent is obtained by either the dedicated surgeon, hand therapist, or research nurse. Within the HAKIR registry there is a centralised digital randomisation function, to ensure ~~within the HAKIR registry will be used, ensuring~~ allocation concealment. Participants will be randomly allocated 1:1 to either the EAM group ~~early active motion~~ or the immobilisation group with a block size of four to six. There will be no blinding.	Ln164-167<br>Authors- consider edits<br><br>Is there a software that you can cite associated with the HAKIR registry central randomization function? (repeatable method)<br><br>Provide more detail regarding no blinding, such as the intervention team (surgeon, therapist, research nurse, patient, others?) Describe to allow repeatability of your Method
**METHODS: DATA COLLECTION, MANAGEMENT AND ANALYSIS**<br>**Baseline participant and injury characteristics assessment**	**METHODS: DATA COLLECTION, MANAGEMENT AND ANALYSIS**
Ln 172-173 Baseline participant characteristics data including age, sex, injured side, tobacco use? vs. smoking, mechanism of injury ~~mechanism~~, and comorbidities (i.e., diabetes or hand osteoarthritis) are collected by the hand surgeon, hand therapist, or research nurse after inclusion or before randomization?.	Ln 172-176 Consider edit to subtitle<br>Authors- Are you tracking data regarding dominant hand involvement, as this could impact the patient reported outcome scores, and be viewed as a limitation
Ln 174 -Participants complete baseline PROMs with instructions that the assessment represent the status the last week before the injury.	Ln 174<br>Authors please be consistent in your descriptions, check this against other method sections
Ln 175 The surgeon will use the HAKIR assessment to enters ~~information about~~ the ~~level~~ zone of FPL tendon injury, associated digital nerve injury, suture technique and suture material ~~within the HAKIR assessment for all flexor tendon injuries.~~	Ln 175- Consider edit<br>What zone classification system is used for FPL tendon injury?
**Data collection**<br>Ln 179 -180 Data on PROMs, objective outcomes, and occurrence of adverse events (i.e., FPL rupture or post-operative infection) are recorded three and 12 months postoperatively in the HAKIR registry.	**Data collection**<br>Authors- please clarify,<br>1. are you collecting some of the outcome data at baseline, then at 3 and 12 months? If so please specify and correct this sentence<br>2. The HAKIR Registry is where the data is stored v. collected.

	3. Please list the data collected i.e., list each PROM, TAM or AROM, Grip strength<br>4. Adverse condition include FPL rupture noted by examination only, FPL rupture observe intraoperatively, postoperative infection
Ln 181 Participants are allowed to ~~can~~ withdraw from the trial at any time ~~point~~. In the event of participant withdrawal, data collection is discontinued, and the participant's data up to the time ~~participant's point~~ of withdrawal will be used.<br><br>Ln 183-184 In the event of a FPL rupture noted by clinical exam and/or confirmed intraoperatively, the participant will remain included in the study, ~~and~~ with the 12-month assessment ~~will be~~ remaining scheduled 12 months after the primary repair operation. ~~, regardless of any subsequent surgical intervention~~	Ln 181-184 Authors consider edits
**Data management**<br>Ln 187-188 ~~Data will be collected during the HAKIR registry's routine assessments conducted after all flexor tendon injuries three and 12 months postoperatively.~~<br><br>Ln 188-190 ~~The~~ All data collected for this study will be entered directly into the HAKIR registry and stored under the participant's assigned personal identification number. ~~All data is stored in the registry.~~ Before sending the data for analysis, the participant's data file will be de-identified by the Registry using a pseudonymized method. ~~When analyses are to be performed, the researchers will receive a pseudonymized data file from the registry~~	**Data management**<br>Ln 187-188 consider deleting as this is describes data collection not management<br>Authors- consider all edits
**Statistical plan**<br>ln193-196 Descriptive statistics will be presented as means with standard deviations (SD) for normally generated continuous variables.<br><br>Normality will be assessed using Q-Q plots. Continuous and non-parametric variables that do not follow a normal distribution but show a highly skewed distribution ~~as well as non-parametric variables~~ will be presented as median with interquartile ranges (IQR). Categorical outcomes will be presented with percentages<br><br>Ln 198-201 The primary outcome, ~~(rupture rate)~~ will be based on the intention to treat principle (ref), and analysed with the Chi-square test. The absolute risk difference and relative risk with 95% confidence intervals will be calculated. In the presence of imbalance in potential confounders (e.g., age, sex, smoking, suture technique) multivariable Poisson regression (reference)with	**Statistical plan**<br>Ln 193-196 Authors, please list the names of the variables which are the 'normally generated continuous variables'<br><br>Authors- define 'highly skewed distribution' (method repeatability)<br><br>Authors- what is a 'categorical outcomes, please provided for method completeness.<br><br>Ln 198-201<br>Please be more thorough in you explanation of statistical method for repeatability.<br>How will the 95% confidence intervals be calculated?<br>Define 'imbalance' in confounders; also consider adding hand dominance, are you

robust standard errors will be used to estimate adjusted relative risks.	considering surgeons and therapists as confounders?<br>What do you mean 'robust standard errors' to estimate adjusted relative risk?
Ln 202 Secondary outcomes, including PROMs, active range of motion, and grip and pinch strength, will be analysed using the independent-samples t-test for normally distributed data and the Mann–Whitney U test for non-normally 204 distributed and ordinal data. Results will be presented with 95% confidence intervals.	Ln 202- 204<br>Authors – Doesn't the Patient-reported outcomes i.e., qDASH for example have a validated method to score differences that you should be using? Do other PROMs have similar validated scoring systems that should be used?<br>AROM measurements can also use classification schemes that have be standardly use. This will make comparison between studies easier. TAM,<br>Grip strength is often viewed as a % of the opposite hand. This outcome can also be reviewed in regard to hand dominance involvement should you decide to include this data.
Ln 205 In addition, generalized estimating equations (GEE) (reference), adjusted for age, sex and centre will be used for analysis of differences between groups regarding secondary longitudinal outcome data with repeated measurements. A robust estimator covariances matrix will be used. The working correlation matrix is selected according to the lowest Quasi-likelihood under Independence Model Criterion (QICC) (ref) , and linear models will be used for all variables.	Ln 205-209<br>Please consider adding references and defining terms such as robust estimator…<br><br>Authors- What software system are you using for data analysis
210-213 Missing data will not be imputed in the primary analysis. For secondary outcomes, missing data will be imputed using multiple imputation with predictive mean matching, applying a donor pool size of k = 5, as part of sensitivity analyses. The imputation models will include outcome measures at relevant follow-up time points, as well as participant age and sex	Ln 210-213 Authors please explain why are you not inputting missing data for the primary outcome, rupture but are doing so for the secondary outcomes?
**Analysis**:<br>Ln 216- The trialist responsible for data analysis will remain blinded to treatment allocation. The blinded results will be presented to the writing committee for consensus on interpretation prior to unblinding.<br><br>**Interim analysis and stopping guidelines**<br>Ln 220-225 One interim analysis of the primary outcome (rupture rate) will be performed after approximately 50% of participants have been enrolled and completed the 12-month follow-up. Early stopping for efficacy will be guided by a group-sequential design using an O'Brien–Fleming alpha-spending function to preserve the overall two-sided type I error rate at 0.05. The interim efficacy boundary corresponds approximately to $p \leq 0.003$. The final analysis will use a significance	**Analysis**:<br><br>Authors- please clarify and provide<br>1. References for formulas used<br>2. Rupture rates observed on clinical exam and/or surgery?<br>3. Who composes the steering committee?<br>4. Why are you assessing risk using the secondary outcomes with only 20 participants/group v. 50% of participants enrolled to assess rupture risk?

threshold of p ≤ 0.05. Early termination for harm will be considered if there is a statistically significant difference (i.e., decrease or increase) in rupture rates is observed between the groups at the interim analysis. Decisions regarding early stopping<br>227 will be made by the steering committee after review of the blinded interim results. The interim analysis is 228 designed to detect a large treatment effect and will have approximately 80% power to detect an absolute risk difference of 13%. An interim analysis of secondary outcomes, including PROMs, range of motion and strength, will be conducted after 20 participants per group have completed 12 months of follow-up. This analysis is intended 232 to assess safety, feasibility, and early functional recovery, and will primarily be descriptive. Formal hypothesis testing of secondary outcomes at interim will not be used to guide early termination. For range of motion in the IP-joint, non-inferiority will be assessed using a predefined margin of 20 degrees. 235 Based on registry data from HAKIR, assuming a standard deviation of 24 degrees at 12 months postoperatively, a sample size of 19 participants per group provides 80% power to detect non-inferiority at a two-sided significance level of 0.05. These analyses will be considered supportive and exploratory	5. Secondary outcomes can be assessed using validated scoring methods<br>6. How was the margin of 20 degrees established?<br>7. How was the sample size of 19 determined
**Adverse events and safety**<br><br>Ln 240-248 ~~In this trial,~~ Serious adverse events ~~(SAEs)~~ are defined as events leading to hospitalization or death that are directly attributable to the intervention. For this trial, ~~no SAEs related to the interventions are expected in this trial, which compares~~ two ~~established~~ postoperative rehabilitation programs are being implemented, both designed to protect the FPL repair. ~~regimens. Other conditions related to the interventions will be considered~~ Possible adverse events may include rupture of the FPL repair or post-operative infection, which may require hospitalization. ~~(i.e., FPL rupture or post-operative infection).~~ Any tendon rupture and infection ~~All adverse events that may be associated with the intervention~~ will be recorded. If an adverse event occurs outside scheduled follow-up visits, participants will be instructed to contact their hand surgery centre. All participating centres provide on- call hand surgery services and are equipped to manage adverse events. Study participants experiencing an adverse event~~s~~ will be managed according to standard clinical practice, irrespective of trial participation. The participant and the surgeon responsible will determine whether reoperation is indicated, and will provide appropriate treatment. ~~will be provided.~~	**Adverse events and safety**<br><br>Ln 240-248 authors- consider edits

**Monitoring**	**Monitoring**
251-256 There will be no formal external monitoring of this trial. All participating centers are responsible for the oversight ~~overseeing~~ and monitoring of the ~~progress of the~~ study at their center. The Steering Committee oversight consists of the principal investigator (initials), lead on-site investigators (i.e., hand surgeons) at each centre, ~~along with~~ the Registry Holder, and the National Coordinator of HAKIR.<br>The collaborative Research Team includes hand therapists and research nurses at the participating centres, who are ~~and is~~ responsible for the practical implementation of the trial. The Steering Committee and the Research Team ~~collaborative group~~ hold monthly meetings for what purpose?.	Please consider the edits.<br>What is the agenda for the Steering Committee/Research Team monthly meetings?
All study-related information is available on the HAKIR website.Insert research link	Authors- Please provide the link to this website
**ETHICS AND DISSEMINATION**<br>**Ethical considerations**	**ETHICS AND DISSEMINATION**<br>**Ethical considerations**
Ln 261-267 This study complies with the ethical guidelines stated in the Declaration of Helsinki and has received approval from the Swedish Ethical Review Authority on 13th November 2023 (D.nr. 2023-04922-01) and 6th March 25 (D.nr. 2025-00730-02). Significant protocol amendments that may impact the conduct of the study, will only be implemented following approval from the Swedish Ethical Review Authority. The trial has been prospectively registered in ClinicalTrials.gov (NCT06836349) on 25th February 2025. Both rehabilitation programs implemented ~~regimens evaluated~~ in this study are established and routinely used in clinical practice to manage surgically repaired FPL injuries. ~~following FPL repair~~. Participants will therefore not be exposed to experimental or unproven interventions.<br>The main potential risk associated with the study is tendon rupture, which is a known complication after FPL surgical repair ~~injury~~ regardless of what rehabilitation postoperative program is used. ~~strategy~~. Additional risks include secondary thumb joint stiffness and reduced grip and pinch strength. These risks will be clearly communicated to patient before participating in the study. ~~participants prior to inclusion~~. Benefits of participation may include a potential reduced risk of tendon rupture, ~~and~~ possible improvement in ~~improved~~ functional outcomes, and more frequent ~~as well as closer~~ follow-up during the rehabilitation period.<br>All participants will receive standard postoperative care and monitoring, and any complication will be managed as outlined, according to established clinical practice.<br>Participation in the study will not affect access to	Ln 261-267 Please consider edits for clarity<br><br>Will participants be seen more frequently than others with FPL repair are traditionally seen in your centers? If yes, this may influence outcomes.

appropriate medical treatment. ~~The anticipated benefits of generating high-quality evidence to optimize rehabilitation after FPL repair are considered to outweigh the small additional risks associated with trial participation~~	
**Confidentiality**<br>287 All participant data will be handled in accordance with the General Data Protection Regulation (GDPR) and applicable Swedish data protection legislation. Study data will be collected and stored within HAKIR using each participant's personal identification number. Access to identifiable data will be restricted to authorized personnel involved in Registry administration. For research purposes, the study team will receive pseudonymized datasets in which personal identifiers have been replaced with unique study codes. The key linking study codes to personal identifiers will be stored securely within HAKIR and will not be accessible to the Research Team or Steering Committee?. All analyses will be performed on pseudonymized data, and results will be reported at an aggregated level to prevent individual identification. Participant information will be stored on secure servers with appropriate technical and organizational safeguards.	**Confidentiality**<br>Please consider edits<br>When and how will the data be destroyed?
**Patient and Public Involvement Statement**<br>Ln 299-302 Patients and the public were not involved in the design, conduct, reporting, or dissemination plans of this trial as the interventions are uncomplicated and constitute standard care following FPL ~~flexor tendon injuries~~. The study was designed by clinicians and researchers based on existing clinical evidence and HAKIR registry data.	**Patient and Public Involvement Statement**<br>Consider edits
**Communication of results, authorship and access to data**<br><br>Ln 305-314 The findings of this study will be published in peer-reviewed journals. Results will be presented through scientific posters or oral presentations at relevant conferences and meetings. Results will be shared with the participants involved through the HAKIR website. The researchers will have full access to the final trial dataset. Data generated and analysed during this study are stored within HAKIR and are subject to Swedish data protection legislation and the GDPR. Due to legal and ethical restrictions, individual-level participant data cannot be made publicly available. Access to de-identified study data may be granted to qualified researchers upon reasonable request and subject to approval by the Swedish Ethical Review Authority and the HAKIR Steering Committee. Access to the study protocol, statistical analysis plan, and analytic code will be granted after reasonable request. Requests should be directed to the corresponding author.	**Communication of results, authorship and access to data**<br><br>Consider minor edits

**Discussion:**	**Discussion**
Ln 318-323 FPL injuries remain challenging, and optimal postoperative management has not been clearly established. Although controlled EAM ~~early active mobilization~~ is widely recommended following flexor tendon repair, evidence supporting its use for postoperative management of patients with FPL repair ~~in thumb injuries~~ is limited6. Although the reason for the higher ~~reported~~ rupture rates after FPL repair compared to with finger flexor tendons is unknown, EAM after FPL repair may be contributory ~~suggest that early active mobilization may not be appropriate for FPL repair rehabilitation~~ 4-6. This registry-randomised controlled trial aims to compare the rupture rates, PROMs and clinical measurement of range of motion and strength between participants managed for ~~address this knowledge gap by comparing early active mobilisation with four~~-4-weeks of cast immobilization and those managed for 4-weeks with a thermoplastic splint and controlled EAM after thumb Zones 1-2 FPL repair.	Ln 318-323<br>Authors- consider edits and adding to references
Ln 324-329 By embedding randomisation within a national quality registry, this study combines the methodological strengths of randomised controlled trials with the efficiency and external validity of real-world data collection. This approach enables large-scale recruitment and standardized outcome assessment while minimizing additional burden on patients and healthcare providers. This registry-based design is therefore a major strength and increases the generalizability of the findings. To our knowledge this is the first RRCT in the field of hand surgery.	Ln 324-329 While the authors are quite familiar with a Registry, the reviewer and therefor perhaps the reader will not understand the underpinnings of a Registry. Authors, please include this elaboration either in your introduction (preferably) or discussion. I do not find that being the first is a strength.
Ln 330- 334 We chose the primary outcome, frequency of FPL tendon rupture within 12 weeks postoperatively because it ~~the The primary focus on tendon rupture reflects the most clinically relevant complication following FPL repair, as rupture~~ often necessitates reoperation, delays rehabilitation and is associated with risk for inferior functional outcomes2. Our secondary outcomes, including ~~In addition, the inclusion of objective functional~~ measurement of active range of motion, grip and pinch strength allow comparison to other similar studies. The use of PROMs allows participants to comment on their lived-experience. When secondary outcomes are analyzed, we may have a better idea how patient report relates to clinical measurement and frequency of FPL tendon rupture. Overall, perhaps a superior postoperative FPL repair therapy management program may identified. ~~for a comprehensive evaluation of the effects of the rehabilitation protocols. This is important given that reduced range of motion or strength may not necessarily correspond to perceived disability or dissatisfaction~~.	Ln 330-334 Please consider edits

Ln 335-344 Several limitations should be considered. The unblinded design may introduce performance and assessment bias. However, rupture rate is a clearly defined clinical endpoint and is unlikely to be substantially affected by this.	Ln 335-344 Authors what do you mean 'performance and assessment bias'? Please describe. Once defined please state why this would not substantially (define) be affected by bias. You are allowing both clinical exam and surgical observation to determine tendon rupture. If IP joint flexion is limited, could this be adhesion v. rupture?<br><br>Please consider other possible limitations pointed out by this reviewer
Secondary outcomes (range of motion and strength) may be more susceptible to bias, as treating hand therapists are unblinded assessors.	ROM and Strength can be affected by bias, but there are also other factors, non-reproducible measurement method, instrument calibration, time of day, etc. How will you determine or minimize these other factors?
Reporting bias might affect PROMs and satisfaction.	Authors describe how reporting bias might affect PROMs including patient satisfaction. How will you minimize?
~~Second, s~~urgeons will ~~are allowed to~~ use their preferred surgical repair techniques and ~~so variability in surgical techniques and~~ suture materials. These factors may influence FPL rupture risk both within and between ~~across~~ centres. ~~may influence rupture risk~~. These surgical details will be systematically recorded and considered in adjusted analyses.<br><br>~~Third, although the rehabilitation protocols have been collaboratively designed by hand surgeons and hand therapists from all participating centres to ensure consistency, adherence to rehabilitation protocols may vary between individuals and centres, which could affect treatment effects.~~	Please consider edit. How will the surgical techniques and suture materials be recorded and describe the analysis process more thoroughly in the methods section<br><br>Authors, I have not seen the reproducible description of your therapy programs for both the cast immobilization and controlled EAM plus thermoplastic splint groups. Please provide these in your method section and then describe how therapist administration may differ.
Ln345-348 The interim analyses are designed primarily to ensure participant safety and to detect large treatment effects at an early stage of data collection. Conservative stopping boundaries have been implemented to minimize the risk of false-positive findings. This approach balances ethical responsibility toward participants with the scientific integrity of the trial.	Ln 345-348<br>Please clarify what you mean by conservative stopping boundaries…and minimize false-positive
Ln 349-353 If cast immobilisation for 4-weeks after FPL repair is shown to reduce rupture rates without compromising ~~functional~~ the secondary outcomes, the results will support a change in standard postoperative management of FPL injuries. Conversely, if controlled EAM and thermoplastic splint wear for 4-weeks protects the FPL repair from rupture and the secondary outcomes are not compromised, the ~~early active mobilisation yields comparable safety and better~~	ln349-353 Authors, consider edits

~~function,~~ current practice will be supported. ~~reinforced~~. In either case, the findings of this trial are expected to provide important evidence to inform clinical practice ~~guidelines~~ and hopefully improve patient care.<br><br>Ln 354-357 ~~In conclusion, tThis registry-randomised controlled trial addresses a critical gap in evidence regarding rehabilitation after FPL repair. By~~ compares ~~two widely used treatment strategies in a pragmatic and methodologically robust framework, the trial has the potential to substantially influence future rehabilitation practices and optimize outcomes for patients with FPL injuries~~	Ln354-357 please consider the following<br><br>Five hand surgery centers submitted data to a National registry for a RCT designed to compared postoperative zone 1-2 FPL tendon rupture rate frequency, range of motion, grip and pinch strength and patient-report of satisfaction and function between participants randomized to either cast immobilization or controlled EAM and a thermoplastic splint for 4-weeks, with measurements obtained at postoperative months three and 12.
**Article Summary**:<br><br>Ln 361**Strengths and limitations of this study**<br><br>362 Randomised registry-controlled trial.<br>363 Generalizable results.<br>364 Unblinded design may bias the outcome.	**Article Summary**:<br><br>**Strengths and limitations of this study**<br><br>Authors- please separate the strengths and limitations<br>I would suggest there are other limitations than those that you have listed per this review
References	Please review the literature to include additional references and to support statements in your protocol as requested by reviewer. For example-<br>Elliot D, Southgate. New Concepts in Managing the Long Tendons of the Thumb After Primary Repair. JHT 2005, doi:10.1197/j.jht.2005.01.008<br><br>Methods of measuring thumb ROM-<br>White, % of opposite thumb IP joint motion, %TAM of opposite thumb, ASSH TAM or Buck-Gramcko methods
Figures	Review figures once protocol is revised to ensure consistent terminology is used

---

VERSION 1 - AUTHOR RESPONSE

Reviewer: 1

Mr. Ben Marson, University of Nottingham Comments to the Author:
A robust study protocol. Good luck.
Thank you!

Reviewer: 2

Comments to the Author:
Authors, your research question is important as it may add to our knowledge in the rehabilitation management of FPL repairs. Please review the attached specifics outlined by this reviewer. Please write from a rehab not surgical perspective.

We would like to sincerely thank the reviewer for the thorough and insightful review of our manuscript. We greatly appreciate the considerable time and effort devoted to providing detailed comments and constructive suggestions. We have carefully considered all comments and have implemented almost all of the proposed changes, as detailed below (answers in bold italic). We believe that these revisions have substantially improved the quality, clarity, and overall strength of the manuscript, and we are grateful for the reviewer's valuable contribution to this process.

Title Authors-Consider striking (REFLEX) from title as it seems unnecessary, striking regime and update terminology with programs or approaches, and adding Randomized Controlled Trial with a superiority design

The title is changed to: Immobilisation versus Early Active Motion Following Primary Flexor Pollicis Longus Tendon Repair: Protocol for a Registry-Based Multicentre Randomised Controlled Trial in Sweden

Most of the suggested red-marked changes from the reviewer has been made throughout the manuscript

Abstract:
Introduction Ln 7- 8

Authors- consider tendon adherence and edit and substitute FPL throughout abstract.

Flexor Pollicis Longus has been replaced by FPL throughout the manuscript after the first time.

Ln 9-13 Authors- Please revise this entire section.

1. Consider introducing the problem, significantly more FPL than FDP ruptures first, then postoperative rehab programs/regimens.

This section (abstract introduction) has been rearranged according to the suggestion (line 9-11): "Flexor pollicis longus (FPL) tendon injuries in the thumb are surgically challenging and require careful postoperative rehabilitation to avoid complications such as tendon rupture, stiffness, and impaired hand function. Rupture rates after FPL repairs are significantly higher than for finger tendons. Controlled early active motion (EAM) is the standard protocol for FPL repairs. This study investigates whether immobilisation for four weeks postoperatively can reduce rupture rates without compromising functional outcomes after FPL injuries."

2. It is your opinion that the anatomy and functional use of the thumb are factors that may correspond with higher FPL rupture rates, please remove this from the abstract. You could use this as a discussion point if supported by evidence. There are many other possible contributors to FPL rupture from surgical repair techniques, patient adherence, therapist experience, co-morbidities, mechanism of injury etc.

This section is deleted as suggested.

3. Consider why you are comparing 2 different postoperative FPL rehab regimens/program and use this as your rationale. For instance, did another HAKIR -based study suggest this possibility that EAM might be a contributor?

The Introduction of the Abstract have been revised to state the rationale for comparing immobilisation and EAM

Methods and analysis Ln 15-18 Authors consider the suggested edits

We prefer to retain the term "registry-randomised" because it describes a key methodological feature of the study design. (line 16).

1. You have not described the credentials of the hand therapists involved in this study

This is not described in the abstract but we have added information in Methods (line 104-5) regarding the qualifications of the hand therapists involved in the study. Rehabilitation is delivered by licensed occupational therapists and physiotherapists working in hand surgery units at the participating centres. All therapists have clinical experience in hand rehabilitation and the management of ffexor tendon injuries and follow a standardized rehabilitation protocol.

2. Is the cast applied at 2-5 days or intra-operatively?

Intraoperatively. This is described in the main text line and in the supplemental rehabilitation program. The text is changed to "Participants will be randomised 1:1 to either the EAM group, in which training starts two to five days postoperatively with protection by a dorsal cast and a volar removable splint, or the casted immobilisation group for four weeks."

3. Are baseline patient reported outcomes (please name these) done once the patient becomes a participant?

Baseline patient reported outcomes are now named in the abstract.

Baseline patient reported outcomes are done before the patient become a participant since all patients operated for a ffexor tendon injury fill in these forms in the registry, regardless of study participation. This is described in line 74.

Ln 19-22 Authors- consider edits

1. Please specify by name, the patient reported outcomes, i.e., QuickDASH

Owing to the word limit of the abstract, we have chosen not to list all PROM instruments individually. Full details are provided in the Methods section.

2. Active or passive ROM, of what joints?

Active ROM of IP and MCP, this is now revised (line 22-23).

3. Grip only or xxx pinch strength?

Grip and key pinch strength, now revised line 23.

4. Are these measurement of ROM and strength taken by standard methods?

Yes according the HAKIR registry manual, this is described in methods line 122.

Ln 21-22 Authors please

1. specify exactly how data collection will be done (Each therapist enters the data?) and how it will be analyzed.

PROM data is entered by the participant. Objective measurement data is entered by each hand therapist. This is described in methods; line 72-3 and 121.

2. Will there be surgical data also entered and analyzed for this RCT? Please include

Yes as described in Metods line 193-4.

Due to the word count limit, some of the requested information can not be included in the abstract but as mentioned above, all information is in the manuscript.

Key words: Please consider more appropriate key words i.e., flexor tendon, thumb, rehabilitation, immobilization, early active motion, splint

These words could not be chosen in the list in manuscriptcentral. In the title page we have the following MeSH term key words: thumb, tendon injury, rehabilitation, immobilization, postoperative complications

Introduction

Lines 32-35 Authors please consider suggested revisions. Please seek out additional more current references to support your statement of controlled EAM after finger flexor tendon repairs provides better outcomes than immobilization There are other factors that also contribute to tendon rupture, EAM is not the only factor
Changes have been made as suggested and references added.

Ln 36- consider deletion of this sentence as it implies patient adherence is responsible for tendon rupture. This is your opinion, unsupported by references and how does not support your study intent
The sentence has been deleted.

Ln 37-38-Authors please support your statements with references and consider more current references. I am not sure you intended to say patient morbidity = patient death/illness ***Morbidity has been changed to suffering (line 39) and reference a has been added.***

Ln 39- 40 Authors consider this edit

Most studies (REFERENCES) reporting on flexor tendon injury, focus on the flexor tendons of the fingers when compared to those involving the thumb.(REFERENCES)

We have changed the sentence to: Most studies on ffexor tendon injuries focus on the finger ffexor tendons, while injuries involving the Flexor Pollicis Longus (FPL) are less well studied and added references (line 41-2).

Ln 41-43 Authors consider this edit Interestingly, the rupture rate for the Flexor Pollicis Longus (FPL) has been reported to be up to 17% (REFERENCES) compared to three percent for the Flexor Digitorum Profundus (FDP). (REFERENCES)

The sentence has been revised: line 42-4.

Ln 44-47 Authors this may be a discussion point, not part of the introduction. Please remove

Removed as suggested.

Ln 48-50 Authors- Please provide all references for controlled EAM rehabilitation programs following FPL repair and support your premise that EAM following FPL repair rupture rates are high when compared to FDP EAM rehabilitation programs. One could argue that immobilization may result in less tendon excursion secondary to adhesions and also more susceptible to rupture as the tensile strength of the repair has not been progressively challenged. You state that the evidence is limited, but you have not provided that evidence for the reader to review.

Additional references describing rehabilitation following FPL repair, including Elliot & Southgate and more recent studies, have been added. The introduction has been revised to better explain the rationale for comparing immobilisation and EAM after FPL repair.

Objectives Authors, please re-consider how you have stated your objectives

1. Comparison between patients with FPL primary repairs who are immobilized for 4 weeks in a cast and patients who wear an thermoplastic splint and initiate controlled EAM motion 2-5 days post operatively. The authors' hypothesis is that those immobilized will have a statistically significant lower rupture rate, proving immobilization to be superior to EAM

2. The secondary hypothesis is that patient report of symptom, disability and strength will not differ between groups when measured at 3 and 12 months post operatively.

New wording: This study aims to compare the rupture rate after FPL primary repair between patients who are immobilised for four weeks in a cast and patients who wear a protective splint and initiate EAM two to five days postoperatively. The primary hypothesis is that the immobilised group will have a statistically lower rupture rate.

The secondary hypotheses are that patient rated report of symptoms, disability, range of motion and strength will not differ between groups when measured at three and 12 months postoperatively.

Ln 60-62 Authors please clarify if registry is inserted properly.

It is a registry-based randomised controlled trial (i.e. a randomised clinical trial embedded within a clinical registry). We have added -based throughout the manuscript.

Ln 75-79 Authors- please clarify and consider listing the sites by name, not in paragraph format

We have revised the section to improve clarity by listing the participating study sites individually. We have also clarified that all participating centres are secondary or tertiary referral hospitals in Sweden

and completed the Uppsala site (line 78-82).

Ln 80-81 Authors- who serves as the primary principal investigator and how was the hand surgeon at each centre selected to serve as co-principle investigator. (Important for research method repeatability

This is now clarified in the text: The first author (MW) serves as the principal investigator of the study. At each participating centre, a hand surgeon who participated in the study design was appointed co-principal investigator. All co-principal investigators have clinical and research expertise in ffexor tendon injuries and hand surgery. Each centre will be responsible for screening all patients referred for FPL injuries. (line 85-8).

Ln 84-84 Authors consider edits

Edited as suggested

Table 1 Authors- consider all edits

1. Please provide repeatable details regarding how a patient under 18 years is deemed sufficiently mature to understand the research implications

We have clarified the inclusion criterion for participants under 18 years of age by specifying that maturity and understanding will be assessed by the treating surgeon or investigator based on the participant's ability to understand the study information, procedures, and implications and to provide informed assent.

2. Primary repair within 14 days of injury

Changed as suggested

Authors- are patients with digital nerve injury, partial tendon injuries of other digits in the same hand included/excluded?

Yes, patients with concomitant digital nerve injuries in the thumb or other digits, as well as partial tendon injuries in other digits of the same hand, are eligible for inclusion in the study. This in now stated in the text line 92-4.

3. Define large skin defects

We have clarified the definition in the manuscript (table 1). Large skin defects are defined as skin defects requiring reconstruction with a skin graft or ffap.

4. How do you deem patient will be non compliant and unsuitable for EAM program?

We agree that "non-compliance" may be perceived as subjective. We have revised the exclusion criterion to specify observable conditions that may impair the patient's ability to safely participate in and adhere to EAM rehabilitation program, such as cognitive impairment, severe psychiatric illness, or ongoing substance abuse (table 1).

5. Please use consistent updated terminology i.e., EAM rehabilitation program

Yes.

Intervention Ln 92-94

Authors, please consider more thorough edits Authors- when is the cast applied

At operation, this is now revised, line 100.

Ln 94-96 Authors, please provide

1. more details, is the original cast worn for 4 weeks without follow-up? repeatable description of EAM program

There is information about this in the supplemental material. The cast is assessed 2-5 days postoperatively and 2 weeks postoperatively when sutures are removed.

2. how the need for additional appointments is determined. This protocol should be written to be repeatable by others.

We have clarified that additional appointments are provided when clinically indicated and are determined by the treating surgeon or hand therapist based on the patient's individual rehabilitation needs (line 107-8).

3. Please provide the online link for review

The rehabilitation programs are described in the submitted supplemental material, we do not refer to an online site. To avoid confusion, the word "online" has been deleted.

Ln 97-98 please revise for clarity

We have tried to clarify the text: In the event of an FPL rupture or other adverse event such as a postoperative infection, the need for reoperation or other intervention will be assessed by the responsible surgeon in consultation with the participant, and any indicated treatment will be performed (line 108-111).

<u>Measurement of study outcomes</u>

Ln 101-121 Authors, consider edits Authors- specify who is taking the measurements. If treating therapist, this may be a limitation Please describe all methods, not refer reviewer to HAKIR manual unless it is accessible for review

The section has been revised including most of the suggestion.

This is now revised (line 122). The HAKIR manual is available online and in English.

Detail method of scoring, qDASH please

QuickDASH is scored according to the validated scoring procedure. We have clarified this in the manuscript (line 135). If the reviewer is requesting additional methodological details regarding scoring, we would be happy to provide further clarification.

Was a Jamar pinch gauge used, if not name the brand, please.

As there is variation between centres and over time, we have rephrased to: Jamar dynamometer (or equivalent hydraulic hand dynamometer) bilateral grip and dynamometric key pinch strength (pinch gauge Jamar or equivalent) with the mean of three consecutive measurements recorded (line 125).

Please specify if NRS was verbal report or marked on a scale for all outcomes using NRS

All PROMs are collected with online follow-up questionnaires/scales via the national healthcare platform 1177 this is now stated in line 128-9.

Sample Size

Authors, this section appears to be the method in which you determined the number of participants needed for your study. This appears to be an unusual method based on what appears to be unpublished HAKIR data including

1. Reoperation rates for FPL and FDP repairs
2. This HAKIR data did not include FPL ruptures determined by clinical examination only 3. Approximately 20% of those ruptures diagnosed by clinical exam, did not choose reoperation

Authors- Why did you not choose to do a Priori Analysis which is a more conventional method for determining the number of participants needed for this study?

1+2: The sample size calculation was performed a priori before study initiation. As there are limited published data on rupture rates following FPL repair and early active mobilisation, estimates for expected event rates were based on available HAKIR registry data and supplementary medical record review data. We agree that the previous wording may have been unclear and have revised the section to clarify the rationale and assumptions underlying the power calculation (line 140-50).

Participant recruitment

Authors- this section is in part a duplication of that found in lines 84-124. Please be consistent in description when presented in multiple places

We agree that parts of the participant recruitment section overlapped with information presented earlier in the manuscript. We have therefore revised and shortened this section (line 15-7) to avoid duplication and to ensure consistency in the description of participant screening and recruitment procedures throughout the manuscript.

Adherence

Authors- again, some of this section ln 149 159 is methodology, however your presentation is not orderly or up to standards.

1. What does free of charge visit have to do with method? May be a discussion point

2. How does the hand therapist 'ensure' that the HAKIR PROM questionnaires are completed? If this is method, write it so it is repeatable.

3. If the study participant (not patient) fails to complete the HAKIR PROM questionnaires, who and how is this recorded?

Ln 153 – describe how participant will be replace (repeatable method)

Ln 155 please consider edits, describe how assessed and 'promoted' -this sentence is unclear.

Ln 156- This may be viewed as a limitation to your study. If the participant removes his/her cast prior to 4 weeks, is there no way to monitor? How do you replace that group's participant?

Ln 157 -159 Consider edits

1-3: We have revised the section (line 160-17) to clarify: how PROM completion and follow-up attendance will be recorded, how participants who miss visits will be contacted, under which circumstances participants will be replaced, how adherence to the early active motion protocol will be assessed, and that adherence to immobilisation cannot be fully monitored, which we acknowledge as a study limitation. We also removed information regarding free-of-charge visits, as this is not directly relevant to the study methodology.

METHODS: ASSIGNMENT OF INTERVENTIONS Randomisation and blinding

Ln164-167 Authors- consider edits

Is there a software that you can cite associated with the HAKIR registry central randomization function? (repeatable method)

No dedicated randomisation software is used within the HAKIR registry itself. Instead, a pre-randomised allocation list was generated using a computer program prior to study initiation. For each new participant, the HAKIR randomisation function assigns the next allocation in the pre-generated randomisation sequence. This is now clarified in line 180-3.

Provide more detail regarding no blinding, such as the intervention team (surgeon, therapist, research nurse, patient, others?) Describe to allow repeatability of your Method

We have clarified that participants, treating therapists and outcome assessors will not be blinded because of the nature of the interventions (line 184-5).

METHODS: DATA COLLECTION, MANAGEMENT AND ANALYSIS

Ln 172-176 Consider edit to subtitle Authors- Are you tracking data regarding dominant hand involvement, as this could impact the patient reported outcome scores, and be viewed as a limitation

Changes as suggested (line 190).

Ln 174 Authors please be consistent in your descriptions, check this against other method sections

We believe the reviewer is referring to PROM at baseline. This is not an outcome and therefore not mentioned in measurement of outcome. Baseline PROMs will be used as a covariate in adjustment in analysis, This in now added in (line 226) for clarity,

Ln 175- Consider edit What zone classification system is used for FPL tendon injury?

Thank you for drawing our attention to the lack of a reference for the tendon zone classification. The reference is now added (line 91).

<u>**Data collection**</u>

Authors- please clarify,

1. are you collecting some of the outcome data at baseline, then at 3 and 12 months? If so please specify and correct this sentence

A mentioned above, baseline PROM are not outcomes and therefore not mentioned in measurement of outcome. We hope the this is clearer after revision.

2. The HAKIR Registry is where the data is stored v. collected.

Data is collected and stored in the registry. This is clarified under Data management in line 208-10.

3. Please list the data collected i.e., list each PROM, TAM or AROM, Grip strength

We have chosen not to list all outcomes again here since that would repeat the text line 123-37 but we have changed the reordered the outcomes so the primary outcome comes first

4. Adverse condition include FPL rupture noted by examination only, FPL rupture observe intraoperatively, postoperative infection

The text is revised (line 266-7).

Ln 181-184 Authors consider edits

Changes as suggested (line 201-2).

Data management Ln 187-188 consider deleting as this is describes data collection not management

This information has been deleted form this section added to the data collection line 199-200.

Authors- consider all edits Statistical plan

Ln 193-196 Authors, please list the names of the variables which are the 'normally generated continuous variables'

"generated" should be "distributed". This is corrected (line 215). As it is not possible to determine in

advance which variables will be normally distributed, these cannot be specified beforehand.

Authors- define 'highly skewed distribution' (method repeatability)

We agree that the term "highly skewed distribution" was insufficiently defined. To improve clarity and reproducibility, we have revised the sentence to state that variables that are not normally distributed will be presented as median with interquartile ranges (IQR).

Authors- what is a 'categorical outcomes, please provided for method completeness.

Thank you for this comment. "Categorical outcomes" should be "categorical variables". This is now corrected (line 216).

Ln 198-201 Please be more thorough in you explanation of statistical method for repeatability. How will the 95% confidence intervals be calculated?

We have clarified that 95% confidence intervals for the absolute risk difference and relative risk will be calculated using standard methods for binomial outcomes (Line 218-9).

Define 'imbalance' in confounders; also consider adding hand dominance, are you considering surgeons and therapists as confounders?

We have revised the wording to specify that adjusted analyses will be performed if clinically relevant imbalance in baseline characteristics between groups is observed (line 219-22). There is no information about, individual surgeons or therapists so unfortunately this cannot be added as a confounder. Injury of dominant hand might be correct for, thank you for the suggestion.

What do you mean 'robust standard errors' to estimate adjusted relative risk?

We have clarified the wording by replacing "robust standard errors" with "robust variance estimation," referring to the modified Poisson regression approach commonly used for estimation of adjusted relative risks for binary outcomes (Line 221).

Ln 202- 204 Authors – Doesn't the Patient-reported outcomes i.e., qDASH for example have a validated method to score differences that you should be using?

Do other PROMs have similar validated scoring systems that should be used?

QuickDASH will be scored according the validated scoring procedure. The other PROMs do not have scoring procedures. The other PROMs are NRS-based variables and are not combined into composite scores.

AROM measurements can also use classification schemes that have be standardly use. This will make comparison between studies easier. TAM, Grip strength is often viewed as a % of the opposite hand. This outcome can also be reviewed in regard to hand dominance involvement should you decide to include this data.

We have chosen not to use TAM, as the primary focus of this study is thumb-specific joint motion

following FPL repair. Therefore, active range of motion of the MCP and IP joints will be presented separately. Objective variables will be reported in relation to the contralateral. This is now stated in line 124 and 127.

Ln 205-209 Please consider adding references and defining terms such as robust estimator…

We have made additional clarification to improve readability for readers less familiar with these statistical methods. We have therefore revised the wording to clarify that robust variance estimation is used to account for within-subject correlation in repeated measurements.

Regarding references: The statistical methods described (e.g., Chi-square test, t-test, Mann-Whitney U test, Poisson regression with robust variance estimation and GEE are well-established methods in clinical research. Our intention is for the SAP is to provide a concise description of the planned analyses rather than a methodological review of standard statistical procedures. Similarly, robust variance estimation is standard statistical terminology that is routinely reported without detailed definitions in study protocols and statistical analysis plans. To maintain clarity and brevity, we have therefore chosen to describe the methods sufficiently for reproducibility without including references or definitions for commonly used statistical techniques.

Authors- What software system are you using for data analysis Ln 210-213

As the study is ongoing and no analyses have yet been performed, the statistical software has not been finalised. The software used for the final analyses will be reported in the primary publication.

Authors please explain why are you not inputting missing data for the primary outcome, rupture but are doing so for the secondary outcomes?

We chose not to impute missing data for the primary outcome because tendon rupture is a clearly defined clinical endpoint, and primary analyses based on observed data are generally considered the most transparent and conservative approach for hard clinical outcomes. Secondary outcomes such as PROMs and objective physical variable are more prone to missing follow-up data, and multiple imputation is therefore planned as a sensitivity analysis to reduce potential bias and loss of statistical precision.

Analysis:

Authors- please clarify and provide

1. References for formulas used

Please see above in answer regarding Ln 205-209.

2. Rupture rates observed on clinical exam and/or surgery?

This is clarified earlier line 116-8.

We have corrected an error in the manuscript. The text previously stated a two-sided significance level, whereas the correct significance level is one-sided (line 260).

3. Who composes the steering committee?

We believe the reviewer refers to the Writing committee which will consist of the authors. This is clarified in line 239.

4. Why are you assessing risk using the secondary outcomes with only 20 participants/group v. 50% of participants enrolled to assess rupture risk?

The interim analysis of secondary outcomes is intended as an exploratory assessment of early functional recovery and safety rather than a formal efficacy analysis. Secondary continuous outcome (e.g., range of motion and strength, require substantially smaller sample sizes than the primary binary outcome of tendon rupture. Rupture is a relatively infrequent event and therefore requires a much larger sample size to allow meaningful between-group comparisons. No changes were made to the manuscript, as we believe the current text adequately describes the rationale for the exploratory interim analyses.

5. Secondary outcomes can be assessed using validated scoring methods

This is discussed earlier in this respons to review.

6. How was the margin of 20 degrees established?

The 20° margin was chosen based on clinical judgement regarding what would constitute a meaningful difference in thumb interphalangeal joint motion. We have clarified this in the manuscript (line 257-8).

7. How was the sample size of 19 determined

The sample size of 19 participants per group was determined by an a priori power analysis for the exploratory non-inferiority analysis of IP joint range of motion (please see line 258-60).

<u>**Adverse events and safety**</u>

Ln 240-248 authors- consider edit

Edited as suggested
Monitoring

Please consider the edits. What is the agenda for the Steering Committee/Research Team monthly meetings?

Edited as suggested.

The agenda is to ensure consistent study conduct, protocol adherence, patient recruitment, data quality, and coordination between centres (now mentioned in line 280-3).

Authors- Please provide the link to this website

Added in line 270

ETHICS AND DISSEMINATION

Ethical considerations Ln 261-267

Please consider edits for clarity Will participants be seen more frequently than others with FPL repair are traditionally seen in your centers? If yes, this may influence outcomes.

The study does not generally involve more frequent follow-up than standard care; however, because the rehabilitation programmes have been standardised within the study, some participants may have more frequent follow-up visits during the rehabilitation period compared with usual practice at their centre. This is now revised (line 300-3).

Confidentiality

Please consider edits When and how will the data be destroyed

As the study data are stored within the national quality registry HAKIR, it will not be destroyed after study completion, in accordance with Swedish legislation governing national quality registries (now stated in line 305-7).

Patient and Public Involvement Statement

Consider edits Communication of results, authorship and access to data Consider minor edits

Edited as suggested

Discussion

Ln 318-323

Authors- consider edits and adding to references

Changed as suggested

Ln 324-329 While the authors are quite familiar with a Registry, the reviewer and therefor perhaps the reader will not understand the underpinnings of a Registry. Authors, please include this elaboration either in your introduction (preferably) or discussion. I do not find that being the first is a strength.

Perhaps text did not sufficiently explain the general principles and rationale underlying registry-based randomized controlled trials (RRCTs). We have therefore revised the text to clarify that the described advantages—such as efficient recruitment, integration with routine clinical care, standardized outcome collection, and improved external validity—are established and recognized strengths of the RRCT design in general, rather than unique features of our specific study. We have also expanded the text to better explain how embedding randomization within a national quality registry supports pragmatic clinical research and enhances generalizability of the findings Line (356-63).

Ln 330-334 Please consider edits

Mostly changed as suggested

Ln 335-344 Authors what do you mean 'performance and assessment bias'? Please describe. Once defined please state why this would not substantially (define) be affected by bias. You are allowing both clinical exam and surgical observation to determine tendon rupture. If IP joint flexion is limited, could this be adhesion v. rupture?

We have revised the manuscript to clarify what we mean by performance bias and assessment bias. Performance bias refers to potential differences in postoperative management resulting from knowledge of treatment allocation, whereas detection bias refers to potential differences in outcome assessment by unblinded assessors (375-84).

Please consider other possible limitations pointed out by this reviewer

ROM and Strength can be affected by bias, but there are also other factors, non reproducible measurement method, instrument calibration, time of day, etc. How will you determine or minimize these other factors?

We agree that variability in ROM and strength measurements may arise not only from detection bias but also from measurement error and other sources of variability. In our study, these assessments were performed according to the standardized HAKIR protocol using calibrated instruments, which likely reduced measurement variability. Nevertheless, we acknowledge that some degree of random measurement error is unavoidable in clinical assessments. We have revised the manuscript accordingly line 380-2.

Authors describe how reporting bias might affect PROMs including patient satisfaction. How will you minimize? Please consider edit.

As the PROMs were collected using standardized, validated questionnaires administered according to routine follow-up procedures, opportunities to minimize reporting bias beyond these measures were limited. We have clarified this point in the manuscript and acknowledge reporting bias as a limitation that cannot be fully excluded (Line 383-4).

How will the surgical techniques and suture materials be recorded and describe the analysis process more thoroughly in the methods section

Surgical technique, core suture configuration and suture materials are recorded in HAKIR and will be included in the study database. This has been clarified in the Methods section (line 194).

Authors, I have not seen the reproducible description of your therapy programs for both the cast immobilization and controlled EAM plus thermoplastic splint groups. Please provide these in your method section and then describe how therapist administration may differ.

Detailed rehabilitation protocols for both groups have been provided as supplementary material and are referenced in the Methods section (line 106).

Rehabilitation was delivered by hand therapists at several hospitals. Although standardized protocols were used in both treatment groups, some variability in therapist-delivered care, including exercise instruction, progression and follow-up, is unavoidable in routine clinical practice. The use of predefined rehabilitation protocols was intended to minimize such variation. We have clarified this limitation in the manuscript (Line 373-5).

Ln 345-348 Please clarify what you mean by conservative stopping boundaries…and minimize false positive

We have revised the manuscript to clarify that more stringent statistical significance thresholds will be applied at interim analyses than at the final analysis line 392-3.

n349-353 Authors, consider edits

Edited as suggested

Ln354-357 please consider the following Five hand surgery centers submitted data to a National registry for a RCT designed to compared postoperative zone 1-2 FPL tendon rupture rate frequency, range of motion, grip and pinch strength and patient report of satisfaction and function between participants randomized to either cast immobilization or controlled EAM and a thermoplastic splint for 4-weeks, with measurements obtained at postoperative months three and 12.

Mostly edited as suggested

<u>Article Summary:</u>

Strengths and limitations of this study Authors- please separate the strengths and limitations I would suggest there are other limitations than those that you have listed per this review

The Article Summary has been revised to clearly separate strengths and limitations of the study.

Please review the literature to include additional references and to support statements in your protocol as requested by reviewer. For example Elliot D, Southgate. New Concepts in Managing the Long Tendons of the Thumb After Primary Repair. JHT 2005, doi:10.1197/j.jht.2005.01.008 Methods of measuring thumb ROM White, % of opposite thumb IP joint motion, %TAM of opposite thumb, ASSH TAM or Buck-Gramcko methods

Thank you, references are added and updated including this suggestion

Review figures once protocol is revised to ensure consistent terminology is use

Yes

VERSION 2 - REVIEW

**Reviewer**	**2**
**Name**	**Howell, Julianne W.**
**Affiliation**	**Self-employed Hand Therapy Consultant**
**Date**	**15-Jun-2026**
**COI**	

Thank you for your revised Protocol. I am asking that your review and consider revisions to your protocol manuscript. Investing the time now into these revisions will support the final results manuscript impact. I am still uncertain if surgeons are using the same materials and repair techniques across the centres. I look forward to your revision.

**Title- R1** Immobilisation versus Early Active Motion Following Primary Flexor Pollicis Longus Tendon Repair: Protocol 3 for a Registry-Based Multicentre Randomised Controlled Trial in Sweden	**R1 Reviewer Comments**
**Abstract**	Authors after revising manuscript, please review and revise the abstract accordingly
**Introduction**<br>Background and rationale<br>Ln 34-51<br><br>Objectives<br>Ln 54-56 This study aims to compare the rupture rate after zones 1-2 FPL primary repair between patients who are immobilised for four weeks in a cast with patients who are issued a removable protective splint and instructed in controlled ~~initiate~~ EAM two to five days postoperatively.<br><br>Ln 58-59 The secondary hypotheses are that patient ~~rated~~ report of symptoms and disability, and measurement of active range of motion and grip and pinch strength will not differ between groups when measured at three and 12 months postoperatively.<br><br>Trial design	**Introduction**<br>Background and rationale<br>Ln 34-51 Thank you for your revision<br><br>Ln 54-59 Consider revisions<br><br>Ln 62-63 Thank you for your revision
**Methods: Participants, Intervention and Outcomes**<br><br>Study setting<br>Ln 73-74 ~~for~~ Prior to surgery and again postoperatively at 3 and 12 months, patients self-reported symptoms, functional impairment, and satisfaction.<br>Ln 74-76 For ~~selected diagnoses, including~~ the diagnosis of flexor tendon injury, an extended assessment is done by the hand therapist in which ~~a hand therapist measures~~ range of motion and grip and pinch strength is done at 3 and 12 months postoperatively<br>Ln 85-87 At each participating centre, a hand surgeon who participated in the study design process-will serve as ~~was appointed~~ co-principal investigator. All surgeon co-principal investigators	**Methods: Participants, Intervention and Outcomes**<br><br>Authors- consider edits and add description of Hand Therapists involved in the study process.

have clinical and research expertise in the surgical management of flexor tendon injuries. ~~and hand surgery.~~ ADD information regarding Hand Therapist here?<br>87-88 Each centre will be responsible for screening ~~all~~ patients referred for FPL injuries	Ln 87-88 Who exactly at each center is responsible screening or examining all patients referred with FPL injury?
**Participants**<br>Ln 91-94 ~~All~~ Any patient~~s~~ referred to the ...Table 1 outlines the inclusion and exclusion criteria.<br>Patients with concomitant digital nerve injuries involving ~~in~~ the thumb or other digits, as well as partial tendon injuries in the fingers ~~other digits~~ of the same hand, are eligible for inclusion in the study<br>Table 1 Inclusion and exclusion criteria **Inclusion**<br>Complete Zone 1-2 FPL laceration.<br>Primary repair within 14 days of injury.<br>Age ≥15 years. ~~Participants~~ Patients under 18 years will ~~of age must~~ be assessed by the treating surgeon or investigator? as having sufficient maturity to understand the study information, procedures, and implications and to provide informed consent.<br>Ability to communicate in spoken and written Swedish.<br>**Exclusion**<br>Tendon injury requiring tendon grafting. Complete tendon injuries other than FPL in the same hand.<br>Associated injuries such as fractures, vascular injuries impairing circulation, skin defects requiring ~~reconstruction with~~ a skin graft or flap, or preoperative signs of infection.<br>Observable conditions, such as cognitive impairment, severe psychiatric illness, or ongoing substance abuse that may impair the patient's ability to safely participate in and adhere to EAM rehabilitation program, ~~Development of~~ Wound infection development between 2-5 days ~~at day two to five~~ postoperatively, that would contraindicate EAM rehabilitation program participation.	Ln 91-Table<br>Authors consider edits for clarity.<br>Clarify investigator?
**Intervention**<br>Ln 99-111 This trial will compare two different methods for managing patients after zones 1-2 FPL injury. These methods include either 1) ~~cast~~ immobilisation of the wrist and thumb in a dorsal? cast from operation until ~~and for a period of~~ four weeks postoperatively (experimental group) or 2) removal ~~use of a~~ the dorsal wrist- thumb dorsal cast between 2-5 days post operatively and replaced with a volar removable thermoplastic splint and implementation of controlled EAM ~~within two to five days after surgery~~ (control group).	Ln 99-111<br>Authors- consider edits

At four weeks postoperatively, participants in both groups will be given ~~instructed in~~ the same restrictions and rehabilitation program. A standardized rehabilitation protocol outlined in supplemental materials ~~Treatment~~ is delivered by ~~licensed hand~~ licensed Occupational Therapists ant Physiotherapists experienced in hand rehabilitation ~~occupational therapists or physiotherapists)~~ at each ~~the~~ participating centre. ~~according to a standardised protocol (see 106 supplemental material). All hand therapists had specific clinical experience in hand rehabilitation and flexor 107 tendon injuries.~~<br>Based on surgeon or hand therapist assessment, analgesics and additional appointments according to the individual patient needs ~~of the patient~~ are permitted when clinically indicated. ~~based on assessment by the treating surgeon or hand therapist.~~<br>In the event of an FPL rupture or other adverse event such as a postoperative infection, the need for reoperation or other intervention will be assessed by the responsible surgeon in consultation with the participant to determine the need for ~~if and~~ further ~~any~~ indicated treatment. ~~will be performed.~~<br><br>**Measurement of ~~study~~ outcomes**<br>Ln 114 -118 The primary outcome variable is the frequency of FPL tendon rupture over a 12-months period. If a rupture is suspected based on clinical assessment (i.e., no active thumb flexion at interphalangeal (IP) joint), the treating therapist will notify the responsible hand surgeon. If surgery is pursued, observation intraoperatively will be used to confirm the tendon rupture. If the participant declines reoperation, the diagnosis will be based on the clinical assessment.<br>Ln 119 Secondary outcome variables include range of motion, grip and pinch performance measurements, and ~~objective function and~~ patient-reported outcomes measurements (PROMs). ~~at three and 12 months postoperatively.~~<br><br>Ln 121-122 The treating therapist will measure and enter the ~~objective function is measured and~~ data ~~entered in~~ as described in the HAKIR English manual[13] as follows: ~~by a hand therapist, that may be the treating 122 therapist, according to the HAKIR measurement manual that is available online and in English13 including:~~	Authors – consider adding Table to outline(see my comments in supplementary materials section)<br><br>Authors- consider edits and suggestions Please note that AROM and grip/pinch measures are indirect measures of function and are not objective, especially when measured by the treating therapist. I would suggest 'objective and function' be deleted throughout the manuscript.<br><br>Authors- for repeatability of 2ndary variables-

Ln 123-125<br>• Goniometric measurement of active range of motion of thumb metacarpophalangeal (MCP) and IP-joints. Active range of motion of the MCP and IP joints will be presented as absolute values and relative to the contralateral side<br><br>Ln 126-129<br>• Jamar dynamometer (or equivalent hydraulic hand dynamometer) bilateral grip strength and dynamometric key pinch strength (pinch gauge Jamar or equivalent) with the mean of three consecutive measurements recorded. Grip and key pinch strength will be presented as absolute values and relative to the contralateral side<br><br>Ln 130-140<br>PROMs are collected (When?)with online follow-up questionnaires via the national healthcare platform 117714 and include:<br><br>• HAKIR symptom survey (HQ-8)17, an ~~patient-reported~~ 11-point numerical rating scale (NRS) 0-100 with a higher score indicating worse symptoms rating. Symptoms surveyed include- pain on loading, pain during motion, pain with no load, pain at rest, weakness, stiffness, cold sensitivity, numbness and activities of daily life (ADL) experienced over the previous week.<br><br>• Quick disability of the arm, shoulder, and hand (QuickDASH)17. QuickDASH is an 11-item score ~~self~~ assessment of symptoms and disability in the upper limb, ~~s~~ rating from 0 (no disability) to 100 (most severe 137 disability~~) according to~~ with a validated scoring procedure and CMID defined (reference)<br><br>• HAKIR 11-point NRS[13] ~~scale~~ of ~~patient rated~~ overall satisfaction with the surgical outcome using a scale from 0-100~~. is assessed with a self-administered 11-point NRS (0-100)13.~~<br><br>• Five-point Likert scale of overall satisfaction18 with rehabilitation.	Ln 123-125 please describe method of goniometric measurement, for example composite MCPJ + IPJ flexion/extension, active/passive motion, what is the absolute value (% of contralateral thumb?)<br>Describe goniometer and placement for measurement<br>When is ROM taken?<br><br>Ln 126-129 Authors- was the reliability of these instruments at each center tested?<br>Please revise this section for clarity<br>What is the absolute value, i.e., percentage of contralateral hand?<br>When are strength performance measures taken?<br><br>Authors- I do not understand an 11-point scale when you describe a rating scale between 0-100? Clarify, please.<br>Is this survey validated and reliable with the CMID defined?<br><br>Authors- not clear on the 11-point NRS what are these points?<br>Is this NRS validated and is CMID defined?<br><br>Authors – please expand Likert description including 0= ? 5=?<br>Validated? CMID defined?<br><br>Authors- for this section refer to Figure 2?
Sample size<br>Ln 143-152 The sample size calculation was performed a priori for the primary	Sample Size<br>Ln 143-152<br>Please consider naming your groups to describe intervention

outcome of tendon rupture. Based on previous HAKIR data[8] (reference?), the expected rupture rate was estimated at 10% ~~in~~ for the ~~control~~ immobilized group and 3% ~~8~~ ~~in~~ for the Splint/EAM ~~intervention~~ group, corresponding to an absolute risk reduction of seven percentage points.<br><br>The primary hypothesis was that 4 weeks ~~the~~ postoperative immobilization ~~intervention~~ would result in a lower rupture rate compared with 4 weeks of postoperative splinting/EAM using ~~standard rehabilitation, consistent with~~ a superiority design. Assuming a two-sided significance level of 0.05 and 80% statistical power, a total of 194 patients per group was required.<br><br>Because this ~~present~~ study ~~also~~ includes both clinically diagnosed FPL ruptures and intra-operatively confirmed FPL ruptures ~~not treated surgically~~, event rates are expected to be higher than those reported in HAKIR (ref) that ~~which only~~ captures only surgically confirmed ~~treated~~ tendon rupture frequency. Based on supplementary medical record review data, the final target sample size was set at 190 participants per group to allow for anticipated loss to follow up	Please clarify<br><br>Is there a better word?<br><br>What is supplementary medical record review? How did you use this to establish loss to fu?
Participant recruitment<br>Ln 155 -159 Eligible patients at participating centres will be invited to participate in the study. The principal investigator will monitor recruitment progress at all centres, and the steering committee will regularly review recruitment and any potential challenges. An overview of the trial design is presented in Figure 1, and the enrolment, intervention schedule, and outcome measures are illustrated in Figure 2. The first participant was recruited on 13 June 2025.<br><br>Adherence<br>Ln 162-175 Participants will attend routine follow-up visits according to the rehabilitation protocol at each participating centre. During scheduled follow-up visits, the hand therapist or research staff will remind participants to complete the PROM questionnaires in HAKIR and assist with technical issues if needed. Completion of PROM questionnaires and attendance at follow-up visits will be recorded in HAKIR. Participants who miss scheduled appointments will be contacted by phone, electronic message, or mail and offered a new appointment. Reasons for withdrawal or discontinuation will be documented when	Participant recruitment<br>Ln 155 -159<br><br>How is this done?<br><br>Adherence<br>Ln 162-175<br>Consider edits

available. Participants who withdraw before randomisation or before initiation of the allocated rehabilitation protocol will be replaced. Adherence to the Splint/EAM group ~~program~~ will be assessed by the treating hand therapist during follow-up visits based on participant self-report and clinical evaluation. Participants allocated to the immobilisation group will be instructed to maintain cast immobilisation continuously for four weeks. Adherence to immobilisation cannot be fully monitored. Participants requiring post-trial treatment will be offered the centre's standard care for FPL injuries. Those who suffer harm by trial participation may seek compensation from the Swedish patient insurance system (LÖF).	
METHODS: ASSIGNMENT OF INTERVENTIONS<br>Randomisation and blinding<br><br>Ln 180-197 Randomisation will be conducted after signed informed consent is obtained by either the dedicated surgeon, hand therapist, or research nurse. Within the HAKIR registry there is a centralised digital randomisation function to ensure allocation concealment. A pre-randomised allocation list was generated using a computer program prior to study initiation. For each new participant, the HAKIR randomisation function assigns the next allocation in the pre-generated randomisation sequence. Participants will be randomly allocated 1:1 to either the splint/EAM group or the immobilisation group with a block size of four to six. There will be no blinding of participants, treating therapists or outcome assessors because of the nature of the interventions.<br><br>METHODS: DATA COLLECTION, MANAGEMENT AND ANALYSIS<br>Baseline participant and injury assessment<br><br>Ln 192 -197 Baseline data including age, sex, injured side, smoking, mechanism of injury, injury of dominant hand, comorbidities (i.e., diabetes or hand osteoarthritis) are collected by the hand surgeon, hand therapist, or research nurse after inclusion. Participants complete the baseline PROMs with instructions to report on their ~~that the assessment represent the~~ status using the ~~last~~ week before the injury as a reference point. The surgeon enters information about the zone of FPL ~~level of~~ tendon injury, associated digital nerve injury, surgical technique and core suture	METHODS: ASSIGNMENT OF INTERVENTIONS<br><br>Ln 180-197<br>Authors- is the randomization assignment sequence for each centre or all centres sequentially?<br><br>What computer program?<br><br>METHODS: DATA COLLECTION, MANAGEMENT AND ANALYSIS<br><br>Ln 192 -197<br>Authors- smoking or tobacco use?<br>Authors- consider edits

configuration within the flexor tendon injury HAKIR assessment. ~~for all flexor tendon injuries~~<br><br>Outcome data collection<br>Ln 199-207 Data includes the number of FPL tendon ruptures, ~~on occurrence of FPL rupture,~~ PROMs, MCPJ and IPJ thumb ROM, grip and pinch strength of injured and contralateral hands, ~~objective outcomes~~, and ~~other~~ adverse events (i.e., postoperative infection) are collected as required by ~~during~~ the HAKIR registry~~'s~~ as well as 3 and ~~routine assessments conducted after all flexor tendon injuries three and~~ 12 months postoperatively. ~~in the HAKIR registry.~~<br><br>Participants are allowed to withdraw from the trial at any time. In the event of participant withdrawal, data collection is discontinued and the participant's data up to the time of withdrawal will be used and analyzed. In the event of an FPL rupture noted either by clinical exam and/or confirmed intraoperatively, the participant will remain included in the study and assessed as planned at 3 and ~~with the~~ 12-months? ~~assessment remaining scheduled 12 months~~ after the primary repair. ~~Operation~~	Outcome data collection<br>Ln 199-207<br>Authors- consider edits and clarify when each data is collect on each variable.<br>Perhaps refer to Fig 2
Data management<br>Ln 210-212 All data will be entered directly into the HAKIR registry and stored under the participant's personal identification number. When analyses are to be performed, the researchers will receive a pseudonymised data file from the registry.	Data management<br>Authors- consider edits
Statistical plan<br>Ln 215 -224 Descriptive statistics will be presented as means with standard deviations (SD) for normally distributed continuous variables. Normality will be assessed using Q-Q plots. Non-parametric and continuous variables that are not normally distributed will be presented as median with interquartile ranges (IQR). Categorical variables will be presented as numbers with percentages. The primary outcome (rupture rate) will be analysed according to the intention to treat principle using the Chi-square test. The absolute risk difference and relative risk with corresponding 95% confidence intervals calculated using standard methods for binomial outcomes. ~~, will be reported.~~ If clinically relevant imbalance in baseline characteristics (e.g., age, sex, smoking, injury of dominant hand, suture technique and core suture configuration) is	Statistical plan<br>Ln 215 -224<br>Authors, consider edits and highlight

observed between groups, multivariable Poisson regression with robust variance estimation will be used to estimate adjusted relative risks.<br><br>Ln 225-237 Secondary outcomes, including PROMs, range of motion, and grip and pinch strength, will be analysed using the independent-samples t-test for normally distributed data and the Mann–Whitney U test for non-normally distributed and ordinal data. Results will be presented with 95% confidence intervals. In addition, generalized estimating equations (GEE), adjusted for age, sex, baseline PROM and centre will be used for analysis of repeated secondary outcome measurements. Robust variance estimation will be applied to account for within-subject correlations in repeated measurements. The working correlation structure will be selected according to the lowest Quasi-likelihood under Independence Model Criterion 232 (QICC), and linear models will be used for all variables. Missing data for the primary outcome will not be imputed, as tendon rupture represents a clearly defined clinical endpoint and the primary analysis will be based on observed data only. For secondary outcomes, missing data will be imputed using multiple imputation with predictive mean matching, applying a donor pool 236 size of k = 5, as part of sensitivity analyses. The imputation models will include outcome measures at relevant follow-up time points, as well as participant age and sex.<br>Analysis<br>Ln 240-241 The trialist responsible for data analysis will remain blinded to treatment allocation. The blinded results will be presented to the writing committee (i.e. the authors) for consensus on interpretation prior to unblinding.<br><br>Interim analysis and stopping guidelines<br>Ln 244 -253 One interim analysis of the primary outcome (rupture rate) will be performed after approximately 50% of the participants have been enrolled and completed the 12-month follow-up.	 <br>Ln 225-237<br>Authors- consider edits, and highlighted area needs clarity.<br><br>Authors- will data for each centre be analyzed and/ or taken as a whole?

Early stopping for efficacy will be guided by a group-sequential design using an O'Brien–Fleming alpha-spending function to preserve the overall two-sided type I error rate at 0.05. The interim efficacy boundary corresponds approximately to $p \leq 0.003$. The final analysis will use a significance threshold of $p \leq 0.05$. Early termination for harm will be considered if there is a statistically significant difference (i.e., decrease or increase) in rupture rates is observed between the groups at the interim analysis. Decisions regarding early stopping will be made by the steering committee after review of the blinded interim results. The interim analysis is designed to detect a large treatment effect and will have approximately 80% power to detect an absolute risk difference of 13%.	
Ln 254-263 An interim analysis of secondary outcomes, including PROMs, range of motion and grip and pinch strength, will be conducted after 20 participants per group have completed 12 months of follow-up. This analysis is intended to assess safety, feasibility, and measures of range of motion and strength ~~early functional recovery~~ and will primarily be descriptive. Formal hypothesis testing of secondary outcomes at interim will not be used to guide early termination. For active range of motion in the IP-joint, non-inferiority will be assessed using a predefined margin of 20°. The 20° margin was chosen based on clinical judgement regarding what would constitute a meaningful difference in thumb IP-joint motion. Based on registry data from HAKIR, assuming a standard deviation of 24 degrees at 12 months postoperatively, a sample size of 19 participants per group provides 80% power to detect non-inferiority at a one-sided significance level of 0.05. These analyses will be considered supportive and exploratory.	Ln 254-263<br>Authors consider edits
Adverse events and safety<br>Ln 266-274 Serious adverse events are defined as events leading to hospitalisation or death that are directly attributable to the intervention. For this trial two rehabilitation programs are implemented, both designed to protect the FPL repair. Possible adverse events may include FPL rupture noted by examination ~~only~~ or intraoperative~~ly~~ confirmation, and post-operative infection which may require hospitalisation. Any tendon rupture or infection will be recorded. If an adverse event occurs outside scheduled follow-up visits, participants will be instructed to contact their hand surgery centre. All participating centres provide on-call hand surgery	Adverse events and safety<br>Ln 266-274<br>Author consider edit

services and are equipped to manage adverse events. Study participants experiencing an adverse events will be managed according to standard clinical practice, irrespective of trial participation. The participant and the surgeon responsible will determine whether reoperation is indicated and the centre will provide appropriate treatment.<br><br>Monitoring<br>Ln 277-285 There will be no formal external monitoring of this trial. All participating centres are responsible for the oversight and monitoring the study at their centre. The Steering Committee oversight consists of the principal investigator (MW), lead on-site investigators (i.e., hand surgeons) at each centre, the registry holder and the National Coordinator of HAKIR. The collaborative Research Team includes hand therapists and research nurses at the participating centres who are responsible for the practical implementation of the trial. The Steering committee and the Research Team will meet regularly as required, with meeting held ~~between~~ monthly and every third month~~s~~, depending on needs, to ensure consistent study conduct, protocol adherence, patient recruitment, data quality, and coordination between centres. All study-related information is available on the HAKIR website (HAKIR | Handkirurgiskt kvalitetsregister).	Monitoring<br>Ln 277-285<br>Consider edit
ETHICS AND DISSEMINATION<br>Ethical considerations<br><br>Ln 290-307 This study complies with the ethical guidelines stated in the Declaration of Helsinki and has received approval from the Swedish Ethical Review Authority on 13th November 2023 (D.nr. 2023-04922-01) and 6th March 25 (D.nr. 2025-00730-02). Significant protocol amendments that may impact the conduct of the study, will only be implemented following approval from the Swedish Ethical Review Authority. The trial has been prospectively registered in ClinicalTrials.gov (NCT06836349) on 25th February 2025. 295 Both rehabilitation programs implemented in this study are established and routinely used in clinical practice to manage surgically repaired FPL injuries. Participants will therefore not be exposed to experimental or unproven interventions. The main potential risk associated with the study is tendon rupture, which is a known complication after FPL surgical repair regardless of what rehabilitation postoperative program is used. Additional risks include secondary thumb joint stiffness and	ETHICS AND DISSEMINATION<br>Ethical considerations<br><br>Ln 290-307<br>Authors, consider minor edits

reduced grip and pinch strength. These risks will be clearly communicated to patients before participating in the study. Benefits of participation may include a potential reduced risk of tendon rupture, possible improvement in functional outcomes. The study does not generally involve more frequent follow-up than standard care; however, because the rehabilitation programmes have been standardised within the study, some participants may require ~~have~~ more frequent follow-up visits during the rehabilitation period compared with usual practice. ~~at their centre.~~ All participants will receive standard postoperative care and monitoring, and any complication will be managed as outlined, according to established clinical practice. Participation in the study will not affect access to appropriate medical treatment .	
Information of the trial and approval of the participant	Information of the trial and approval of the participant
Ln 310-316 All participants will be given written and verbal ~~oral~~ information before signing the informed consent to participate ~~is obtained~~. Only patients who ~~All participants enrolled in the study will~~ provide written informed consent (Supplemental Material) will be entered into the study. ~~themselves.~~ Informed consent will be obtained ~~from participants~~ by a surgeon, research nurse, or hand 313 therapist, immediately after surgery, or at the first outpatient clinic appointment ~~at the outpatient clinic~~, two to five days after surgery. , when rehabilitation will start. At that visit, the study's purpose, methods, potential benefits, and risks will be explained. ~~Participants~~ Patients can decline or withdraw their consent at any time during the study. Informed consent will be recorded ~~both~~ in both the medical record and ~~in~~ the HAKIR registry.	Ln 310-316<br>Authors- consider edits<br>Note until informed consent is signed, the patient is a patient not a participant<br><br>Do the cast immobilized patients also return to clinic 2-5 days after surgery?
Confidentiality<br>Ln 319-329 All participant data will be handled in accordance with the General Data Protection Regulation (GDPR) and applicable Swedish data protection legislation. Study data will be collected and stored within HAKIR using each participant's personal identification number. As the study data are stored within the national quality registry HAKIR, it will not be destroyed after study completion, in accordance with Swedish legislation governing national quality registries. Access to identifiable data will be restricted to authorised personnel involved in Registry administration. For research purposes, the study team will receive	Confidentiality<br>Ln 319-329<br>Authors, good.

pseudonymised datasets in which personal identifiers have been replaced with unique study codes. The key linking study codes to personal identifiers will be stored securely within HAKIR and will not be accessible to the Steering Committee or Research team. All analyses will be performed on pseudonymised data, and results will be reported at an aggregated level to prevent individual identification. Participant information will be stored on secure servers with appropriate technical and organisational safeguards.	
Patient and Public Involvement Statement<br><br>Ln 332-334 Patients and the public were not involved in the design, conduct, reporting, or dissemination plans of this trial as the interventions are uncomplicated and constitute standard care following FPL injuries. The study was designed by clinicians and researchers based on existing clinical evidence and HAKIR registry data.	Patient and Public Involvement Statement<br>Ln 332-334- good
Communication of results, authorship and access to data<br><br>Ln 337-345 The findings of this study will be published in peer-reviewed journals. Results will be presented through scientific posters or oral presentations at relevant conferences and meeting. Results will be shared with the participants involved through the HAKIR website. The researchers will have full access to the final trial dataset. Data generated and analysed during this study are stored within HAKIR and are subject to Swedish data protection legislation and the GDPR. Due to legal and ethical restrictions, individual-level participant data cannot be made publicly available. Access to de-identified study data may be granted to qualified researchers upon reasonable request and subject to approval by the Swedish Ethical Review Authority and the HAKIR Steering Committee. Access to the study protocol, statistical analysis plan, and analytic code will be granted after reasonable request. Requests should be directed to the corresponding author.	Communication of results, authorship and access to data<br><br>Ln 337-345- good
DISCUSSION<br>Ln 349-356<br>~~FPL injuries remain challenging, and optimal postoperative management has not been clearly established~~. ~~Although~~ Use of controlled EAM combined with a strong suture repair is ~~widely~~ recommended following ~~flexor tendon~~ FPL repair Sirotakova & Elliot (1999) and Sirotakova &	DISCUSSION<br>Authors – please note, I believe that your introduction and discussion needs to include this information.<br>1. The direct evidence regarding surgical and therapy management of FPL zones 1-2 repairs is limited. The most recent

Elliot (2004). There are no studies comparing immobilization to use of EAM after a multistrand core suture FPL repair ~~evidence supporting its use following~~ ~~for postoperative management of patients with FPL repair is limited~~2,6. ~~Although the reason for the higher reported rupture rates after FPL repair compared to finger flexor tendons is unknown~~, ~~EAM after FPL repair 353 may be contributory 7-9.~~<br><br>This Registry-Based Multicentre Randomised Controlled Trial (RRCT~~) aims to~~ compares the tendon repair rupture rate, PROMs and ~~clinical measurement of~~ range of motion, and grip and pinch strength measurements of patients with mulistrand core FPL zones 1-2 repairs ~~participants~~ managed ~~for four~~ 4-weeks with either cast immobilisation or a combination splint/~~those managed for four weeks with~~ controlled EAM.	literature Sirotakova & Elliot (1999) and Sirotakova & Elliot (2004) suggest as suture strength increases the rupture rate when use with EAM decreases. There are, however, no direct comparisons between immobilization and EAM following repair of FPL zones 1-2 injuries.<br>2. EAM in general following tendon repair, reduces adhesion formation and stiffness<br>3. Multistrand core suture repairs are intended to support EAM v. immobilization<br>In summary-The current literature (cohort studies and extrapolation from finger flexor tendon evidence favors use of controlled EAM.<br>These references (2, 6) are outdated, not used correctly, and should be revised based on the current evidence (Sirotakova & Elliot (1999) and Sirotakova & Elliot (2004)
Ln 357-365 This study benefits by embedding randomisation into ~~within~~ the structure of a national quality registry, ~~study applies~~ the methodological framework of a RRCT lessens selection bias, allows comparison between different intervention groups and organization to prospective data collection, yet may lack the controlled environment of a RCT. ~~In general, RRCTs combine the internal validity of randomised controlled trials with the efficiency, inclusiveness and external validity of prospective registry-data collection~~. Because patient inclusion, follow-up, and outcome assessment are integrated into routine clinical practice, this facilitates large-scale recruitment, ~~standardised data collection,~~ and long-term follow-up while reducing additional burden on patients and healthcare providers. Registry-based designs are therefore particularly valuable for pragmatic clinical research and for improving the generalisability of study findings to real-world practice. To our knowledge this is the first RRCT in the field of hand surgery. We chose the primary outcome, frequency of FPL tendon rupture postoperatively because it often necessitates reoperation, delays rehabilitation and is associated with risk for inferior functional outcome3,5.	Ln 357-365<br>Authors-<br>-consider edits<br>-consider updated references. Many of your references are greater than 20 years old.<br>- review your discussion points with the purpose of eliminating repetition.<br>- revise the last summary paragraph
Ln 367-395 Our secondary outcomes, including ~~functional~~ measurements of active range of motion,	

grip and pinch strength allow comparison to other similar studies. The use of PROMs allows participants to self-rate their ~~experienced~~ disability and satisfaction ~~experience~~. When secondary outcomes are analysed, we may have a better idea how patient reported disability relates to clinical measurements and rupture rate frequency. ~~of FPL tendon rupture.~~ As a result of this study, ~~Overall,~~ perhaps a superior postoperative ~~FPL repair~~ therapy management program may be identified.

Several limitations should be considered. First, the unblinded design may introduce performance bias, as treating surgeons and therapists were aware of treatment allocation, which ~~and this~~ may ~~have~~ influence~~d~~ postoperative management~~. Also,~~ As different hand therapists across several centres delivered the rehabilitation there may be ~~was delivered by different hand therapists across several hospitals~~, some variation in ~~therapist-delivered~~ care and program progression, is ~~unavoidable in clinical practice~~ even ~~if~~ when implementing standardised rehabilitation protocols. ~~were used.~~ Assessment bias is also a possibility as ~~because outcome~~ the assessors were also the primary therapist and not blinded.

However, the primary outcome, tendon rupture, is based on predefined clinical criteria and, when applicable, surgical confirmation will be done, which may reduce the risk of ~~substantial~~ misclassification. Nevertheless, we cannot exclude the idea that some clinically diagnosed i.e., not surgically confirmed ruptures may have represented impaired thumb IP joint flexion due to tendon adhesions.

Secondary outcomes (range of motion and strength) are more susceptible to detection bias, because these measurements ~~assessments~~ are ~~performed~~ administered, not independently but by the ~~treating~~ hand therapist treating the participant. ~~In~~ Additionally, some measurement variability cannot be ~~is un~~avoided despite the use of standardised assessment protocols and calibrated instruments. Patient-reported outcome measures ~~and~~ including satisfaction may be influenced by reporting bias, as participants were aware of their treatment allocation. Although PROMs were collected using standardised validated questionnaires, reporting bias cannot be excluded.

Likewise, surgeons will use their preferred surgical repair techniques and suture materials. These

factors may influence FPL rupture risk both within and between centres. These surgical details will be systematically recorded and considered in adjusted analyses. ~~Third,~~<br><br>Although the rehabilitation protocols have been ~~collaboratively~~ designed collaboratively by hand surgeons and hand therapists from all participating centres ~~to ensure~~ for consistency, adherence to rehabilitation protocols may vary between individuals and centres, which could affect outcomes. ~~treatment effects.~~<br><br>The interim analyses are designed primarily to ensure participant safety and to detect large treatment effects at an early stage of data collection. More stringent statistical significance thresholds will be applied at interim analyses than at the final analysis. This reduces the probability of incorrectly concluding that a treatment effect exists based on random variation while maintaining the overall type I error rate of the trial.<br><br>Ln 396-406 If cast immobilisation for four weeks after FPL repair is shown to reduce rupture rates without compromising the secondary outcomes, the results will support a change in the current Registry ~~standard~~ postoperative management. ~~of FPL injuries~~. Conversely, if ~~wearing a~~ splint/EAM ~~wear for 4-weeks~~ protects the FPL repair from rupture ~~and the~~ without compromise to the secondary outcomes ~~are not compromised~~, the current Registry practice will be supported.<br><br>In either case, the findings of this trial are expected to provide important evidence to inform clinical practice and hopefully improve patient care.<br>In conclusion, five hand surgery centres submit data to a National registry for a RRCT designed to compared the frequency of tendon repair rupture after ~~postoperative~~ zones 1-2 FPL tendon repair. ~~rupture rate frequency~~, analyze 3-12 month range of motion, grip and pinch strength and patient-reported outcomes ~~satisfaction and function~~ between those randomized to either 4-weeks of cast immobilisation ~~four weeks~~ or splint/ controlled EAM. ~~, with measurements obtained at postoperative months three and 12~~	
ARTICLE SUMMARY<br>Strengths and limitations of this study<br><br>Ln 410-418<br><br>Strengths:	ARTICLE SUMMARY<br>Strengths and limitations of this study<br><br>Ln 410-418

• The unique use of a registry-based to execute a randomised controlled trial comparing cast immobilisation and controlled early active motion following primary zones 1-2 FPL repair.<br>• Embedding the trial within the Swedish national hand surgery registry (HAKIR) enables efficient recruitment , reduced burden on surgeons, therapists and other medical staff by use of standardised outcome collection. The multicentre design enhances the generalisability of the findings to real-world practice.<br>• Review of the Registry's current practice with the intent of improving quality of care for patients with this diagnosis<br><br>Limitations:<br>• Blinding of participants and outcome assessors is not feasible because of the nature of the interventions.<br>• Adherence to rehabilitation protocols cannot be fully monitored as patient-centered adjustments to therapy may be required.<br>• Surgical materials and repair techniques vary between surgeons who repair zone 1-2 FPL injuries	Authors- this list is limited, please consider the edits and add strength/limits as you revise your protocol
REFERENCES	Authors, continue to update/revise your references, please
Legends to figures and Figures	Okay for now, may need revision with manuscript
Registry-Randomized Study of Rehabilitation Regimen After Flexor Tendon Injury of the Thumb<br><br>The surgical procedure is the same for all patients…	Registry-Randomized Study of Rehabilitation Regimen After Flexor Tendon Injury of the Thumb<br><br>Authors, please confirm that all surgical procedures are the same for this study as stated in this document
Supplemental material<br>Rehabilitation Protocols	Supplemental material<br>Rehabilitation Protocols<br><br>Authors- KEY POINT your protocol manuscript lacks necessary information regarding intervention rehab program. This is the crux of your study, please consider making a table for your protocol that lists-<br>1. Position of wrist/thumb in cast and splint<br>2. Pictures of the cast and splint used

	3. Timelines in table format would be helpful. For example, within the protocol manuscript, you did not state that the immobilization group returned at 2-weeks for suture removal/cast inspection<br>4. EAM exercises descriptions, frequency and duration(Table) or pictures should be included<br>5. 4-week, 6-week, 8-week, 10-week and 12-week exercises for both groups in Table format with details.<br>6. When is the protective splint/cast weaning or discharged?<br>7. Are you saying that rehab appointments were at 2-4-12 weeks only if doing well (3 visits?)

VERSION 2 - AUTHOR RESPONSE

Response to Reviewer 2

We thank the reviewer again for the detailed and constructive review of our protocol manuscript and its supplemental rehabilitation material. We have revised both documents throughout, with all changes tracked, and respond to each comment point by point below.

Abstract

Reviewer comment	Author response
Authors – after revising the manuscript, please review and revise the abstract accordingly.	We reviewed the abstract following all revisions to the manuscript. No further modifications were considered necessary.

Introduction

Objectives (Ln 54–59)

Reviewer comment	Author response
Consider revisions.	Suggested revisions have been made. We have reviewed the Objectives for clarity and consistency with the group naming used throughout the revised manuscript (see our response to the Sample size comments below, where we have standardised group

	names to "splint/EAM group" and "immobilisation group" throughout).

Methods: Participants, Interventions and Outcomes

Study setting (Ln 73–88)

Reviewer comment	Author response
ADD information regarding Hand Therapist here? / Authors– consider edits and add description of Hand Therapists involved in the study process.	We have revised this section and added a description of the role of hand therapists in the study.
Ln 87–88: Who exactly at each center is responsible for screening or examining all patients referred with FPL injury?	We have clarified that screening of all patients referred for FPL injuries at each centre is the responsibility of the co-principal investigator, in collaboration with delegated study staff.

Participants (Ln 91–94) and Table 1

Reviewer comment	Author response
Authors consider edits for clarity.	Suggested edits have been made.
Table 1: Authors consider edits for clarity. Clarify investigator?	We have changed "investigator" to "co-principal investigator" in Table 1 for clarity, consistent with the terminology used elsewhere in the manuscript.

Intervention (Ln 99–111)

Reviewer comment	Author response
Authors consider edits	Most suggested edits have been made. However, we need to clarify that patients in the EAM group continues to wear the dorsal splint, and therefore we have not changed the text regarding this.
"...in a dorsal? cast..." – clarify cast type.	We have added "dorsal" to the description of the immobilisation cast, consistent with the cast type already specified for the splint/EAM group and with the supplemental material.
Authors– consider adding Table to outline (see my comments in supplementary materials section).	We have restructured the supplemental material into four tables (initial cast/splint position; immobilisation-group timeline; splint/EAM-group timeline; and the shared 4–12-week protocol) and added a cross-reference to these tables in this section of the main manuscript. Please see our detailed response under "Supplemental material" below.

You did not state that the immobilization group returned at 2 weeks for suture removal/cast inspection (see Supplemental material comments) / Do the cast immobilized patients also return to clinic 2–5 days after surgery?	We have now added a sentence to this section stating that both groups attend in-person visits at two to five days (cast or splint check), two weeks (suture removal), four weeks (transition to the shared rehabilitation protocol) and 12 weeks postoperatively. We have also clarified this in the consent section (see below): both groups attend the first outpatient visit at two to five days; immobilisation-group patients have their cast checked, while splint/EAM-group patients begin training at this visit.
Are you saying that rehab appointments were at 2-4-12 weeks only if doing well (3 visits)?	We have added an explanatory note in the supplemental material clarifying that in-person follow-up visits are compulsory at 2, 4 and 12 weeks for both groups, while the 6-, 8- and 10-week progression points are self-directed by the patient according to the standardised protocol and do not require an additional clinic visit unless clinically indicated.

Measurement of study outcomes (Ln 114–140)

Reviewer comment	Author response
Authors- consider edits and suggestions	Most edits have been made.
Please note that AROM and grip/pinch measures are indirect measures of function and are not objective... I would suggest 'objective and function' be deleted throughout the manuscript.	We have removed "objective function" and "objective outcomes" terminology throughout the manuscript and replaced it with specific descriptions (range of motion, grip and pinch strength) in the Measurement of outcomes and Outcome data collection sections.
Ln 123–125: please describe method of goniometric measurement (e.g., composite MCPJ+IPJ flexion/extension, active/passive motion); what is the absolute value (% of contralateral thumb?); describe goniometer and placement; when is ROM taken?	We have expanded this description to specify that active range of motion is recorded as the extension and flexion of the MCP and IP joints, measured with a standard finger goniometer placed over the dorsal aspect of each joint, at the three- and 12-month assessments, and presented in degrees and a percentage of the contralateral side. To avoid ambiguity, the term "absolute values" has been removed from the manuscript.
Ln 126–129: was the reliability of these instruments at each center tested? Please revise for clarity. What is the absolute value (% of contralateral hand)? When are strength measures taken?	We have added a statement that all dynamometers are calibrated according to the HAKIR measurement manual to ensure comparability across centres and specified that grip and key pinch strength are measured at the three- and 12-month assessments and presented in kilograms and as a percentage of the contralateral side.
I do not understand an 11-point scale when you describe a rating scale between 0–100? Clarify. / not clear on the 11-point NRS what are these points?	We have clarified that these are numerical rating scales with 11 discrete response options spanning 0 to 100 in increments of 10 (i.e., 0, 10, 20 … 100), with a higher score indicating worse symptoms (HQ-8) or greater satisfaction (HAKIR satisfaction NRS), as applicable.

Is this survey validated and reliable with the CMID defined? (HQ-8, QuickDASH)	We have stated that the HQ-8 symptom survey is validated for the Swedish hand surgery population (reference 16), but that a minimal clinically important difference (MCID) has not yet been established for this instrument. For QuickDASH, we have added a citation reporting an MCID of approximately 8 to 15 points in populations with upper-limb musculoskeletal disorders (new reference, Galardini et al. 2024).
Authors – please expand Likert description including 0=? 5=? Validated? CMID defined?	We have expanded the description of the five-point Likert satisfaction scale to specify its anchors (1 = very dissatisfied to 5 = very satisfied) and noted that an MCID has not been established for this instrument.<br>We have also clarified outcomes section by specifying that HQ-8 and QuickDASH are collected preoperatively and at 3 and 12 months postoperatively, while satisfaction with the surgical outcome and rehabilitation is assessed only at the 12-month follow-up.
Authors– for this section refer to Figure 2?	We have added a reference to Figure 2.

Sample size (Ln 143–152)

**Reviewer comment**	**Author response**
Please consider naming your groups to describe intervention. Please clarify.	We have replaced "control group", "intervention group" and "experimental group" with consistent descriptive names – "splint/EAM group" and "immobilisation group" – throughout the Sample size section and the rest of the manuscript (Intervention, Randomisation and Discussion sections).
Is there a better word? ("assuming")	We have changed "assuming" to "based on".
What is supplementary medical record review? How did you use this to establish loss to follow-up?	We have revised the text and added a reference for the expected higher combined event rate that results from including clinically diagnosed (non-surgically treated) ruptures.

Participant recruitment (Ln 155–159)

**Reviewer comment**	**Author response**
How is this done? The principal investigator will ***monitor*** *recruitment*<br>*progress at all centres, and the steering*<br>*committee will regularly* ***review*** *recruitment*	We have clarified that recruitment will be monitored through regular review of HAKIR recruitment reports. Recruitment progress and any challenges related to enrolment will also be discussed at steering committee meetings.

Adherence (Ln 162–175)

Reviewer comment	Author response
Consider edits.	Suggested edits have been made

Methods: Assignment of Interventions

Randomisation and blinding (Ln 180–197)

Reviewer comment	Author response
Is the randomization assignment sequence for each centre or all centres sequentially? What computer program?	We have clarified that the pre-randomised allocation sequence is stratified by participating centre, with each participating centre having its own allocation list.<br>The randomization module was developed within the .NET application framework by a programmer employed by the registry. We did not include the software because it is a technical implementation detail that is not relevant to the interpretation or reproducibility of the study.

Methods: Data Collection, Management and Analysis

Baseline participant and injury assessment (Ln 192–197)

Reviewer comment	Author response
Smoking or tobacco use?	We have changed "tobacco use" to "smoking" in the baseline assessment, consistent with the terminology used in the HAKIR registry.
Authors– consider edits.	Suggested edits were made

Outcome data collection (Ln 199–207)

Reviewer comment	Author response
Authors– consider edits and clarify when each data is collected on each variable. Perhaps refer to Fig 2.	We have made suggested edits, removed the "objective outcomes" terminology (replaced with range of motion, grip and pinch strength) and added a cross-reference to Figure 2.

Data management

Reviewer comment	Author response
Authors– consider edits.	Edit has been made.

Statistical plan (Ln 215–237)

Reviewer comment	Author response
Authors, consider edits and highlight.	Suggested edits have been made.
Authors– consider edits, and highlighted area needs clarity. Will data for each centre be analyzed and/or taken as a whole?	We have clarified that centre is included as a fixed covariate in the GEE model to account for between-centre variation, while data from all participating centres are analysed together within a single combined model rather than separately by centre.

Analysis, Interim Analysis, Adverse Events, Monitoring

Reviewer comment	Author response
Interim analysis of secondary outcomes (Ln 254–263): Authors consider edits.	Suggested edit have been made.
Adverse events and safety (Ln 266–274): Author consider edit.	Suggested edits have been made.
Monitoring (Ln 277–285): Consider edit.	Suggested edits have been made.

Ethics and Dissemination

Ethical considerations (Ln 290–307)

Reviewer comment	Author response
Authors, consider minor edits.	Suggested edits have been made.

Information of the trial and approval of the participant (Ln 310–316)

Reviewer comment	Author response
Authors– consider edits. Note until informed consent is signed, the patient is a patient not a participant.	Suggested edits are made. We have revised this section to consistently distinguish "patients" (before consent) from "participants" (after consent is provided), throughout.
Do the cast immobilized patients also return to clinic 2–5 days after surgery?	Yes. We have made this explicit: both groups attend the first outpatient visit at two to five days after surgery. Participants allocated to the splint/EAM group begin training at this visit, while participants allocated to the immobilisation group have their cast checked and continue immobilisation.

Confidentiality / Patient and Public Involvement / Communication of results

Reviewer comment	Author response

Confidentiality (Ln 319–329): Authors, good. / Patient and Public Involvement Statement: good. / Communication of results, authorship and access to data: good.	Thank you.

Discussion

We adopted many of the reviewer's suggested language revisions. Following these changes, we undertook a comprehensive revision of the Discussion section to improve clarity, flow and structure. Several sections were reorganised and expanded, including the rationale for outcome selection, strengths and limitations of the RRCT design, sources of potential bias, and the anticipated clinical implications of the study.

**Reviewer comment**	**Author response**
Authors – please note, I believe that your introduction and discussion needs to include this information: (1) direct evidence regarding surgical and therapy management of FPL zones 1-2 repairs is limited; the most recent literature (Sirotakova & Elliot 1999, 2004) suggests that as suture strength increases, the rupture rate with EAM decreases, though there are no direct comparisons between immobilisation and EAM for FPL repair; (2) EAM in general following tendon repair reduces adhesion formation and stiffness; (3) multistrand core suture repairs are intended to support EAM versus immobilisation. In summary, current literature (cohort studies and extrapolation from finger flexor tendon evidence) favours EAM. These references (2, 6) are outdated, not used correctly, and should be revised based on the current evidence (Sirotakova & Elliot 1999, 2004).	We have incorporated all three points into the Discussion. (citing Sirotakova & Elliot 1999 and 2004, new references 5 and 6), while noting that no studies have directly compared immobilisation with EAM specifically after FPL repair; we also added a sentence noting that current literature, drawn chiefly from cohort studies of FPL repair and extrapolation from finger flexor tendon evidence, favours EAM. We have restructured the opening paragraph to state explicitly that direct comparative evidence is limited, to cite the Sirotakova & Elliot findings, to note that EAM more broadly reduces adhesion formation and joint stiffness. We address the possible impact of core sutures developed specifically to support safe EAM. Rreferences 2 (Chesney et al.) and 6 (Starr et al.) have been replaced with Sirotakova & Elliot (1999, 2004) at both citation locations (Background and Discussion), as requested.
Ln 357–365: Authors– consider edits and updated references. Many of your references are greater than 20 years old. Review your discussion points with the purpose of eliminating repetition. Revise the last summary paragraph.	We have added the Sirotakova & Elliot references requested by the reviewer (1999, 2004) and a 2024 reference for QuickDASH MCID. We have retained a small number of older citations (e.g., Verdan 1960, Strickland 1980) as foundational, historically significant references for flexor tendon repair. We have rewritten the concluding paragraph paragraph.
Ln 396-406 If cast immobilisation for four weeks after FPL repair is shown to reduce rupture rates without compromising the secondary outcomes, the results will support a change in the current Registry standard postoperative management. of FPL injuries. Conversely, if wearing a splint/EAM wear for 4-weeks protects the FPL	We would like to clarify that there is currently no standard treatment within HAKIR. Management strategies vary between centres, which was one of the reasons for conducting the present study.

repair from rupture and the without compromise to the secondary outcomes are not compromised, the current Registry practice will be supported.	

Article Summary

Strengths and limitations of this study (Ln 410–418)

Reviewer comment	Author response
Authors– this list is limited, please consider the edits and add strength/limits as you revise your protocol.	We have added two further strengths (a prespecified, registry-data-informed sample size calculation; standardised, collaboratively developed rehabilitation protocols and validated PROMs across all centres) and two further limitations (lack of standardisation in surgical repair technique and suture material between surgeons and centres; susceptibility of unblinded, therapist-assessed secondary outcomes to detection and reporting bias).

References / Legends to figures and Figures

Reviewer comment	Author response
Authors, continue to update/revise your references, please.	Addressed via the additions described above (Discussion section response).
Legends to figures and Figures: Okay for now, may need revision with manuscript.	Noted; we will finalise the figures alongside any further manuscript revisions requested by the editor.

Supplemental Material – Rehabilitation Protocols

Reviewer comment	Author response
"Registry-Randomized Study of Rehabilitation Regimen After Flexor Tendon Injury of the Thumb / The surgical procedure is the same for all patients..." – Authors, please confirm that all surgical procedures are the same for this study as stated in this document.	This statement does not appear in the current version of the supplemental material. For clarity, we confirm that surgical repair technique and suture material are not standardised across surgeons or centres in this trial (as stated in the Discussion limitations); only the postoperative rehabilitation protocol is standardised across centres, which is the subject of the supplemental material.
Authors– KEY POINT your protocol manuscript lacks necessary information regarding intervention rehab program. This is the crux of your study, please consider making a table for your protocol that lists: (1) position of wrist/thumb in cast and splint; (2) pictures of	We have substantially revised the supplementary material by restructuring it into four tables as suggested by the reviewer, describing the initial postoperative position, the rehabilitation protocols for each treatment group during the first 4 weeks, and the shared rehabilitation programme from 4 to 12 weeks. We have clarified clinic visits, suture removal, cast/splint

the cast and splint used; (3) timelines in table format (e.g., the 2-week suture removal/cast inspection visit was not stated); (4) EAM exercise descriptions, frequency and duration in table or picture format; (5) 4-, 6-, 8-, 10-and 12-week exercises for both groups in table format; (6) when is the protective splint/cast weaned or discharged; (7) clarify whether rehab appointments were only at 2-4-12 weeks (3 visits).	discontinuation, exercise descriptions, permitted loading, and follow-up schedules. We have not included photographs of the cast and splint, as these are standard postoperative devices and we believe the written descriptions are sufficient. However, should the Editor consider photographs necessary, we will make every effort to provide them.

We thank the reviewer again for the thorough and constructive feedback, which we believe has substantially strengthened both the protocol manuscript and the supplemental material.

VERSION 3 - REVIEW

**Reviewer**	**2**
**Name**	**Howell, Julianne W.**
**Affiliation**	**Self-employed Hand Therapy Consultant**
**Date**	**08-Jul-2026**
**COI**	

Authors, thank you for the time and this revision. I have suggested another review to allow you to clearly state the study's purpose, clearly define terminology and to write so the method can be replicated. There are many more specific details in the attached document. This revision was vastly better than the first. Thank you.

**bmjopen-2026-118506.R2**	**Reviewer Comments**
**Title**-Immobilisation versus Early Active Motion Following Primary Flexor Pollicis Longus Tendon Zones I-II Repair: Protocol for a Registry-Based Multicentre Randomised Controlled Trial in Sweden	Authors- consider edit
**Abstract**<br>Ln 7 Introduction Flexor pollicis longus (FPL) tendon injuries ~~in the thumb~~ are surgically challenging and require careful 8 postoperative rehabilitation to avoid complications such as tendon rupture, stiffness, and impaired hand 9 function. ~~Rupture rates after FPL repairs are substantially higher than those reported for finger flexor 10 tendons. Although controlled early active motion (EAM) is widely used following FPL repairs, rehabilitation 11 protocols developed for finger flexor tendons may not be directly transferable to the thumb because of 12~~	**Abstract**<br><br>Ln 10-14 Authors, after revision of your manuscript, please re-write the abstract purpose with supporting background information.<br><br>37

~~differences in anatomy, biomechanics and functional demands. This study investigates whether 13 immobilisation for four weeks postoperatively can reduce rupture rates without compromising functional 14 outcomes after FPL injuries~~.<br><br>Ln 15 Methods and analysis<br>~~This registry-based randomised controlled unblinded superiority trial will be conducted across five academic 17 hand surgery centres. Patients aged 15 years or older with a complete FPL injury in zone 1 or 2 will be 18 included. Participants will be randomised 1:1 to either the EAM group, in which training starts two to five 19 days postoperatively with protection by a dorsal cast and a volar removable splint, or the casted 20 immobilisation group for four weeks. After four weeks, the same rehabilitation protocol will be introduced for 21 both groups. The primary outcome is the frequency of tendon rupture over 12 months. The secondary 22 outcomes include patient-reported outcome measurements and patient-reported satisfaction, active range of 23 motion of metacarpophalangeal and interphalangeal joints, grip and key pinch strength at three and 12 24 months postoperatively. All data is collected within the Swedish National Quality Registry for Hand Surgery, 25 HAKIR.~~<br>Ln 26 Ethics and dissemination<br>The trial was approved by the Swedish Ethical Review Authority (2025-00730-02). All participants are asked 28 to provide written informed consent. The results will be disseminated as published articles in peer-reviewed 29 journals	 <br><br><br><br><br>Ln 15-25 Authors, after revision of the manuscript, please revise the abstract method section and include analysis which you have omitted here
**Article Summary**<br>Ln 36 This registry-based randomised controlled trial compares ~~cast~~ immobilisation with controlled early 37 active motion following primary zone 1–2 flexor pollicis longus tendon repair.<br><br>Ln 38 Embedding the trial within the Swedish National Registry for Hand Surgery (HAKIR) enables ~~efficient~~ 39 multicentre recruitment, ~~standardised~~ methodologically consistent collection of outcome measurements ~~collection~~ and enhances generalisability to ~~routine~~ 40 clinical practice.<br><br>Ln 41 For this study, we used previous registry data to support our prespecified sample size	**Article Summary**<br>Ln 36-48 Authors consider edits and suggestions.<br><br>'Efficient' is your opinion only- strike<br>Your use of outcome tools and methods of measurements did not always follow the standards established for hand surgery and therapy research data.<br>Please avoid use of term 'standardised'.<br><br>Ln 41 Consider separating these characteristics.

calculations. ~~based on registry data, together with standardised 42~~<br><br>Ln 42 The same rehabilitation splint fabrication specifics and exercise protocols and ~~validated~~ patient-reported outcome measures were used by therapists in each of the centres. ~~strengthens the 43 methodological quality of the study~~	Ln 42- Authors, I would argue that these factors in an of themselves do not strengthen your method quality per se but do demonstrate that you strived for consistency.
Ln 44-45 Blinding of participants and outcome assessors is not feasible because of the nature of the 45 interventions.	Ln 44- please don't mix unrelated factors
Ln 45, ~~and adherence to rehabilitation protocols cannot be fully monitored~~.	Ln 45 Just as in the clinical setting, adherence to rehabilitation protocols, splint and cast wear cannot be fully monitored. Is this a strength or weakness or similar to clinical practice?
Ln 46 • In this Swedish system, surgical repair techniques and suture materials are not standardised across centres.	Ln 46 Is this a strength or weakness or similar to clinical practice?
Ln 47 , and some 47 secondary outcomes are assessed by treating, unblinded hand therapists, introducing a potential 48 risk of detection bias	Ln 47-48 please consider combining this thought with lines 44-45.<br>Others
**Introduction**<br>Ln 54- Controlled early active motion (EAM) is typically initiated a few days after surgery, and for repairs of the finger flexor tendons has ~~as EAM has 55~~ produced better range of motion compared to cast immobilisation. ~~following flexor tendon repair involving the 56 fingers~~1,2.	**Introduction**<br>Ln 54- Consider edits
Ln 59 Most flexor tendon repair studies ~~on flexor tendon injuries~~ focus on the tendons of the fingers compared to studies after ~~flexor tendons, while injuries involving~~ the Flexor 60 Pollicis Longus (FPL) repair. ~~are less well studied~~5,6. This disparity is of interest to us as ~~Interestingly,~~ the rupture rate for FPL repairs has been reported 61 to be as high as 17% compared with approximately three percent for the Flexor Digitorum Profundus (FDP) 62 repair7-10 .	Ln 59-please consider edits
Ln 63 Although EAM is widely implemented following finger and thumb flexor tendon repair, some have suggested that EAM after ~~following~~ FPL repair3,9,11, ~~it~~ may be associated with higher re-rupture 64 rates7-10.	Ln 63-64 Consider edit.<br>Elliott and Southgate (2005) looked at repair techniques v. rupture rate after EAM. These authors discussed that zone of injury (FPL), tendon retraction after injury and different methods of repair may be contributors to rupture rate. The 17% rupture rate was noted by the authors when they used their oldest surgical repair technique and material (group 1) ; eventually after modification of

	suture technique and materials (group 4) rupture rate was 0% . Please review this 2005 review of studies by done by Elliot/Southgate on their patients overtime to be sure your introduction is representative of their findings.
Ln 64 and immobilisation could hypothetically represent a safer alternative.	Ln 64 Authors- is this your opinion? If so state that, if not, you will need to cite references that support your statement. OR provide strong rationale based on findings of others (Elliot and Southgate, primarily)
Ln 65 Concerns have been raised that 65 rehabilitation protocols developed for finger flexor tendons may not be directly transferable to FPL repairs 66 because of differences in tendon excursion, biomechanics and functional demands of the thumb12.	Ln 65 Did Elliot and Southgate actually say this? (2005)<br>"This increased tension from FPL muscle shortening may help to explain the higher rupture rate in the repaired FPL tendon as compared to that of finger flexor" p141<br><br>Vascularity issues associated with- Zone 2 FPL higher rupture rate than Zone 1 per Elliot and Southgate p 142<br><br>See Table 1 in this 2005 article that shows distribution of RR recorded by various authors between 1989 and 2004. Note that RR have decreased since Kasashima et al. (2002)[28]<br><br>Authors- KEY POINT Please review and revise your rationale for this study.
Ln 66 ~~Conversely,~~ 67 Reports from 2004 to date have emphasized use of EAM~~-~~(cite -Authors from Table 1 in Elliot and Southgate 2005 see copy at end of this review) ~~has been advocated to~~ reduce tendon tethering, ~~adhesion formation~~ and loss of interphalangeal (IP) joint 68 motion following FPL repair~~12~~. Rupture rates have decrease when these authors used either immobilization, dynamic splinting, Duran-Houser passive motion, or and EAM following FPL repairs since 2002 (references Table 1). To date,there have been no	Ln67 Authors consider revision suggestions<br>Authors, please cite primary sources not only reference 12<br>Ln 67 Authors- consider this train of thought ....<br><br>Ln 68 delete<br>40

rehabilitation program comparison studies to identify an optimal program.<br><br>~~Ln 68 Therefore, the optimal postoperative rehabilitation strategy after FPL repair 69 remains unclear. No studies have directly compared immobilisation with EAM specifically after FPL repair~~<br><br>**Objectives**<br>Ln 72-78 This study aims to compare the rupture rate after zones 1-2 FPL primary repair between patients who are 73 immobilised for four weeks in a cast with patients who at 2-5 days postoperativelyare issued a removable protective splint and 74 instructed in controlled EAM exercises. ~~two to five days postoperatively.~~<br>Ln 74 The primary hypothesis is that the immobilised 75 group will have a statistically significantly lower rupture rate.<br>Ln 76 The secondary hypotheses are that patient-reported outcome measurements of (specified each) ~~symptoms and disability~~ and measurements of active 77 range of motion and grip and pinch strength will not differ between the immobilized or EAM groups ~~when measured~~ at three and 12 78 months postoperatively<br>**Trial Design**	Ln 72-please consider edits<br><br>Ln 74-<br>Consider edit<br><br>Ln 76 – Consider edits<br><br>**Trail Design**- okay
**METHODS: PARTICIPANTS, INTERVENTIONS AND OUTCOMES**<br>Study setting-<br>Ln 93- Normally participating centres 94 perform ~~participating in~~ an extended follow-up assessment programme for patients at 3 and 12 months postoperatively. Anytime a flexor tendon has been repaired assessment by ~~perform~~ hand therapists is also done and includes measurement assessments at three and 12 months 95 postoperatively in patients with flexor tendon repairs including measurements of range of motion and grip 96 strength.<br><br>Intervention<br>Ln 122- This trial will compare two different methods for managing patients after zones 1-2 FPL repair~~injury~~. All patient will be fitted with a postoperative dorsal wrist-thumb cast. Those randomized to the immobilization group will wear the cast time. Those randomized to the EAM group will have the cast discontinued between 2-5 days post-surgery and replaced with a volar removable wrist-thumb thermoplastic splint and controlled EAM exercises. ~~These 123 methods include either 1) immobilisation of the wrist and thumb in a~~	Ln 93- consider edits<br><br>Intervention<br><br>Ln 122 consider edits<br><br>41

~~dorsal cast from operation until four 124 weeks postoperatively (immobilisation group) or 2) wrist-thumb dorsal cast and a volar removable 125 thermoplastic splint and implementation of controlled EAM (splint/EAM) within two to five days after surgery 126 (splint/EAM group).~~	
Ln 129 Both groups will attend in-person visits at two to five days postoperatively for a cast check ~~and~~ or fabrication of a splint; 130 ~~check~~, week-2 postoperatively for ~~two weeks (~~suture removal~~)~~, at week-4 postoperative to ~~four weeks (~~transition to the shared rehabilitation program, and at week-12 131 ~~weeks~~ postoperatively for final assessment?? ~~further appointments are scheduled when clinically indicated~~.	Ln 129 consider edits<br><br>Authors- KEY POINT please add the supplemental material to your described intervention section. It is missing
**Measurement of outcomes**	**Measurement of outcomes**<br>**Authors, you have not discussed evaluation of primary outcome – tendon rupture, you will need to define what is considered a rupture and what is not (define) I do not endorse calling a tendon ruptured if active tendon glide does not progress, it needs to be visualized to compare your RR with other studies.**
Ln 143 Secondary outcomes variables include active range of motion, grip and pinch performance measurements, 144 and patient-reported outcome measures (PROMs).	Ln 143- Authors, KEY POINT to enable comparison your outcomes with others, please consider converting your AROM measures to White and/or Buck-Gramcko range of motion scoring
Ln 147 Goniometric bilateral measurement of active range of motion of the thumb metacarpophalangeal (MCP) and 148 IP joints. Active extension and flexion of the MCP and IP joints will be measured at the three- and 12-month 149 assessments using a standard finger goniometer placed over the dorsal aspect of each joint. Active range of 150 motion of the MCP and IP joints will be presented in degrees and as a percentage of the contralateral side.	Ln 147- Authors, please detail exactly how AROM is taken isolated MCP and IP joint or composite MCP and IP joint motion.<br>Authors-presenting AROM as a percentage of the opposite thumb is good, but please add the White and/or Buck-Gramcko method, too. Please add to your method
Ln 151 ~~Bilateral~~ Jamar dynamometers ~~(~~or equivalent hydraulic hand dynamometer and Jamar or equivalent pinch gauges will be used. ~~)~~ ~~grip strength~~ and ~~dynamometric 152 key pinch strength (pinch gauge Jamar or equivalent)~~. Each grip and pinch measurement will represent the mean of three consecutive measurements taken	Ln 151- Authors, consider edits<br><br>42

in a standard position (reference standard here). ~~will be 153 recorded.~~<br><br>Ln 153 All dynamometers are calibrated according to the HAKIR measurement manual to ensure 154 comparability of measurements across centres. Grip and key pinch strength are measured at the three- and 155 12-month assessments and reported in kilograms and as a percentage of the contralateral side.<br><br>Ln 156 PROMs will be ~~are~~ collected ...<br><br>Ln 158 HAKIR symptom survey (HQ-8)17, is a ~~patient-reported~~ numerical rating scale (NRS) with 11 response options 159 ranging from 0 to 100 in increments of 10, with a higher score indicating worse symptoms.<br><br>Ln 159 HQ-8 is 160 administered three times- preoperatively, ~~and at~~ three and 12 months postoperatively.<br><br>Ln 162 This instrument has been validated for the 163 Swedish hand surgery population.17~~; a~~ The minimal clinically important difference (MCID) has not yet been 164 established for this instrument.<br><br>Ln 165 The Quick disability of the arm, shoulder, and hand (QuickDASH)18 is ~~an~~ validated 11-item self-assessment of 166 symptoms and disability in the upper limb, scored from 0 (no disability) to 100 (most severe disability). ~~with a validated procedure.~~ QuickDASH is administered three times- preoperatively, ~~and at~~ three and 12 months postoperatively. 168 A MCID is considered to be a difference of ~~of approximately~~ 8 to 15 points. 19 ~~has been reported for QuickDASH in populations with upper-limb 169 musculoskeletal disorders19~~<br><br>Ln 170 Patient~~-rated overall~~ satisfaction with the surgical outcome is assessed in this HAKIR NRS 11-item survey at 12 months 171 postoperatively ~~with a self- administered NRS with 11~~ Patients response options range from 0 to 100 in increments 172 of 10, with a higher score indicating greater satisfaction14.<br><br>Ln 174 A five-point Likert scale of ~~overall~~ satisfaction20 with rehabilitation will be assessed at12 months postoperatively. The 5-	Ln 153- Authors how often are the dynamometers calibrated? Prior to study or on regular basis- please mention.<br>I would suggest that for your study calibration is most importantly done to also ensure similar repeatability (reliability)at the same v. between centers to compare each patient's measurement to themself?<br><br>Ln 156- 174 consider edits<br><br>43

point scale ranges ~~and 175 ranging~~ from 1 (very dissatisfied) to 5 (very satisfied). A MCID has not been established for this instrument<br><br>**Adherence**<br>Ln 213 Adherence to the ~~Splint/~~EAM group will be assessed by the treating hand therapist during follow-up visits 214 based on participant self-report and clinical evaluation.<br>Ln 214 Participants allocated to the immobilisation group will 215 be instructed to maintain cast immobilisation continuously for four weeks.<br>Adherence to immobilisation 216 cannot be fully monitored.	Authors- KEY POINT Likert scale data (symptoms and satisfaction) can be analyzed without having a MCID established, please be sure to address in data analysis section if you have not already.<br><br>**Adherence**<br>Ln 213- consider edits<br><br>Ln 214- Authors- are they instructed to return if cast issues?<br>Are both groups instructed on hand use limitations?<br>Authors- why are you directing this statement toward the immobilization group only, wouldn't this be the same for the EAM group, too?
**METHODS: ASSIGNMENT OF INTERVENTIONS**<br>Ln 226 Participants will be randomly allocated 1:1 to either 229 the immobilisation group or the ~~splint/~~EAM group,	**METHODS: ASSIGNMENT OF INTERVENTIONS**<br>Ln 226 consider edit from here forward
**METHODS: DATA COLLECTION, MANAGEMENT AND ANALYSIS**<br><br>Ln 247 Participants are allowed to withdraw from the trial at any time. In the event of participant withdrawal, data 248 collection is discontinued and the participant's data up to the time of withdrawal will be used and analysed.	**METHODS: DATA COLLECTION, MANAGEMENT AND ANALYSIS**<br><br>Ln 247 – Authors- in other words, if patient self-discharges at any point 1-week postoperatively to 11-weeks postoperatively the data will be analyzed?
**DISCUSSION**<br>Ln 397 ~~Although controlled EAM is widely recommended after FPL repair, the evidence supporting this 398 recommendation is based primarily on observational studies and extrapolation from digital flexor tendon 399 repairs5,6~~<br>~~Ln 399 Furthermore, reported rupture rates following FPL repair are higher than those reported after 400 repair of the digital flexor tendons7-10.~~	**DISCUSSION**<br>Ln 397 Authors, I would question this statement. Please refer to **Table 1** below from Elliot /Southgate 2005 and make an adjustment to your statement.<br><br>Ln 399- Authors, I would disagree with your generalized statement, as with stronger repairs, FPL rupture rates have decreased<br>(**See Table 1**) since 2002 and are perhaps less than that of finger flexor tendon RRs.

~~Ln 400 In general, EAM following flexor tendon repair is reported to reduce adhesion formation and joint stiffness compared with immobilisation, and multistrand core suture techniques 402 have been developed to withstand the loads generated during EAM5,6~~.	Consider deletion of these points and get to your objective which is the fact that there have been no comparison studies!<br><br>Ln 400 -402- Please clarify and revise this sentence or consider deleting.
Ln 402 ~~However, no~~ The authors of this study plan to ~~has directly 403 compared~~ the implementation of immobilisation to ~~and~~ EAM intervention following FPL zones I-II repair7-9~~. Consequently, the relative~~ The intent of this study is to better understand the effects of these ~~404 rehabilitation strategies~~ interventions on tendon rupture rate, active motion, grip and pinch performance, and ~~function, al recovery,~~ and patient-reported outcomes. ~~remain 405 uncertain~~. ~~This RRCT was designed to address knowledge. gap by comparing four weeks of cast 406 immobilisation with splint/EAM following primary zone 1-2 FPL repair.~~	Ln 402- 406 Consider edits
Ln 407 The primary outcome is tendon repair rupture, while active MCP and IP joint active motion, ~~range of motion~~, grip and pinch 408 strength, and PROMs are assessed as secondary outcomes at preoperatively, and at three and 12 months postoperatively. 409 Tendon rupture was selected as the primary outcome because it frequently necessitates reoperation, delays 410 rehabilitation and is associated with less desirable ~~an increased risk of inferior functional~~ outcomes3,4. The secondary 411 outcomes were chosen to capture both clinical and patient-reported aspects of recovery and to facilitate 412 comparison with previous studies ~~of~~ following flexor tendon zones I-II repair.	Ln 407<br>Authors consider all edits<br><br>Ln 410- outcomes could also be satisfaction, pain, - not specifically poor function<br>Ln 412-Authors – KEY POINT this is why your range of motion data needs to be converted to White's and Buck-Gramcko scoring i.e., to compare with other studies.
~~Ln 413 PROMs provide insight into participants' perceived 413 disability and satisfaction. Together, these outcomes will provide a comprehensive assessment of both 414 structural and functional recovery following FPL repair.~~	Ln 413-14 repetitive, please strike
Ln 415 ~~A major strength of this study is the integration of randomisation within a national quality registry. The 416 methodological framework of a RRCT reduces selection bias, enables comparison of treatment strategies 417 and combines the methodological advantages of~~	Ln 415 -423<br>Authors, consider deleting and revision<br>Authors, I would suggest that this is not necessarily a strength in terms of research design but could be viewed as

~~randomisation with the external validity of routine clinical 418 practice.~~<br><br>~~Because patient inclusion, follow-up, and outcome assessment are embedded within routine clinical 419 practice, this design facilitates large-scale recruitment, standardised data collection, and long-term follow-up 420 while reducing additional burden on patients and healthcare providers. RRCTs are therefore particularly well 421 suited for pragmatic clinical research and may enhance the generalisability of findings to real-world practice. 422 However, the pragmatic nature of the design may provide less control over treatment delivery and follow-up 423 procedures than a traditional explanatory RCT~~	an "asset" to this study, in that the authors have integrated a RCT into a multicenter surgical and rehabilitation registry. This registry links the centers in multiple ways, which in turn may put less burden on the participating surgeons and therapists as patient recruitment/follow-up, data collection, and outcome assessments are embedded in the Registry's system. The down-side of use of the Registry may provide less control over treatment delivery and follow-up 423 procedures than a traditional explanatory RCT
Ln 424 ~~Several limitations should be considered~~. ~~First, the~~ The design of this study includes no blinding of the treating surgeons, therapists and participants, which ~~unblinded design~~ may introduce performance bias lending to awareness of . , ~~as 425 treating surgeons, therapists and participants are aware of~~ treatment allocation. ~~, potentially influencing 426 postoperative management and rehabilitation~~.	Ln 424 Authors, please consider edits. Try to eliminate repeated thoughts, simplify by condensing.
Ln 440 [insert multiple surgeon here] ~~Allowing surgeons to~~ For this study, surgeons continued to use their preferred materials and repair techniques. In doing so, this duplicates routine clinical practice and enhances 441 external validity.<br><br>Ln 441 The authors are aware that surgeon ~~However,~~ variation in surgical materials and technique, suture configuration and repair strength may 442 influence both rupture risk and ~~functional~~ measured outcomes.<br><br>Previous FPL zones I-II case series (references) have demonstrated declining 443 rupture rates with progressively stronger multistrand core suture repairs when combined with EAM. Historically, flexor tendon repairs involving the fingers with improved suture materials and repair techniques have also enhanced the safety of EAM.(current references) ~~suggesting that 444 repair technique is an important determinant of the safety of EAM5,6.~~ It remains unknown the effect of these improved materials and surgical repair techniques will have on the postoperatively immobilized flexor tendon repair in this study. ~~Consequently, surgical factors may 445~~	Ln 440 - Authors, please insert the surgeon discussion here followed by therapist, more in order of process.<br><br>Authors-How is external validity enhanced- example, please<br><br>Ln 441-445 consider edits

~~contribute to variation in outcomes both within and between centres~~.<br><br>~~Ln 445 To address this, detailed surgical data 446 will be collected and considered in adjusted analyses~~.	Ln 445 Authors- KEY POINT This is method, but be sure the methodological detail of comparing primary outcome/secondary outcomes with surgeon repair/technique for both groups is included in the Method section
Ln 426 ~~In addition, r~~There are standardised ~~rehabilitation protocols~~ rehabilitation that will be delivered by multiple hand therapists at ~~across~~ each participating centres. As a result, because treatment is patient-centered, there may be ~~, which may result~~ in some variation in treatment delivery and program progression~~., 428 despite the use of standardised rehabilitation protocols.~~ Collection of range of motion, maybe be effected by assessment bias since ~~is also possible as outcome 429~~ assessors are not blinded and are typically the therapist responsible for the participant's rehabilitation.	Ln 426-429 Consider edits
430 Understandably only direct visual inspection can confirm, rupture, however for this study, we have defined rupture as ... (Authors please define) ~~However, the~~ primary outcome, tendon rupture, we have defined rupture for this study as .... ~~using predefined clinical criteria and, when 431 applicable, surgical confirmation, which may reduce the risk of outcome misclassification.~~ To avoid misclassification ... ~~Nevertheless, some 432 clinically diagnosed ruptures that are not surgically confirmed may represent impaired thumb IP joint flexion 433 caused by tendon adhesions rather than true tendon rupture.~~	Authors- KEY POINT please clarify your definition for this study of tendon rupture and what you consider not a rupture and therefore not counted as such.(see ln 432-433.
Ln 434 ~~The secondary outcomes, including active range of motion and strength measurements, are more susceptible 435 to detection bias, because they are assessed by the treating hand therapist rather than an independent 436 assessor. Furthermore, some measurement variability is unavoidable despite the use of standardised 437 assessment protocols and calibrated instruments. Finally, PROMs may be affected by reporting bias, as 438~~ participants are aware of their treatment allocation. Although	Ln 434-439 Authors, this section is repetitive and uses vague terminology. Please revise to clarify and make it specific to your study, instead of general statements.<br><br>47

validated and standardised questionnaires are 439 used, reporting bias cannot be excluded	
Ln 447 ~~Although the rehabilitation protocols were developed collaboratively by hand surgeons and hand therapists 448 from all participating centres to promote consistency, adherence to the protocols may vary between 449 therapists, centres, and individual participants. Such variation may influence treatment effects and contribute 450 to outcome variability~~.	Ln 447-450 Authors, again this is repetitive and general, either delete or make pertinent to your study.
Ln 451 The interim analyses are intended primarily to ensure participant safety and to identify substantial treatment 452 effects at an early stage of the trial. More stringent statistical significance thresholds will be applied at interim 453 analyses than at the final analysis. This reduces the probability of incorrectly concluding that a treatment 454 effect exists based on random variation while maintaining the overall type I error rate of the trial.	Ln 451-454 reconsider if this is required in your discussion, please.
Ln 455 ~~If four weeks of cast immobilisation following FPL repair is associated with lower rupture rates without adversely affecting functional or patient-reported outcomes, the findings may support broader adoption in 457 postoperative rehabilitation. Conversely, if controlled splint/EAM achieves comparable rupture rates while 458 maintaining or improving functional recovery and patient-reported outcomes, the current rehabilitation 459 approach may be further supported. In either case, the results of this trial are expected to provide important 460 evidence to guide clinical postoperative management following FPL repair.~~	Ln 455- 460 Authors, go back to your purpose- a comparative study. Recall that your study lacks the rigor of a RCT, so you can not extrapolate findings to other situations.<br>You have not considered the effect of the surgeon's repair and the materials used as a factor that might contribute to rupture, or zone of injury, since zone II FPL repairs have a greater tendency to rupture, or patient adherence etc.<br><br>An important question for the Authors, is your motivation to do an internal audit of current practice to review the outcomes after immobilization or EAM interventions after Zone I-II repairs?
Ln 461 In conclusion, this multicentre RRCT, will be conducted across five hand surgery centres through a national 462 registry, with the intent to provide ~~the first direct~~ comparison of cast immobilisation and splint/EAM following primary zone 463 1-2 FPL repair. The study will evaluate tendon rupture rates, range of motion, grip and pinch strength, and 464 patient-reported outcomes ~~at~~ pre-operatively and at three and 12 months postoperatively.	Ln 461 Authors consider edits
**References**	**References- please continue to update as you revise**

**Figure 2**	Authors- please include the outcome measures taken preoperatively into this chart.
**Supplemental Materials**<br>**Table 1**<br>Table 1. Postoperative ~~Initial~~ wrist-thumb position in the dorsal cast for both groups, and ~~in~~ the thermoplastic volar splint for the ~~(Splint/~~EAM group fitted ~~2–5 days postoperatively.~~<br><br>**Table 2.** Immobilisation group: Rehabilitation timeline starting postoperative day 2–5 ~~days~~ to 4 weeks after repair. ~~Postoperatively~~<br><br>**Table 3.** ~~Splint/~~early active motion (EAM) ~~(Splint/EAM)~~ group: Rehabilitation timeline starting postoperative day 2–5 ~~days~~ to 4 weeks after repair ~~postoperatively~~<br><br>Authors on the supplemental material consider omitting those crossed off in red	Thank you for revising this information

Table 3. Splint/early active motion (Splint/EAM) group: rehabilitation timeline, 2–5 days to 4 weeks postoperatively

Time point	Clinic visit	Cast/splint status	Exercises	Permitted loading
2–5 days	Yes (splint fitting)	Dorsal cast adjusted, volar removable thermoplastic splint fitted (worn between training sessions; see Table 1 for position)	Passive thumb flexion; gentle active thumb flexion (≤50% of full range); active thumb extension using splint as support. 10–20 repetitions, every 2 hours, 6 times daily	No weight-bearing
2 weeks	Yes (compulsory)	Sutures removed; cast/splint adjusted as needed	Exercises continued 6 times daily, 10–20 repetitions per session; range of motion increased to two-thirds of full range	No weight-bearing
3 weeks	No (optional, on request)	Splint continued	Frequency increased to 6–12 sessions daily (hourly), 10–20 repetitions; gentle active isolated flexion with proximal stabilisation introduced if needed	No weight-bearing
4 weeks	Yes (compulsory)	Splint discontinued	Movement training continues per shared protocol (Table 4)	Very light activities only (as for immobilisation group); no weight-bearing, carrying or lifting

Table 2. Immobilisation group: rehabilitation timeline, 2–5 days to 4 weeks postoperatively

Time point	Clinic visit	Cast/splint status	Exercises	Permitted loading
2–5 days	Yes (cast check)	Dorsal cast assessed; worn at all times (see Table 1 for position)	None – full immobilisation	None
2 weeks	Yes (compulsory)	Sutures removed; cast assessed and adjusted as necessary	None – continued immobilisation	None
4 weeks	Yes (compulsory)	Cast removed	Movement training initiated (see shared protocol, Table 4); protective thumb and/or wrist orthosis may be provided up to 6 weeks for high-risk activities	Very light activities only (e.g., typing, holding utensils, light dressing, holding a glass); no weight-bearing, carrying or lifting

For peer review only

Elliot and Southgate 2005 article Table 1

TABLE 1. **Summary of Major Studies of Primary Repair of the FPL Tendon, 1989–2004**

Authors	*Rehabilitation Technique*	*N*	*Zones*	*Study Period (years)*	*Excellent and Good Results*	*Mechanical Rupture Rate*
Percival and Sykes (1989)[35]	(1) Immobilized	25	1, 2, 3	4	44% (White)	8%
	(2) Kleinert mobilization	26	1, 2, 3		60% (White)	8%
Noonan and Blair (1991)[33]	Kleinert mobilization	30	1, 2, 3, 4, 5	6	71% IP joints normal<br>82% metacarpophalangeal joints normal	—
Nunley et al. (1992)[34]	Kleinert mobilization	38	1, 2	2	Average IP 35%	3%
Thomazeau et al. (1996)[36]	(1) Immobilization	10	1, 2, 3, 4, 5			
	(2) Kleinert mobilization	3	1, 2, 3, 4, 5	5	85% (Tubiana)	5%
	(3) Passive mobilization	7	1, 2, 3, 4, 5			
Sirotakova and Elliot (1999)[50]	(4) Early active mobilization (group 1)	30	1, 2	8.5	70% (White) 73% (Buck-Gramcko)	17%
	(2) Early active mobilization (group 2)	39	1, 2		67% (White) 72% (Buck-Gramcko)	15%
	(3) Early active mobilization (group 3)	49	1, 2		76% (White) 80% (Buck-Gramcko)	8%
Kasashima et al. (2002)[28]	(1) Immobilization	16	1, 2, 3	12	50% (JSSH 1994)	0%
	(2) Kleinert mobilization	13	1, 2, 3		77% (JSSH 1994)	0%
Peck et al. (2003)[107]	Kleinert mobilization	23	1, 2	1.5	Not reported	4%
Baer et al. (2003)[55]	Mantero early active mobilization	22	1, 2	6	91% (Buck-Gramcko)*	0%
Sirotakova and Elliot (2004)[53]	Early active mobilization	48	1, 2	3.5	73% (White) 77% (Buck-Gramcko)	0%

IP = interphalangeal; JSSH = assessment of the Japanese Society for Surgery of the Hand (similar to the White assessment, uses IP range of motion only).
*This figure includes results of both finger and thumb flexor repairs. Although the authors indicate that the results were poorer in the thumbs, they only give an overall result of excellent and good results for all cases.

VERSION 3 - AUTHOR RESPONSE

Response to Reviewer Comments

Manuscript bmjopen-2026-118506.R2

Immobilisation versus Early Active Motion Following Primary Flexor Pollicis Longus Tendon Zones I-II Repair: Protocol for a Registry-Based Multicentre Randomised Controlled Trial in Sweden

We thank the reviewer for a thorough and constructive third review. Below we respond to each comment in turn.

Title

Reviewer comment	Author response
"Authors – consider edit"	We have added Zones I-II to the title as suggested.

Abstract

Reviewer comment	Author response
Ln 10–14: Please re-write the abstract Introduction/purpose with supporting background information.	We have chosen to retain the current wording of the abstract's Introduction, as we believe it already provides sufficient background to justify the study's rationale within the space constraints of a structured abstract.
Ln 15–25: Please revise the abstract Methods section and include the analysis, which was omitted.	We have added a sentence describing the planned analysis: intention-to-treat chi-square test with absolute risk difference and relative risk (95% CI) for the primary outcome, and t-tests / Mann-Whitney U / GEE for secondary outcomes.

Article Summary (Strengths and limitations)

Reviewer comment	Author response
Ln 36–48: General – consider edits and suggestions.	The bullet points have been revised as detailed below.
“Efficient” is the authors’ opinion only – strike.	“Efficient” has been removed.
The outcome tools and measurement methods did not always follow established standards; please avoid the term “standardised”.	Edited as suggested
Ln 41: Please consider separating the sample-size and rehabilitation-protocol/PROM characteristics into different points.	We agree that this point currently combines two related but distinct methodological strengths. However, the journal's format restricts the "Strengths and limitations" section to a maximum of five bullet points, which does not allow us to add an additional point without removing another. We have therefore retained the two aspects within a single bullet but revised the wording.
Ln 42: These factors demonstrate consistency rather than “strengthening methodological quality” per se.	The wording is revised
Ln 44: Please do not mix unrelated factors (blinding infeasibility and adherence monitoring).	We have kept these as a single bullet but linked them explicitly through a shared “as in routine clinical practice” framing rather than presenting them as a list of unrelated limitations.
Ln 45 / Ln 46: Is non-monitoring of adherence / non-standardisation of surgical technique a strength, weakness, or simply similar to clinical practice?<br><br>Ln 46 • In this Swedish system, surgical repair techniques and suture materials are not standardised across centres.	Both bullets now explicitly frame these features as reflecting routine clinical practice (“as in routine clinical practice...”).<br><br>We would prefer to keep the original wording rather than framing this as specific to "this Swedish system." Lack of standardisation in surgical technique and suture material across centres is a general feature of pragmatic multicentre trials, not something particular to the Swedish healthcare setting — the same limitation would likely apply in most other countries' trial systems as well. Attributing it to the Swedish system specifically could therefore be misleading to readers.
Ln 47–48: Please consider combining this thought with lines 44–45.	The adherence bullet and the surgical-technique/detection-bias bullet now use parallel “routine clinical practice” language so the two points read as a connected pair, while remaining separate bullets for clarity of the two distinct sources of bias (performance/adherence vs. detection).

Introduction

Reviewer comment	Author response
Ln 54–55: Consider edits.	The text has been revised.
Ln 59: Consider edits.	The text has been somewhat revised.
Ln 63–64: Please review Elliot & Southgate (2005), who found the 17% rupture rate applied to their oldest technique (group 1); rupture rate fell to 0% after modification of suture technique and materials (group 4). Please ensure the Introduction reflects this.	This sentence is intentionally kept as a brief, general statement to establish that elevated FPL rupture rates are a recognised problem in the literature; we do not intend to give a full account of technique-dependent results in the Introduction, as the possible effect on rupture rate of the surgical technique is discussed in the Discussion, which we consider the more appropriate location for that level of methodological detail.
Ln 64: Is “immobilisation could hypothetically represent a safer alternative” the authors’ opinion, or does it need a citation/rationale?	Our intention was not to express an opinion, but to present the underlying clinical hypothesis that motivates the study. We have revised the wording to make this clearer by explicitly framing immobilisation as a hypothetical alternative rather than a conclusion or recommendation.

Reviewer comment	Author response
Ln 65: Did Elliot & Southgate (2005) actually support the statement about "differences in tendon excursion, biomechanics and functional demands of the thumb"? (The reviewer notes the article instead discusses FPL muscle-shortening tension and zone 2 vascularity as explanations for higher rupture rates.)	We have revised the text to more accurately reflect the content of the cited article.
Ln 66–68: Please cite primary sources, not only reference 12; rupture rates have decreased since Kasashima et al. (2002). Key point: please revise the rationale for this study.	We have added Kasashima T, Kato H, Minami A. Hand Surg. 2002;7(2):171–176 as new reference.<br><br>We respectfully believe that the current rationale appropriately reflects the existing evidence gap and the hypothesis underpinning the present trial. While early active mobilisation has been widely adopted following flexor tendon repair, there are no studies directly comparing immobilisation with early active mobilisation specifically after primary FPL repair. Consequently, the optimal postoperative rehabilitation strategy for FPL repairs remains uncertain. As this uncertainty provides the scientific justification for the present study, we have retained the rationale.
Ln 67: "Delete".	Please see answer for line 64 above
Ln 68: "Delete".	We respectfully prefer to retain the original wording (however, we have combined the two sentences into one). The purpose of the present study is to compare two specific postoperative rehabilitation strategies rather than to identify an "optimal" rehabilitation programme in general.
Objectives, Ln 72–78: Consider edits.	The current text is " This study aims to compare the rupture rate after zones 1-2 FPL primary repair between patients who are immobilised for four weeks in a cast with patients who are issued a removable protective splint and instructed in controlled EAM two to five days postoperatively"<br>It does not match the reviewers text: "Ln 72-78 This study aims to compare the rupture rate after zones 1-2 FPL primary repair between patients who are 73 immobilised for four weeks in a cast with patients who at 2-5 days postoperatively are issued a removable protective splint and 74 instructed in controlled EAM exercises."<br>No change to content was done since the objectives already reflect the current design.
Ln 74: Consider edit (primary hypothesis wording).	Thank you, "Statistically lower rupture rate" has been changed to "statistically significantly lower rupture rate".
Ln 76: Consider edits (secondary hypotheses).	Thank you for this suggestion. We have chosen not to substantially revise this sentence.

Methods: Participants, Interventions and Outcomes

Reviewer comment	Author response
Study setting, Ln 93–96: Consider edits.	Edits have been done.
Intervention, Ln 122 / Ln 129: Consider edits. Key point: please add the supplemental material to the described intervention section – it is missing.	Thank you for this suggestion. However, we have chosen to retain most of the original wording. We have added a more explicit cross-reference to the supplemental material (supplemental material (Tables 1-4)")
Measurement of outcomes: The authors have not discussed evaluation of the primary outcome – tendon rupture. Please define what is considered a	We have added an explicit operational definition: The same definition, and its limitation, is repeated and discussed in the Discussion (assessment-bias paragraph).

Reviewer comment	Author response
rupture and what is not; the reviewer does not endorse calling a tendon ruptured based on lack of progression of active tendon glide alone – it needs to be visualised, to allow comparison of rupture rate with other studies.	
Ln 143: Key point – please consider converting AROM measures to White and/or Buck-Gramcko scoring to enable comparison with other studies.	Thank you for this suggestion. We have considered converting the AROM measurements to White and/or Buck-Gramcko scores. However, we have chosen not to do so, as range of motion is not a primary outcome of the present study. Furthermore, White and Buck-Gramcko are composite ordinal scoring systems derived primarily from range of motion and therefore provide less detailed information than reporting the individual AROM measurements directly. We believe that presenting the objective AROM values is more transparent and clinically informative while still allowing comparison with previous studies that report range of motion.
Ln 147: Please detail exactly how AROM is measured – isolated MCP/IP joint motion, or composite motion?	We have clarified that MCP and IP joint active range of motion is "measured in isolation at each joint."
Presenting AROM as a percentage of the opposite thumb is good, but please also add the White/Buck-Gramcko method.	Addressed in the Ln 143 comment above.
Ln 151: Consider edits.	The text has been edited.
Ln 153: How often are the dynamometers calibrated – prior to the study, or on a regular basis? The reviewer suggests calibration is most important for within-centre repeatability over time (comparing each patient to themselves) as well as between-centre comparability.	We have clarified in the manuscript that the dynamometers will be calibrated prior to the study and at regular intervals thereafter. Regular calibration is intended to ensure measurement consistency and reliability throughout the study, both within and across participating centres.
Ln 156–174: Consider edits.<br>Authors- KEY POINT Likert scale data (symptoms and satisfaction) can be analyzed without having a MCID established, please be sure to address in data analysis section	The text has been edited.<br>We agree that the absence of an established MCID does not preclude statistical analysis of the Likert-scale and other ordinal symptom/satisfaction measures. We have clarified this in the Statistical plan line 298-301. We have retained the statements noting the absence of an established MCID for each instrument, as this remains relevant to the clinical interpretation of effect sizes, but this is now clearly distinguished from the feasibility of the statistical comparison itself.

Adherence

Reviewer comment	Author response
Ln 213: Consider edits.	No further change identified as necessary. As the reviewer requested consistent use of "splint/EAM group" in the previous round, we have retained this naming throughout to avoid reintroducing inconsistency.
Ln 214: Are participants instructed to return if there are cast issues? Are both groups instructed on hand-use limitations, or only the immobilisation group? Authors- why are you directing this statement toward the immobilization group only, wouldn't this be the same for the EAM group, too?	We have added a sentence clarifying that participants in both groups are instructed on permitted hand use and load restrictions appropriate to their allocated protocol (with a cross-reference to Supplemental Tables 2 and 3) and are instructed to contact the treating centre promptly for cast- or splint-related problems.

Reviewer comment	Author response
	We agree that adherence is relevant to both groups and have revised the text to clarify the distinction rather than implying it is a concern for the immobilisation group alone.

Methods: Assignment of Interventions

Reviewer comment	Author response
Ln 226: Consider edit from here forward.	Se comment above regarding line 213

Methods: Data Collection, Management and Analysis

Reviewer comment	Author response
Ln 247: If a participant self-discharges at any point from 1 to 11 weeks postoperatively, will the data still be analysed?	Yes – as stated, data collected up to the point of withdrawal is retained and analysed; we have left this statement as written.

Discussion

Reviewer comment	Author response
Ln 397: The reviewer questions the claim that evidence for EAM is based primarily on observational studies/extrapolation, given Table 1 of Elliot & Southgate (2005).<br>Ln 399: The reviewer disagrees with the generalised statement that FPL rupture rates are higher than finger flexor rates, noting that with stronger repairs FPL rates have fallen since 2002 and may now be lower; suggests focusing the argument on the absence of comparative studies.<br>Ln 400–402: Please clarify or consider deleting the sentence on adhesion reduction and suture technique development.<br>Ln 402–406: Consider edits.	We agree that reported FPL rupture rates vary across studies and have likely improved over time with stronger repair techniques. We have revised the Discussion to soften the comparative statement accordingly (now noting that rates "have varied across studies, with some series reporting higher rates" rather than a generalised claim), and to move directly to our central point: that no study has directly compared immobilisation and EAM following FPL repair, which is the knowledge gap this RRCT was designed to address.
Ln 407: Consider all edits.	The paragraph has been revised. We have not added “preoperatively” since Active range of motion and grip/pinch strength are not assessed preoperatively (due to acute FPL laceration).
Ln 410: Outcomes affected by rupture could also include satisfaction and pain, not only “poor function.”	The sentence now reads “may be associated with poorer functional recovery, satisfaction, and higher levels of pain,” broadening beyond function alone.
Ln 412: Key point – AROM data needs conversion to White/Buck-Gramcko scoring to compare with other studies.	As explained in our response above, we have chosen not to convert AROM data to the White/Buck-Gramcko scoring system
Ln 413–14: Repetitive – please strike.	The sentence is removed.
Ln 415–423: Consider deleting/revising – the reviewer suggests this is better framed as an “asset” (integrating a RCT into a multicentre registry, linking centres and reducing burden on surgeons/therapists) than a methodological “strength” per se, and that reduced control over treatment delivery is a downside of the registry approach.	The paragraph has been rewritten

Reviewer comment	Author response
Ln 424: Please condense – avoid repeated thoughts. Ln 440: Please insert the surgeon-technique discussion before the therapist discussion, in order of clinical process. How is external validity enhanced – please give an example.	The limitations paragraph has been shortened and reorganised
Ln 441–445: Consider edits.	The surgical-technique paragraph has been rewritten
Ln 445: Key point – the comparison of primary/secondary outcomes with surgeon repair/technique for both groups should be described in the Methods, not only the Discussion.	The Statistical Plan already specifies that suture technique and core suture configuration are examined as covariates if baseline imbalance is observed; we have not duplicated this in the Discussion beyond referencing that detailed surgical data will be collected for both groups and considered in adjusted analyses.
Ln 426–429: Consider edits.	This paragraph has been shortened and folded into the revised limitations section
Ln 430–433: Key point – please clarify the definition of rupture for this study, and what is not considered a rupture.	Addressed via the same operational definition added to Measurement of Outcomes (see above), repeated in the Discussion: “tendon rupture is defined as loss of active IP joint flexion against resistance, confirmed by direct visualisation of tendon discontinuity when reoperation is performed…”
Ln 434–439: Repetitive and uses vague terminology; please make specific to this study rather than general statements.	This paragraph has been condensed and tightened, removing repeated references to “standardised” protocols/questionnaires and stating the detection- and reporting-bias risks once each, specific to this trial’s design.
Ln 447–450: Repetitive and general; please delete or make pertinent to this study.	This paragraph has been condensed and included in the section of performance and assessment bias
Ln 451–454: Please reconsider whether this level of interim-analysis detail is required in the Discussion.	This has been shortened substantially to a single sentence noting that interim analyses (described in the Methods) are intended to protect participant safety rather than to guide early efficacy claims, removing the duplicated methodological detail.
Ln 455–460: Please return to the study’s purpose – a comparative study that lacks the rigour of a fully controlled RCT, so findings cannot be broadly extrapolated. The reviewer notes that surgeon repair technique/materials, zone of injury (zone 2 has a greater tendency to rupture), and patient adherence have not been addressed as contributing factors. The reviewer also asks whether the authors’ underlying motivation is an internal audit of current practice.	The implications paragraph has been rewritten to state explicitly that, because surgical technique is not standardised, findings should be interpreted as applicable to routine Swedish practice rather than extrapolated broadly, and to name surgeon technique, zone of injury, and patient adherence as factors that may also affect rupture risk and are not the focus of this specific comparison.<br>We have not reframed the study as an internal audit, as it remains a prospective, registry-based randomised controlled trial (RRCT), not an internal audit of current practice
Ln 461: Consider edits.	Edited.

References

Reviewer comment	Author response
Please continue to update references as you revise.	We have added Kasashima T, Kato H, Minami A.

Figures and Supplemental Material

Reviewer comment	Author response
Figure 2: Please include the outcome measures taken preoperatively in this chart.	The SPIRIT diagram (Figure 2) include the preoperative outcomes (HQ-8 and QuickDASH assessments)
Supplemental Table 1: cast/splint position wording marked up for correction.	Edited as suggested.

Reviewer comment	Author response
Authors – for the supplemental material, please consider omitting the items crossed off in red.	Edited as suggested.

VERSION 4 - REVIEW

**Reviewer**	**2**
**Name**	**Howell, Julianne W.**
**Affiliation**	**Self-employed Hand Therapy Consultant**
**Date**	**28-Jul-2026**
**COI**	

Thank you authors for revising your manuscript again. It was a pleasure to read. There are a few minor edit suggestions, please consider. I happily accept this manuscript pending independent statistical review, please.

**bmjopen-2026-118506.R3**	**Reviewer Comments**
Title: Immobilisation versus Early Active Motion Following Primary Flexor Pollicis Longus Tendon Zones 1-2 3 Repair: Protocol for a Registry-Based Multicentre Randomised Controlled Trial in Sweden	
Abstract:	Reads well, thank you
Article Summary:<br>A prespecified sample size calculation based on registry data ensures the study is adequately 44 powered, while shared rehabilitation protocols and validated patient-reported outcome measures 45 ~~ensure~~ support methodological consistency across participating centres.	Authors- regarding blinding, you have mentioned the limitations, however the statistician will be blinded. Is this worth a mention?
Introduction<br>Rehabilitation after flexor tendon repair ~~injuries of the fingers~~ is critical to prevent tendon adhesion and joint 57 stiffness1,2<br>Conversely, EAM has been advocated to reduce tendon 71 tethering, adhesion formation and loss of interphalangeal (IP) joint motion following FPL repair12,13	Authors- is tendon tethering and adhesion formation the same? Consider deleting one
Methods:<br>Patient post-surgical follow-up is done using online follow-up 96 questionnaires via the national healthcare platform 117716. These patient-reported questionnaires query symptoms, functional impairment and satisfaction, and are done prior to surgery and again postoperatively at three 97 and 12	57

months. ~~of patients' self-reported symptoms, functional impairment, and satisfaction. In addition, the 98 participating centres routinely perform extended follow-up by hand therapists at 3 and 12 months 99 postoperatively~~ For patients undergoing flexor tendon repair, additional measures are done by the hand therapist at 3 and 12-months, which include range of motion and 100 grip strength.	
Intervention:<br>or 2) wrist and thumb dorsal cast and a volar removable 129 thermoplastic splint and implementation of controlled EAM (splint/EAM) within two to five days after surgery 130 (splint/EAM group).<br><br>Ln 143 The primary outcome variable is the frequency of FPL tendon rupture over a 12-month~~s~~ period.<br><br>Ln 153-157 Goniometric and Dynamometer measures	Authors, please include which joints are included in the thermoplastic splint- i.e., wrist-hand-thumb (refer to supplementary Table 1?)<br><br>Authors- please add that these will be taken in the standard position as described by (reference). [For repeatability]

Reviewer **3**

Name **Martin, James**

Affiliation **University of Birmingham, Institute of Applied Health Research**

Date **24-Aug-2026**

COI **None**

Thank you for this resubmission of your randomised trial protocol. I have some comments, listed below.

In the abstract and statistical plan, you say that the primary outcome will be analysed using the chi-square test. Given you plan to perform adjusted analyses, I wonder why you plan to report a p-value from a chi-square test, rather than from the result of a regression model. Could you clarify the rationale for this decision.

In the abstract, I would not focus on the hypothesis tests that might be performed, and only include the regression models that you plan to use. Of note, you do not report the models (or general framework) for the primary outcome to estimate the risk difference and relative risk, but you do report that you will use GEE for secondary outcomes.

Can you clarify how a measurement of the primary outcome (frequency of FPL tendon rupture) will be operationalised. For other outcomes, you are measuring them at 3months, and 12 months. But for the primary outcome, its dependent on complete loss of active IP joint flexion, and then confirmed at re-operation. So is this loss of joint flexion at the 3m or 12m period, or at any point between them?

The sample size statement was clear at first. The original sample size estimate of 194 patients per group was reproducible. However, the next part is not sufficiently transparent. The authors should clearly state what event rate was then assumed, and what allowance for loss to follow up was allowed for, in order for the 190 patients per group to be considered sufficient.

The randomisation is stratified by centre. It is recommended that any type of restricted randomisation used is reflected in the statistical analysis. As such, the planned analysis approach should include adjustment for centre in some way.

You say that: "The absolute risk difference and relative risk with corresponding 95% confidence intervals calculated using standard methods for binomial outcomes, will be reported." This is not sufficient reporting. You should clearly articulate the planned approach to obtain the relative risk and risk difference. Particularly whether you plan to use a GLM or GEE approach.

The following statement in the statistical methods confuses me: "If clinically relevant imbalance in baseline characteristics (e.g., age, sex, smoking, injury of dominant hand, suture technique and core suture configuration) is observed between groups, multivariable Poisson regression with robust variance estimation will be used to estimate adjusted relative risks." Firstly, chance baseline differences can occur despite valid randomisation, and so the proposed approach is problematic. You should not adjust for "unbalanced" characteristics, but rather should prespecify the covariates to adjust for in any analyses, otherwise you are selecting a final model after examining treatment groups. Moreover, "unbalanced" is subjective unless explicitly defined; if based on statistical significance, selection will depend heavily on sample size rather than clinical or prognostic importance. In a properly randomised trial, chance baseline differences do not ordinarily introduce confounding into the treatment comparison. Adjustment may improve precision, but covariates should be chosen a priori based on prognostic importance and/or their role in the randomisation procedure - not based upon observed between-group differences. CONSORT 2025 explicitly states that significance testing of baseline differences is not recommended and should not be reported and that adjustment decisions should not be based on statistically significant differences between randomised groups. Please therefore prespecify the covariates for the primary/secondary analysis model and their justification. Any additional adjustment prompted by an unexpected baseline difference should be presented as a sensitivity analysis and not the primary analysis.

To add, it is not clear how a modified Poisson regression model is different to the planned primary analysis – because the primary analysis model is not clearly specified.

As mentioned earlier, centre should be included for in the analysis of the primary outcome. I do note that it is included for secondary outcomes – so it's unclear why it was not included for the primary outcome. That being said, there is more information for analysis models for the secondary outcomes than there is for the primary outcome. This is something that I believe needs to be rectified.

For the missing data sensitivity analyses, is there a reason that other participant demographic information at baseline is not being included to aid the imputation process?

VERSION 4 - AUTHOR RESPONSE

We thank Dr. Martin for a thorough and constructive statistical review. We have revised the statistical analysis plan as detailed below, and updated the Abstract, Sample size, Measurement of outcomes and Statistical plan sections of the manuscript accordingly.

Reviewer 2 (Ms. Julianne W. Howell)

We thank the reviewer for her positive assessment and for accepting the manuscript pending independent statistical review.

Statistical review

Reviewer comment	Author response
In the abstract and statistical plan, you say that the primary outcome will be analysed using the chi-square test. Given you plan to perform adjusted analyses, I wonder why you plan to report a p-value from a chi-square test, rather than from the result of a regression model. Could you clarify the rationale for this decision.	We have revised the statistical analysis plan so that the primary outcome is analysed using a Mantel-Haenszel (MH) stratified analysis, stratified by centre, rather than an unstratified chi-square test. The MH-weighted relative risk and risk difference will be estimated with 95% confidence intervals. We chose MH stratification over a covariate-adjusted regression model because it is more robust to sparse strata given five participating centres and a relatively infrequent primary outcome. An unadjusted chi-square test is retained as a supportive descriptive statistic, not as the primary inferential analysis. The Abstract and Statistical plan sections have been revised accordingly (tracked changes).
In the abstract, I would not focus on the hypothesis tests that might be performed, and only include the regression models that you plan to use. Of note, you do not report the models (or general framework) for the primary outcome to estimate the risk difference and relative risk, but	We have revised the abstract to reflect the stratified analysis approach used for the primary outcome (a stratified analysis, stratified by centre) rather than referring to the chi-square test; the specific method (Mantel-Haenszel) is named in the Statistical plan section of the manuscript body. We have kept a brief mention of the methods used for secondary outcomes (GEE and standard tests for continuous and ordinal data), since BMJ Open's structured abstract format calls for concise identification of methods, but we have reworded the sentence to foreground the effect estimates

Reviewer comment	Author response
you do report that you will use GEE for secondary outcomes.	(with 95% confidence intervals) rather than the hypothesis tests themselves.
Can you clarify how a measurement of the primary outcome (frequency of FPL tendon rupture) will be operationalised. For other outcomes, you are measuring them at 3 and 12 months. But for the primary outcome, it is dependent on complete loss of active IP joint flexion, and then confirmed at re-operation. So is this loss of joint flexion at the 3m or 12m period, or at any point between them?	Tendon rupture is a clinical event rather than a scheduled assessment: it may present, and will be recorded, at any point during the 12-month follow-up period, not only at the 3- or 12-month visits. We have added a clarifying sentence to 'Measurement of outcomes' stating that participants are instructed to contact their centre if a rupture is suspected between scheduled visits (as already described under 'Adverse events and safety'), and that the date of clinical presentation or reoperation is recorded as the event date for the 12-month cumulative incidence used in the primary analysis.
The sample size statement was clear at first. The original sample size estimate of 194 patients per group was reproducible. However, the next part is not sufficiently transparent. The authors should clearly state what event rate was then assumed, and what allowance for loss to follow up was allowed for, in order for the 190 patients per group to be considered sufficient.	We have expanded the Sample size section to state that, because the overall event rate was expected to be higher than the HAKIR-based estimates (as up to one-third of clinically confirmed ruptures may not undergo reoperation and are therefore not captured in HAKIR), the number of evaluable patients required to detect the same absolute difference was correspondingly lower; a conservative target of 173 evaluable patients per group was calculated. Allowing for up to 10% loss to follow-up, a final recruitment target of 190 patients per group was set
The randomisation is stratified by centre. It is recommended that any type of restricted randomisation used is reflected in the statistical analysis. As such, the planned analysis approach should include adjustment for centre in some way.	Agreed. Centre is now included as a covariate in the primary analysis model, consistent with its existing inclusion in the secondary-outcome (GEE) models. This is described in the revised Statistical plan section.
You say that: "The absolute risk difference and relative risk with corresponding 95% confidence intervals calculated using standard methods for binomial outcomes, will be reported." This is not sufficient reporting. You should clearly articulate the planned approach to obtain the relative risk and risk difference. Particularly whether you plan to use a GLM or GEE approach.	We have clarified that the primary analysis for relative risk and risk difference will use a MH stratified analysis, stratified by centre, rather than a GLM or GEE approach (see first comment).<br>GEE is reserved for the secondary, repeated-measures outcomes, where within-subject correlation across follow-up time points must be accounted for. The primary outcome is assessed once per participant over the 12-month period and does not require a GEE approach.

Reviewer comment	Author response
The following statement in the statistical methods confuses me: "If clinically relevant imbalance in baseline characteristics ... is observed between groups, multivariable Poisson regression with robust variance estimation will be used to estimate adjusted relative risks." ... You should not adjust for "unbalanced" characteristics, but rather should prespecify the covariates to adjust for in any analyses ... CONSORT 2025 explicitly states that significance testing of baseline differences is not recommended ... Please therefore prespecify the covariates for the primary/secondary analysis model and their justification. Any additional adjustment prompted by an unexpected baseline difference should be presented as a sensitivity analysis and not the primary analysis.	We have removed the wording that tied adjustment to observed baseline imbalance. The primary analysis now stratifies only by centre, prespecified a priori as the randomisation stratification factor; no other covariates are included in the primary analysis, and no adjustment decision is based on post hoc comparison of baseline characteristics between groups.<br><br>Should a clinically important baseline imbalance nonetheless be noted after unblinding, we will report an additional analysis adjusting for the relevant baseline characteristics as an explicitly labelled sensitivity analysis, separate from the primary analysis, consistent with CONSORT 2025 guidance.
To add, it is not clear how a modified Poisson regression model is different to the planned primary analysis -- because the primary analysis model is not clearly specified. As mentioned earlier, centre should be included for in the analysis of the primary outcome. I do note that it is included for secondary outcomes -- so it's unclear why it was not included for the primary outcome.	This ambiguity has been resolved by the revisions described above: a centre-stratified Mantel-Haenszel analysis is now explicitly specified as the primary analysis for both relative risk and risk difference, removing the previous ambiguity between a chi-square-based primary analysis and a conditionally triggered Poisson model.
For the missing data sensitivity analyses, is there a reason that other participant demographic information at baseline is not being included to aid the imputation process?	No, and we agree that the imputation model should be richer. We have expanded the imputation model for secondary outcomes to include additional baseline characteristics (smoking status, injury of the dominant hand, suture technique and core suture configuration) in addition to age and sex, to strengthen the plausibility of the missing-at-random assumption.

VERSION 5 - REVIEW

**Reviewer**	**3**
**Name**	**Martin, James**
**Affiliation**	**University of Birmingham, Institute of Applied Health Research**
**Date**	**12-Sep-2026**
**COI**	

Thank you for addressing my comments, and I think the statistical elements are much clearer now. I still have a couple of minor comments though that I welcome a response to:

The proposed centre-stratified analysis appears appropriate given that randomisation was stratified by centre. However, please specify the exact Mantel–Haenszel estimators and weighting schemes used for the pooled relative risk and risk difference, including how much influence centres of different sizes will have and whether the weighting differs between the two effect measures.

Given the proposed approach, will the consistency of the treatment effect across centres be examined, either descriptively through centre-specific estimates or using a treatment-by-centre interaction? Though, given the small number of centres and expected low event frequency, any formal assessment of heterogeneity is likely to have limited power.

Please clarify how centres with zero events in one or both treatment groups will be handled (given the low event rate in both arms, its possible centres will have no outcomes) and how the 95% confidence intervals will be calculated.

The phrase “rather than an unstratified chi-square test” could be removed – as this doesn’t make sense within the text (though I understand that you have it in response to reviewer comments)

What is the purpose of still including the unadjusted chi-square test? Its not clear to me how it is a supportive descriptive statistic or the context in which it will be used.

VERSION 5 - AUTHOR RESPONSE

Reviewer 3 (Dr. James Martin)

Thank you for addressing my comments, and I think the statistical elements are much clearer now. I still have a couple of minor comments though that I welcome a response to.

Response: We thank the reviewer for the careful review and constructive comments.

1. The proposed centre-stratified analysis appears appropriate given that randomisation was stratified by centre. However, please specify the exact Mantel–Haenszel estimators and

weighting schemes used for the pooled relative risk and risk difference, including how much influence centres of different sizes will have and whether the weighting differs between the two effect measures.

Response: Thank you. The Statistical plan now specifies the MH estimators for the RR and RD, their weighting and the variance estimators used for the 95% confidence intervals. Under 1:1 allocation, the RD weights are approximately proportional to centre size. The RR weights also depend on the number of events in the reference (splint/EAM) group. Larger centres will therefore have greater influence on both estimates, and the weighting differs between the two measures, as now stated in the text.

2. Given the proposed approach, will the consistency of the treatment effect across centres be examined, either descriptively through centre-specific estimates or using a treatment-by-centre interaction? Though, given the small number of centres and expected low event frequency, any formal assessment of heterogeneity is likely to have limited power.

Response: We agree. Centre-specific numbers of ruptures and risk differences with 95% confidence intervals will be presented descriptively in a forest plot. As the reviewer notes, a formal treatment-by-centre interaction test would have limited power with five centres and a low event rate, and we have therefore not included one.

3. Please clarify how centres with zero events in one or both treatment groups will be handled (given the low event rate in both arms, its possible centres will have no outcomes) and how the 95% confidence intervals will be calculated.

Response: The MH estimators require no continuity correction. Centres with zero events in one group contribute to both estimates. Centres with zero events in both groups contribute no information to the RR but are retained in the RD. If no ruptures occur in one group across all centres, only the RD will be reported. Confidence intervals will be based on the Greenland–Robins (log RR) and Sato (RD) variance estimators, which are valid in sparse strata. This is now described in the Statistical plan.

4. The phrase "rather than an unstratified chi-square test" could be removed – as this doesn't make sense within the text (though I understand that you have it in response to reviewer comments).

Response: Thank you. The phrase has been removed.

5. What is the purpose of still including the unadjusted chi-square test? Its not clear to me how it is a supportive descriptive statistic or the context in which it will be used.

Response: We agree that the unadjusted chi-square test adds little beyond the stratified primary analysis and could cause ambiguity about which result is primary. It has been removed.
