# Supplementary material for: Immobilisation versus early active motion following primary flexor pollicis longus tendon zones 1–2 repair: protocol for a registry-based multicentre randomised controlled trial in Sweden
Source: BMJ Open. 2026 Oct 20;16(10):e118506. doi: 10.1136/bmjopen-2026-118506 (PMC13684173; doi:10.1136/bmjopen-2026-118506)
Supplement: Supplementary file 2 [file bmjopen-16-10-s001.docx]

# Registry-Randomized Study of Rehabilitation Regimen After Flexor Tendon Injury of the Thumb

## Information and invitation to participate in a research study

We would like to ask whether you would like to participate in a research project. This document provides information about the project and what participation in the study involves. Please read the information carefully before making your decision. Do not hesitate to ask questions if anything is unclear.

## Background and purpose of the project

This study is being conducted at hand surgery clinics in Sweden to compare outcomes after surgery for flexor tendon injuries of the thumb depending on the postoperative rehabilitation program.

After surgery, the thumb may either: (1) be completely rested (immobilized) in a cast for 4 weeks, or (2) begin early motion training of the thumb flexor tendon within 2–5 days. Even with early motion training, a splint/cast is worn between training sessions for protection.

At present, it is unclear how outcomes differ between these two rehabilitation regimens. The aim of the study is to compare the risk of tendon rupture and functional outcomes between the two regimens.

The project is conducted by researchers at the regional hand surgery centers in Linköping, Malmö, Stockholm, Uppsala, Umeå and Örebro. The research principal is Region Stockholm. The study has been approved by the Swedish Ethical Review Authority (reference number 2023-04922-01).

## Invitation to participate

We are inviting individuals aged 15 years or older who are undergoing surgery for a flexor tendon injury of the thumb at a hand surgery clinic in Sweden.

## How is the study conducted?

You have sustained a flexor tendon injury of the thumb and will undergo surgery to restore flexion. The surgical procedure is the same for all patients. In connection with surgery, you will be asked whether you wish to participate.

If you choose to participate, you will be randomly assigned to one of two rehabilitation regimens: (1) immobilization in a cast for four weeks, or (2) early motion training. Both rehabilitation regimens are well established and widely used.

After surgery, you will be followed within the Swedish National Quality Registry for Hand Surgery (HAKIR) through clinical assessments (thumb motion and strength) and patient-reported questionnaires (web-based survey via 1177) at 3 and 12 months. No samples will be collected for the study, and all examinations are painless. Follow-up is conducted according to routine clinical practice within HAKIR, and no additional visits or examinations are required.

## Possible consequences and risks of participation

Surgery and follow-up are performed according to standard clinical practice, and participation in the study does not provide any direct benefit to you. Early motion training may increase the risk of tendon rupture, whereas immobilization may increase the risk of thumb stiffness. Participation will help us determine which rehabilitation regimen provides the best results after surgery for flexor tendon injuries of the thumb. Future patients may benefit from improved care based on the study results. No samples will be taken, all examinations are painless, and no risk of personal injury is anticipated.

## What happens to my data?

Your examination results and questionnaire responses will be stored in HAKIR. For this project, data will be extracted from HAKIR in pseudonymized form (meaning that the data cannot be directly linked to you) for analysis. Your contact details, consent form and results will be stored securely so that unauthorized persons cannot access them. Results will be presented in a way that does not allow identification of individual participants.

Region Stockholm is responsible for the processing of your personal data. Under the EU General Data Protection Regulation (GDPR), you have the right to access your data free of charge and to request correction of any inaccuracies. You may also request deletion or restriction of processing of your personal data, although these rights may be limited when the data are necessary for the research. If you are dissatisfied with the handling of your personal data, you may file a complaint with the Swedish Authority for Privacy Protection.

## How will I receive information about the results?

The results will be published in scientific journals and presented at scientific conferences. If you would like to receive information about the scientific reporting, you may contact the study investigators.

## Insurance and compensation

Standard patient insurance applies in connection with surgery and follow-up visits.

## Participation is voluntary

Your participation is voluntary, and you may withdraw at any time. If you choose not to participate, or if you withdraw from the study, you do not need to provide a reason. Your decision will not affect your future care or treatment. If you wish to withdraw, please contact the investigator responsible for the project in your region.

## Project investigators

Stockholm: Maria Wilcke (Principal Investigator)
Linköping: Erika Nyman
Uppsala: Sara Edsfelt
Örebro: Eva Lundqvist
Umeå: Gustav Andersson
HAKIR Registry: Marianne Arner (Registry Holder)

## Consent to participate

I have received oral and written information about the study and have had the opportunity to ask questions. I may keep the written study information.

☐ I consent to participate in the study: Registry-Randomized Study of Rehabilitation Regimen After Flexor Tendon Injury of the Thumb.

☐ I consent to the processing of my personal data as described in the participant information.

Place and date: __________________________

Signature: _______________________________

Personal identity number and name: _______________________________
