# Supplementary material for: Immobilisation versus early active motion following primary flexor pollicis longus tendon zones 1–2 repair: protocol for a registry-based multicentre randomised controlled trial in Sweden
Source: BMJ Open. 2026 Oct 20;16(10):e118506. doi: 10.1136/bmjopen-2026-118506 (PMC13684173; doi:10.1136/bmjopen-2026-118506)
Supplement: Supplementary file 3 [file bmjopen-16-10-s002.docx]

**Supplemental material**

The following standardised rehabilitation protocols were used in the trial. The rehabilitation protocols were developed by a consensus group consisting of hand surgeons and certified hand therapists from all participating centres. All participating centres are instructed to follow these protocols. Additional appointments and minor adaptations are permitted when clinically indicated.

**Rehabilitation protocols**

**Table 1. Initial wrist and thumb position in the dorsal cast (both groups) and in the volar splint (Splint/EAM group, fitted 2–5 days postoperatively)**

| **Joint** | **Position** |
| --- | --- |
| Wrist | Slight extension (0–25°) |
| Carpometacarpal joint of the thumb (CMC-1) | Semi-abducted |
| Metacarpophalangeal joint of the thumb (MCP-1) | Resting position (0–25° flexion) |
| Interphalangeal joint of the thumb (IP) | 0–10° flexion |

**Table 2. Immobilisation group: rehabilitation timeline, 2–5 days to 4 weeks postoperatively**

| **Time point** | **Clinic visit** | **Cast/splint status** | **Exercises** | **Permitted loading** |
| --- | --- | --- | --- | --- |
| 2–5 days | Yes (cast check) | Dorsal cast assessed; worn at all times | None – full immobilisation | None |
| 2 weeks | Yes (compulsory) | Sutures removed; cast assessed and adjusted as necessary | None – continued immobilisation | None |
| 4 weeks | Yes (compulsory) | Cast removed | Movement training initiated (see shared protocol, Table 4); protective thumb and/or wrist orthosis may be provided up to 6 weeks for high-risk activities | Very light activities only (e.g., typing, holding utensils, light dressing, holding a glass); no weight-bearing, carrying or lifting |

**Table 3. Splint/early active motion (Splint/EAM) group: rehabilitation timeline, 2–5 days to 4 weeks postoperatively**

| **Time point** | **Clinic visit** | **Cast/splint status** | **Exercises** | **Permitted loading** |
| --- | --- | --- | --- | --- |
| 2–5 days | Yes (splint fitting) | Volar removable thermoplastic splint fitted; worn between training sessions | Passive thumb flexion; gentle active thumb flexion (≤50% of full range); active thumb extension using splint as support. 10–20 repetitions, every 2 hours, 6 times daily | No weight-bearing |
| 2 weeks | Yes (compulsory) | Sutures removed; cast/splint adjusted as needed | Exercises continued 6 times daily, 10–20 repetitions per session; range of motion increased to two-thirds of full range | No weight-bearing |
| 3 weeks | No (optional, on request) | Splint continued | Frequency increased to 6–12 sessions daily (hourly), 10–20 repetitions; gentle active isolated flexion with proximal stabilisation introduced if needed | No weight-bearing |
| 4 weeks | Yes (compulsory) | Splint discontinued | Movement training continues per shared protocol (Table 4) | Very light activities only (as for immobilisation group); no weight-bearing, carrying or lifting |

**Table 4. Shared rehabilitation protocol, both groups, 4–12 weeks postoperatively**

| **Time point** | **Clinic visit** | **Exercises** | **Permitted loading** |
| --- | --- | --- | --- |
| 4 weeks | Yes (compulsory) | Passive flexion (and, if needed, passive extension) of the IP joint with the MCP joint held in flexion; active flexion with stabilisation of the proximal phalanx; tendon-gliding exercises; full active flexion without force; active extension and palmar abduction; if needed, active wrist exercises (extension-flexion with relaxed fingers, ulnar-radial deviation, pronation-supination). 5–10 repetitions, 6 times daily | Very light activities only; no weight-bearing, carrying or lifting |
| 6 weeks | No (self-directed; additional visit if clinically indicated) | Continued thumb and finger exercises through full range of motion; assisted passive IP-joint extension with active hold if needed; wrist orthosis discontinued | Up to 1 kg; light, controlled activities permitted |
| 8 weeks | No (self-directed) | Continued thumb and finger exercises; dynamic strength training for wrist and grip with light resistance | Up to 2 kg |
| 10 weeks | No (self-directed) | Continued thumb and finger exercises | Up to 3 kg |
| 12 weeks | Yes (compulsory) | Continued mobility exercises as needed | Full weight-bearing in daily activities, with gradual increase in other loads |

For both groups, in-person follow-up visits are compulsory at 2, 4 and 12 weeks postoperatively (Tables 2–4); the 6-, 8- and 10-week progression points are self-directed by the patient according to the standardised protocol and do not require an additional clinic visit unless clinically indicated (e.g., difficulties with exercises due to pain, swelling or stiffness). Additional appointments may be conducted digitally or physically and are recorded according to the individual needs of the patient. Attendance at scheduled visits and any protocol deviations or additional rehabilitation sessions will be recorded. Progression to the next rehabilitation stage should be guided by clinical assessment and may be delayed in the presence of excessive pain, swelling, loss of active motion, signs of tendon irritation, or other clinical concerns.
